# Impact of Mindfulness on sleep quality in innovative corporate employees: A chain mediation of Social Interaction Anxiety and bedtime procrastination

**DOI:** 10.1371/journal.pone.0302881

**Published:** 2024-05-22

**Authors:** Junjian Zheng, Xiaojing Zang, Xiaowei Xu, Hanzhong Zhang, Wasi Ul Hassan Shah

**Affiliations:** 1 Police Physical Education and Research Department, The National Police University for Criminal Justice, Baoding, China; 2 Henan University, Institute of Physical Education, Kaifeng, China; 3 Henan Provincial People’s Hospital, Dermatology, Zhengzhou, China; 4 Zhejiang Shuren University, Physical, Aesthetic and Labor Education Centre, Hangzhou, China; 5 Institute of Physical Education, Kunming University of Technology, Kunming, China; 6 School of Management, Zhejiang Shuren University, Hangzhou, China; Alexandria University Faculty of Nursing, EGYPT

## Abstract

In the context of innovative enterprises in China, the significance of sleep quality for employees’ physical and mental well-being cannot be understated. This study explores the complex relationship between Mindfulness and sleep quality and examines the potential interaction between Social Interaction Anxiety and prolonged sleep behavior. To this end, a thorough evaluation involving the administration of the Mindfulness scale, Social Interaction Anxiety scale, sleep delay scale, and the Pittsburgh Sleep Quality Index (PSQI) was conducted among a significant sample of innovative enterprise employees (N = 1648). The findings reveal that a notable proportion of these employees, 31.1% to be precise (as per PSQI 8), grapple with compromised sleep quality. Subsequent analyses shed light on compelling patterns, underscoring a robust negative correlation between Mindfulness and factors like Social Interaction Anxiety, sleep delay, and sleep quality (β = -0.71, -0.37, -0.35; P < 0.01). Conversely, a significant positive correlation emerges connecting Social Interaction Anxiety, sleep delay, and sleep quality (β = 0.23, 0.37, 0.32; P < 0.01). Interestingly, mediation analysis demonstrates that Mindfulness significantly negatively influences sleep quality, independent of demographic factors such as sex and age. This impact is mediated by sleep delay, which also interacts with Social Interaction Anxiety. In summary, the research emphasizes the predictive function of Mindfulness in improving sleep quality among employees in innovative enterprises, achieved through its reduction of Social Interaction Anxiety and bedtime procrastination tendencies.

## 1. Introduction

Sleep quality stands as a pivotal determinant impacting an individual’s overall health. In 2022, approximately 509 million individuals in China were reported to have experienced sleep disorders, accounting for a prevalence rate of 38.2%. Particularly noteworthy is the heightened insomnia among employees within innovative enterprises [1]. The complex relationships between problematic social media use, emotional control, personality traits, and internet addiction (IA) are examined in this study. The study provides a thorough analysis by measuring social media addiction, fear of missing out, emotional (dys) regulation, and personality factors related to IA in a sample of 397 adults between 18 and 35. Pearson’s correlations show significant relationships between IA and the variables under investigation. Moreover, a mediation model clarifies essential facts regarding the critical role that emotional (dys) regulation plays in the link between problematic social media use and FOMO. These results aid in creating a fresh, integrated model meant to fully comprehend the characteristics, risk variables, and predictors related to IA [2]. Varchetta et al. [3], explore the complex interactions between social media addiction (SMA) and internet addiction (IA), as well as psychological factors such as personality traits, emotional dysregulation, and fear of missing out (FOMO). With a sample of 598 female university students in Spain, mostly 18 to 35, the study uses an online questionnaire to evaluate these constructs. Positive correlations between IA, SMA, FoMO, emotional dysregulation, and the neuroticism dimension of the Big Five personality traits are revealed by correlation analysis.

On the other hand, negative correlations are found between gender, the Big Five Conscientiousness component, SMA, and IA. Additionally, a mediation model reveals a positive and significant indirect influence via SMA and FoMO scores, explaining a robust overall effect of emotional dysregulation on IA scores. These results aid in creating a fresh, integrated model to fully comprehend the traits, indicators, and risk factors connected to IA.

Similarly, Mari et al. [4] examine gender differences in the development of pathological behaviors related to internet addiction. The research intends to analyze unique patterns of addictive behaviors in men and women by looking at variables such as internet gaming addiction, social media addiction, fear of missing out, and phubbing. Two hundred seventy-six people aged 18–30 participated in the study, with over half male. The study uses detailed online surveys to investigate the relationship between gender, psychological features, and internet addiction. It finds that gender plays a crucial role in determining individuals’ vulnerability to internet addiction, with distinct patterns observed in men and women. Social media addiction is a significant predictor for both genders, although regression analysis reveals unique variables for each gender. This comprehensive comprehension of the fundamental factors influencing the development of internet addiction has important implications for creating prevention and treatment strategies specifically designed to target these addictive behaviors effectively. In this context, innovation-oriented enterprises pertain to entities characterized by independent intellectual property rights, recognized brands, robust international competitiveness, and a strategic reliance on technological innovation to secure a competitive edge and ensure sustainable development. Such enterprises constitute the bedrock of an innovative nation and a fundamental component of China’s economic progress. However, a concerning trend has emerged—employees in innovative Chinese enterprises exhibit unhealthy sleep habits and encounter sleep-related issues, including truncated and delayed sleep durations, with the incidence of sleep disorders approaching 41.2% [5,6]. The ramifications extend beyond mere sleep, profoundly influencing not only the operational efficiency of innovative enterprise staff but also elevating the vulnerability to an array of psychological disorders [7,8]. In light of these considerations, the imperative of directing attention towards preemptive measures and targeted interventions for addressing sleep disorders among employees in China’s innovative enterprises assumes profound practical significance.

### 1.1 Mindfulness and sleep

The potential enhancement of sleep quality through Mindfulness has garnered considerable attention [9]. Mindfulness entails a deliberate state of awareness, wherein one consciously directs their focus towards the immediate objective, embracing experiences without judgment [10]. Research in this domain has underscored the association between Mindfulness and heightened sleep quality, positing that its cultivation can improve sleep patterns [11,12]. Similarly, studies examining the college student population have indicated a positive association between increased levels of Mindfulness and improved sleep quality among students [13]. Remarkably, individuals engaged in Mindfulness often report a sense of physical and mental relaxation achieved by directing attention to their breath, bolstering concentration. This relaxation process may encompass sequentially relaxing individual body parts, mitigating internal emotional turmoil, and fostering a preparatory state for sleep induction [14]. This phenomenon could be attributed to the notion that Mindfulness induces relaxation of the bodily and nervous systems, thereby diminishing stress responses—a pivotal aspect in sleep quality regulation [15]. The evidence suggests that elevated mindfulness levels are conducive to ameliorated sleep quality. Consequently, this study postulates the following hypothesis: H1: Engaging in mindfulness practice significantly influences employees’ sleep quality within innovative enterprises.

### 1.2 Mediating role of social interaction anxiety

Social anxiety refers to the anxiety experienced during social interactions due to anticipated social roles and behaviors [16]. However, individuals grappling with Social Interaction Anxiety often subject themselves to self-denial, leading to the emergence of negative emotions like anxiety and tension. These emotions can trigger arousal that disrupts sleep [17]. Additionally, such individuals may redirect their attention toward online communication, potentially fostering mobile phone or Internet addiction that detrimentally affects sleep patterns [18]. This form of anxiety is characterized by an underlying fear of negative evaluation, often culminating in a tendency to avoid social situations. In cases where avoidance isn’t possible, enterprise staff members frequently contend with anxiety and physiological reactions that infringe upon their daily social interactions and sleep health. Given the dual stressors of homeownership and livelihood that innovative enterprise employees in China face, they are prone to heightened anxiety levels, underscoring the imperative for psychological researchers to address their mental well-being and sleep quality [19]. Empirical evidence indicates a substantial prevalence of elevated Social Interaction Anxiety levels among innovative enterprise employees, with 27.2% exhibiting pronounced anxiety and an additional 14.1% contending with notable fear, often coupled with varying degrees of sleep disorders [20].

Mindfulness practice emerges as an avenue for stress alleviation and nervous system relaxation, achieved through bodily relaxation techniques [21]. Prior investigations have highlighted the potential for mindfulness practice to mitigate depressive symptoms and Social Interaction Anxiety among college students [22]. Notably, college students contending with Social Interaction Anxiety also frequently exhibit varying degrees of sleep disorders.

There is a notable scarcity of research investigating the correlation between Social Interaction Anxiety and sleep quality among innovative employees, both domestically and internationally. The unique context of the corporate environment and culture in which they operate introduces distinctive variables that might significantly impact sleep quality [23]. To this end this study posits hypothesis H2: Within the cohort of innovative corporate employees, Social Interaction Anxiety is a mediating factor between Mindfulness and sleep quality.

### 1.3 Mediating role of bedtime procrastination

Sleep delay pertains to the intentional postponement of sleep, lacking external impetus, and is characterized by "retiring to bed later than intended for no apparent reason" [24,25]. Extensive prior research has established connections between sleep delay and sleep quality, encompassing associations with depression, mobile phone addiction, anxiety, insomnia, and other related factors [26–28]. Concurrently, Hairston and Shpitalni’s work has uncovered links between sleep delay and difficulty falling asleep [29]. Additionally, Sirois’s findings have underscored the multidimensional nexus between delayed sleep, encompassing diminished sleep duration and compromised sleep quality, alongside its interplay with emotional states such as anxiety, depression, and social disorders [27,30].

The role of poor sleep habits in engendering suboptimal sleep quality is well-documented, with Mindfulness posited as a potential regulator of such habits. Specifically, the procrastinatory behavior of delaying bedtime directly truncates sleep duration, thereby negatively impacting sleep quality [31]. Concurrently, the theoretical framework of short-term repair in procrastination posits that procrastination is emblematic of self-regulation failure. In this scenario, immediate negative emotions outweigh the eventual long-term benefits. Mindfulness has been found to bolster emotional regulation and self-control capacities, effectively curbing procrastinatory tendencies [9,32]. At bedtime, procrastination can be a manifestation of procrastinatory behavior, wherein shared psychological mechanisms are evident, and given the demonstrated correlation between general procrastination and similar mechanisms [31]. Mindfulness could impact sleep quality by reducing bedtime procrastination. Thus, the hypothesis H3 emerges: Sleep delay functions as a mediating factor between Mindfulness and innovative sleep quality.

### 1.4 Chain-mediating effects of social interaction anxiety and bedtime procrastination

There is a significant correlation observed between Social Interaction Anxiety and sleep delay. Social Interaction Anxiety often triggers increased negative emotions in individuals. According to the short-term repair theory, heightened negative emotions and distress before sleep lead individuals to resort to bedtime procrastination as a coping mechanism to regulate their emotional state [33]. Consequently, a greater magnitude of negative emotions corresponds to a heightened tendency toward sleep delay. Conversely, the negative feelings associated with sleep-related cues may lead to sleep-related errors, culminating in an adverse experiential perception of sleep-related pressures [34]. It, in turn, prompts individuals to harbor an aversion toward sleep and subsequently adopt procrastinatory behaviors [35]. Empirical investigations have further indicated a correlation between general procrastinatory tendencies and heightened Social Interaction Anxiety in response to negative emotional stimuli [36]. Building upon these observations, hypothesis H4 emerges: Social Interaction Anxiety and sleep delay may operate collaboratively as mediating variables between Mindfulness and sleep quality.

In summation, this study endeavors to construct a comprehensive chain mediation model, thereby delving into the intricate mechanism through which Mindfulness interfaces with the sleep quality of innovative enterprise employees. Furthermore, it seeks to elucidate the intermediary roles of Social Interaction Anxiety and sleep delay. Such insights can potentially guide creative enterprise managers in devising strategies for the preemptive management of sleep-related issues and formulating effective intervention programs.

## 2. Materials and methods

### 2.1 Participants

Using a convenience sampling approach, this study gathered data from 1648 employees across 51 innovative enterprises in Zhejiang, Jiangsu, Shanghai, and Guangdong provinces. Data collection was facilitated through an online questionnaire, ensuring accessibility for participants. Before the survey commencement, participants received standardized instructions emphasizing the importance of providing genuine responses while guaranteeing the confidentiality and anonymity of their answers. A total of 1623 completed questionnaires were obtained. After filtering out responses with exceptionally brief completion times or displaying regular patterns, 1482 valid questionnaires remained suitable for analysis, resulting in an effective response rate of 91.31%. Among this sample, 975 respondents identified as male, and 507 as female, with ages ranging from 21 to 53 years and a mean age of 30.02 ± 21.27. Data collection for this study occurred between 20 April 2022 and 25 June 2022.

#### Ethical statement

Informed consent was obtained from all participants before their involvement in the study. Participants were provided with detailed information regarding the purpose and procedures of the research, their rights, and the confidentiality and anonymity of their responses. All the participants were over the age of 18. All data collected were treated with confidentiality and stored securely. Only researchers directly involved in data analysis had access to the data, and all personal identifiers were removed to ensure anonymity. This study did not involve any risks to participants; their participation was voluntary. Participants were informed that they could withdraw from the study without consequences. The study results will be used for research purposes only and disseminated through scholarly publications and presentations. Further, the Institutional Review Board (IRB) of Zhejiang Shuren University has reviewed the research project titled "Impact of Mindfulness on Sleep Quality in Innovative Corporate Employees: A Chain Mediation of Social Interaction Anxiety and Bedtime Procrastination," and upon careful consideration of the project’s objectives, methods, and participant involvement, the IRB has determined that formal ethical approval is not required.

### 2.2 Measures

#### 2.2.1 Mindfulness scale

The Mindfulness Awareness Scale (MAAS), initially developed by Brown and Ryan in 2003, was further refined by Chen et al. in 2012. This scale is designed to evaluate levels of Mindfulness based on the concept of "current attention and awareness." It consists of a unidimensional structure comprising 15 items, each rated on a 6-point scale, ranging from 1 (usually) to 6 (never). A higher total score reflects a more prominent individual trait of Mindfulness. The confirmatory factor analysis yielded the following indices: Tucker-Lewis Index (TLI) = 0.904, Incremental Fit Index (IFI) = 0.918, Comparative Fit Index (CFI) = 0.917, Root Mean Square Residual (RMR) = 0.067, and Root Mean Square Error of Approximation (RMSEA) = 0.077. Additionally, Cronbach’s α coefficient was computed to be 0.856, indicating high internal consistency.

#### 2.2.2 Social interaction anxiety scale

In assessing Social Interaction Anxiety, the Song Hongyan scale, developed by Leary, Mattick, et al., was utilized in this research. This scale consists of seven items, categorized into two dimensions: real Social Interaction Anxiety and network Social Interaction Anxiety. Participants were instructed to rate each item on a 5-point Likert scale, ranging from 1 (completely inconsistent) to 5 (fully consistent). Higher cumulative scores denote increased levels of individual Social Interaction Anxiety. The internal consistency of the scale within this study, measured by Cronbach’s α coefficient, was calculated to be 0.749, indicating a satisfactory level of reliability.

#### 2.2.3 Sleep Delay Scale (Chinese version)

The Chinese version of the Delayed Sleep Scale, as developed and revised by [37], was employed in this investigation. This scale consists of nine items, rated on a 5-point scale ranging from 1 (never) to 5 (always). Notably, four items were reverse-scored, with higher scores indicating heightened sleep delay tendencies. The internal consistency coefficient for this scale in the present study was determined to be 0.843, indicating a reliable level of internal reliability.

#### 2.2.4 Pittsburgh sleep quality index quantitative table

The Chinese version of the Pittsburgh Sleep Quality Index, as revised by Zou et al. [8] was employed in this research. This scale consists of 19 items, enabling the assessment of seven components, including sleep efficiency. Each item and component are rated on a scale from 0 to 3. Higher scores indicate poorer sleep quality among participants. The internal consistency coefficient for this scale in the study was calculated to be 0.775, indicating a reliable level of internal reliability.

### 2.3 Statistical analysis

The data were analyzed using SPSS 26.0 statistical software. Pearson correlation was utilized to examine the associations among the main variables. Additionally, PROCESS V3.5 software, combined with the Bootstrap method, was employed to investigate the chain mediation effect. Statistical significance was set at P < 0.05.

## 3. Results

### 3.1 Test for common method deviation

Harman’s univariate test was utilized to assess the potential common method bias among the variables investigated in this study. The results revealed nine common factors, each with an eigenvalue exceeding one. However, the first common factor explained only 18.60% of the variance, which fell below the critical threshold of 40%. Thus, it can be concluded that no significant common method bias influences the outcomes of the study [38].

### 3.2 Descriptive statistics and correlation analysis of the variables

When exploring the current status of sleep quality among innovative employees, it was found that a score of 8 on the Pittsburgh Sleep Quality Index (PSQI) denoted poor sleep quality for 461 participants, comprising 31.1% of the sample. A chi-square analysis assessing the distribution of sleep quality by gender revealed no statistically significant difference (χ^2^ = 0.83, P = 0.341) [36].

Pearson correlation analysis revealed significant correlations. A negative correlation was found between Mindfulness and variables including Social Interaction Anxiety, sleep delay, and sleep quality. Conversely, Social Interaction Anxiety positively correlated with both sleep delay and sleep quality. Additionally, a positive correlation was observed between sleep delay and sleep quality. Detailed results can be found in Table 1.

**Table 1:** Descriptive statistics and correlation analyses (N = 1482).

Variable	M±SD	1	2	3	2	3	4
1. Gender	1.68±0.42	1					
2. Age	27.±8.47	-0.008	1				
3. Mindfulness	2.45±0.71	-0.125***	0.04	1			
4. Social Interaction Anxiety	2.53±0.74	0.014	0.03	-0.71***	1		
5. Bedtime Procrastination	3.21±0.69	0.021	-0.03	-0.37***	0.23***	1	
6. Sleep quality	5.51±2.52	0.063*	0.05	-0.35***	0.37***	0.32***	1

Notes: Sex is a dummy variable, 1 = male, 2 = female

* = P <0.5

* * = P <0.01

* * * = P <0.001.

### 3.3 Mediation effect test

Employing the non-parametric percentile Bootstrap method as proposed by Hayes and utilizing an SPSS macro program while controlling for sex and age, the current study explored the sequential mediation effect. Mindfulness was designated as the independent variable, while sleep quality served as the dependent variable. Social Interaction Anxiety and sleep delay were identified as the mediating variables in the analysis. Model 6 from the PROCESS tool was selected for the assessment of the chain mediation effect [32,39].

**Fig 1:**
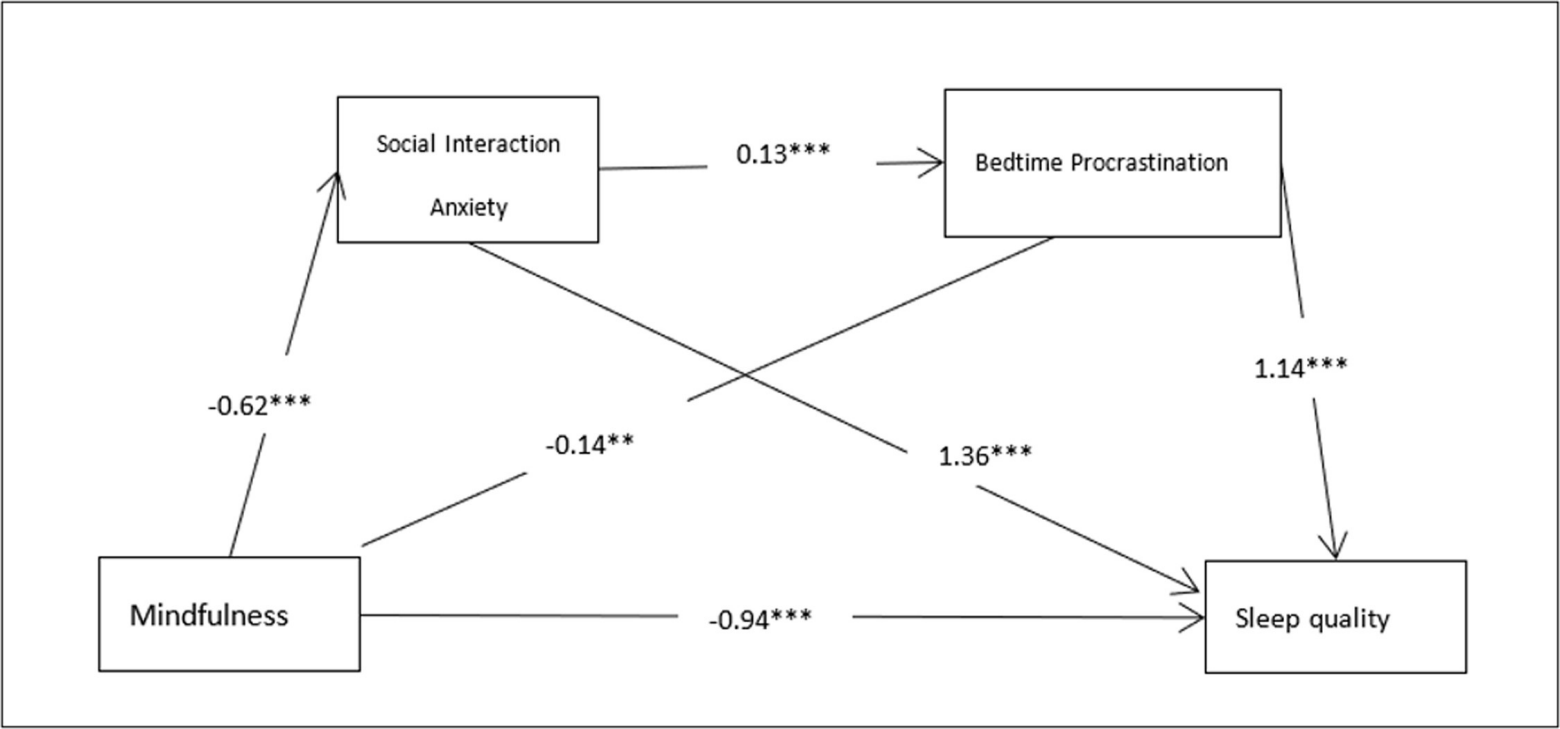
The conceptual framework depicting the chain mediation effect model involving Social Interaction Anxiety and bedtime procrastination.

The regression analysis revealed a statistically significant inverse association between Mindfulness and sleep quality (β = -0.35, P < 0.001). Subsequent mediation analysis uncovered several notable pathways: Mindfulness exhibited a significant negative impact on Social Interaction Anxiety (β = -0.71, P < 0.01), while also significantly predicting reduced sleep delay (β = -0.37, P < 0.01). Conversely, Social Interaction Anxiety positively correlated with sleep delay (β = 0.37, P < 0.001). When Mindfulness, Social Interaction Anxiety, and sleep delay were simultaneously introduced into the regression equation, noteworthy trends emerged: Mindfulness retained its negative association with sleep quality (β = -0.94, P < 0.001), whereas sleep delay demonstrated a positive prediction of sleep quality (β = 1.14, P < 0.001), both with confidence intervals excluding 0. Moreover, Social Interaction Anxiety positively predicted sleep quality (β = 1.36, P < 0.001), with its confidence interval similarly excluding 0. Additional details can be found in Tables 2 and 3. These findings underscore the significant sequential mediating influence of Social Interaction Anxiety and delayed sleep on the relationship between Mindfulness and sleep quality (see Fig 1).

**Table 2:** Regression analysis of the variable relationships in the mediation model.

Variables		Model fitting indicator			Effect value	
Outcome variable	Predictor variable	R	R2	F	β	t
Social Interaction Anxiety		0.4523	0.205	89.8714***		
	Gender				-0.03	-0.06
	Age				0.02	0.73
	Mindfulness				-0.62	-21.32***
Bedtime Procrastination		0.5128	0.263	136.3562***		
	Gender				-0.02	-0.26
	Age				-0.04	-1.63
	Mindfulness				-0.14	-3.41**
	Social Interaction Anxiety				0.13	4.16***
Sleep Quality		0.5423	0.2941	174.6725***		
	Gender				0.25	1.62
	Age				0.18	2.82**
	Mindfulness				-0.94	-6.71***
	Social Interaction Anxiety				1.36	17.65***
	Bedtime Procrastination				1.14	11.27***

**Table 3:** Bootstrap analysis of the significance test.

Mediation paths	Normalized path effect values	Effect rate	Bootstrap 95% CI	
			Lower	Upper
Mindfulness-Social interaction Anxiety-Sleep quality	0.092	10.26%	-0.29	-0.17
Mindfulness-Bedtime Procrastination-Sleep quality	0.075	9.14%	-0.34	-0.012
Mindfulness—Social Interaction Anxiety-Bedtime Procrastination-Sleep quality	0.107	15.64%	-0.26	-0.18
Total indirect	0.099	15.17%	-0.42	-0.16
Direct	0.533	84.83%	-1.13	-0.63
Total	0.632	100%	-1.89	-1.12

## 4. Discussion

Grounded in the cognitive model of insomnia, this study delves into the intricate interplay between Mindfulness and sleep quality among innovative employees. By introducing the mediating factors of Social Interaction Anxiety and sleep delay, it strives to elucidate the underlying mechanism through which trait mindfulness influences sleep quality. The study augments the cognitive model of insomnia and furnishes a theoretical foundation for addressing and mitigating sleep quality issues innovative employees face.

The outcomes from the structural equation model align with the research hypothesis H1, demonstrating that Mindfulness directly predicts the sleep quality of innovative enterprise employees. It underscores a positive relationship wherein higher levels of Mindfulness correspond to improved sleep quality. This enhancement can be attributed to Mindfulness’s ability to facilitate relaxation of the body and nerves through experiential awareness, attention control, and acceptance [40]. Importantly, given the prevalence of poor sleep quality among innovative employees stemming from stress, Mindfulness emerges as an effective strategy for stress reduction, mood elevation, and mitigating stress-induced cognitive intrusions, ultimately enhancing sleep quality [41]. Additionally, Mindfulness’s impact on insomnia can be attributed to its role in curbing primary arousal, alleviating the effects of stressors, and promoting improved sleep quality [42].

Further mediation effect analysis underscores the indirect influence of Mindfulness on sleep quality through the mediating effects of Social Interaction Anxiety and Bedtime Procrastination behavior, thereby validating the research hypotheses H2 and H3. This mediation underscores Mindfulness’s potential to alleviate stress and anxiety, enhancing and ameliorating Social Interaction Anxiety. This observation concurs with prior research [43,44] and is particularly relevant for innovative employees who often face heightened work-related stress and limited social interactions. Meanwhile, the frequent occurrence of sleep delay, particularly among the young population, finds its roots in the modern lifestyle characterized by technological engagement before bedtime. This behavior reflects an imbalance in self-control and emotional regulation abilities [44]. Remarkably, elevated Mindfulness levels are associated with improved self-control and emotional regulation, thereby reducing procrastination behavior and elevating sleep quality [45,46]. Consequently, integrating mindfulness training to intervene in Social Interaction Anxiety and sleep delay behaviors could offer a robust preventive measure for enhancing sleep health among innovative employees.

Furthermore, this study unveils the mediation role of Social Interaction Anxiety and sleep delay in linking Mindfulness with sleep quality among innovative corporate employees, supporting hypothesis H4. Notably, the reduction in social anxiety fostered by Mindfulness diminishes the likelihood of sleep procrastination, ultimately enhancing sleep quality. This insight aligns with the cognitive maintenance model of insomnia, which posits that counterproductive protective behaviors driven by negative cognitions, such as imagery control and anticipatory imagery of task processes, can exacerbate sleep delay [47–49]. Mindfulness training counters this pattern by nurturing perceptual and regulatory capacities [50]. Consequently, promoting mindfulness meditation training is a prospective strategy for addressing sleep quality concerns among innovative employees. It is essential, however, to recognize that while improved sleep quality is an important outcome, it is contingent upon the amelioration of Social Interaction Anxiety and Bedtime Procrastination behavior for effective change to occur [51].

## 5. Conclusions

This study explores the intricate connection between Mindfulness and sleep quality among creative firm personnel. After careful research, several significant findings have been revealed, providing insight into the complex processes at work. This study highlights a clear negative relationship between Mindfulness and sleep quality, showing that higher levels of Mindfulness are linked to better sleep among creative professionals. It shows that Mindfulness is crucial in predicting and maybe improving sleep problems in this group. We have explained how Social Interaction Anxiety acts as a mediator between Mindfulness and sleep quality. This study reveals how Social Interaction Anxiety, a prevalent psychological condition in organizational environments, mediates the relationship between Mindfulness practices and sleep quality. Our research shows that increased levels of Social Interaction Anxiety are linked to worse sleep quality. It highlights the importance of addressing this psychological factor in therapies designed to improve sleep among creative employees.

Furthermore, our research recognizes sleep latency as an additional important factor in the connection between Mindfulness and sleep quality. We investigate how postponing bedtime can worsen sleep issues, regardless of individuals’ mindfulness levels. It highlights the significance of addressing bedtime procrastination patterns while promoting Mindfulness practices to enhance sleep quality among creative people. Our work has verified the presence of a chain mediation process. Mindfulness affects sleep quality by going through Social Interaction Anxiety and sleep delay. This intricate relationship highlights how psychological elements and behavioral habits collectively impact the sleep outcomes of creative employees.

Our study provides a thorough contribution to understanding how Mindfulness influences sleep quality in modern work settings. We offer useful insights for firms aiming to promote healthier work environments and improve employee well-being by explaining how Mindfulness, Social Interaction Anxiety, and bedtime procrastination pathways combine to impact sleep results. Our research provides practical insights for creating specific interventions that target individual psychological aspects and organizational strategies to enhance the sleep quality of creative people.

## 6. Limitations

While offering valuable insights, the current study exhibits certain limitations that could be addressed for future improvement. Firstly, the sampling approach was confined to economically developed regions, including Zhejiang, Jiangsu, Shanghai, and Guangdong provinces, thereby restricting the study’s generalizability to a broader national context. Expanding the sample to encompass a more diverse geographic representation across China could bolster the applicability and generalizability of the study’s conclusions. Comprehensive empirical research on a larger scale would facilitate subsequent meta-analyses, further solidifying the findings.

Secondly, the study’s utilization of cross-sectional data inherently entails the presence of correlations among variables but precludes the establishment of causal relationships. Future research endeavors could leverage longitudinal tracking methods or experimental interventions to elucidate the causal dynamics between the variables. This approach would shed light on the temporal sequences and potential causal pathways, thereby bolstering the depth and breadth of the study’s conclusions.

Lastly, while the study thoughtfully delved into the mechanisms underlying the influence of Mindfulness on the sleep quality of innovative corporate employees, specifically exploring the roles of Social Interaction Anxiety and Bedtime Procrastination, other potential mediation or regulatory mechanisms could exist. Future research could explore and uncover additional mediating or moderating factors. Such investigations would refine the study’s explanatory framework and contribute to a more holistic comprehension of the relationship between Mindfulness and sleep quality in the context of innovative employees.

## Supporting information

S1 File Data is included in the Supporting information file. (PDF)
