# Supplementary material for: Impact of Mindfulness on sleep quality in innovative corporate employees: A chain mediation of Social Interaction Anxiety and bedtime procrastination
Source: PLoS One. 2024 May 22;19(5):e0302881. doi: 10.1371/journal.pone.0302881 (PMC11111030; doi:10.1371/journal.pone.0302881)
Supplement: S1 File — (PDF) [file pone.0302881.s001.pdf]

No	PSQ1_1	PSQ1_2	PSQ1_3	PSQ1_4	PSQ1_5	PSQ1_6	PSQ1_7	PSQ1_8	PSQ1_9
1	4	4	3	5	3	3	4	3	5
2	4	4	4	3	4	3	4	4	3
3	4	5	3	4	5	4	5	3	4
4	3	4	4	5	4	5	4	4	5
5	4	4	4	4	4	3	4	4	4
6	4	5	4	4	5	3	5	4	4
7	4	3	3	3	4	5	3	3	3
8	3	4	5	3	4	4	4	5	3
9	3	5	4	3	4	4	5	4	3
10	4	5	4	4	5	4	5	4	4
11	4	3	4	5	4	4	3	4	5
12	4	4	4	4	4	4	4	4	4
13	5	3	3	3	4	4	3	3	3
14	5	4	4	5	4	5	4	4	5
15	3	2	1	3	2	3	2	1	3
16	4	4	5	4	4	5	4	5	4
17	1	2	3	1	2	2	2	3	1
18	4	4	5	4	4	4	4	5	4
19	4	4	4	4	5	4	4	4	4
20	4	4	3	4	4	3	4	3	4
21	4	4	4	5	4	4	4	4	5
22	4	4	3	3	4	4	4	3	3
23	2	1	2	1	2	2	1	2	1
24	3	4	3	3	3	3	4	3	3
25	4	5	4	4	4	4	5	4	4
26	2	1	1	2	1	1	1	1	2
27	4	4	4	4	4	4	4	4	4
28	5	4	4	5	3	5	4	4	5
29	1	3	1	2	2	1	3	1	2
30	2	2	3	2	2	1	2	3	2
31	2	2	1	1	1	1	2	1	1
32	4	5	4	4	3	4	5	4	4
33	5	3	4	4	3	4	3	4	4
34	5	4	5	5	3	4	4	5	5
35	4	4	3	3	5	4	4	3	3
36	4	4	4	5	4	5	4	4	5
37	5	3	4	3	4	5	3	4	3
38	4	4	3	4	5	4	4	3	4
39	3	4	3	4	4	4	4	3	4
40	4	4	4	4	4	3	4	4	4
41	4	5	5	3	3	3	5	5	3
42	5	5	4	4	3	5	5	4	4
43	4	3	3	4	3	4	3	3	4
44	4	3	4	4	3	4	3	4	4
45	4	4	3	3	4	5	4	3	3
46	2	1	2	1	2	1	1	2	1

47	3	5	3	4	3	4	5	3	4
48	1	2	3	1	2	2	2	3	1
49	4	4	4	4	4	4	4	4	4
50	3	5	5	5	5	3	5	5	5
51	4	4	5	3	4	3	4	5	3
52	3	5	4	5	3	5	5	4	5
53	3	4	3	4	4	4	4	3	4
54	2	2	3	1	2	3	2	3	1
55	5	3	5	5	3	5	3	5	5
56	3	4	4	3	5	3	4	4	3
57	2	2	2	2	3	2	2	2	2
58	4	4	3	4	5	3	4	3	4
59	4	3	5	3	3	3	3	5	3
60	3	5	4	4	5	4	5	4	4
61	5	5	3	4	4	4	5	3	4
62	4	4	5	4	5	4	4	5	4
63	4	4	5	5	3	3	4	5	5
64	5	4	5	4	5	4	4	5	4
65	5	4	5	4	4	4	4	5	4
66	4	4	4	3	5	4	4	4	3
67	4	3	4	4	4	5	3	4	4
68	4	3	4	4	4	4	3	4	4
69	1	2	1	2	1	1	2	1	2
70	2	1	2	2	3	2	1	2	2
71	3	3	5	5	4	4	3	5	5
72	4	3	4	4	4	5	3	4	4
73	4	3	5	3	4	4	3	5	3
74	4	4	3	4	4	4	4	3	4
75	3	3	5	4	3	3	3	5	4
76	3	3	4	5	4	4	3	4	5
77	5	4	5	4	5	4	4	5	4
78	5	3	4	4	4	3	3	4	4
79	4	4	3	5	4	4	4	3	5
80	4	5	5	4	5	4	5	5	4
81	4	3	5	3	3	5	3	5	3
82	4	5	3	4	3	4	5	3	4
83	5	4	5	3	5	3	4	5	3
84	4	4	5	4	4	5	4	5	4
85	4	4	5	5	3	5	4	5	5
86	4	4	4	3	3	4	4	4	3
87	1	2	3	2	2	1	2	3	2
88	1	3	3	2	2	2	3	3	2
89	3	4	4	4	4	4	4	4	4
90	5	5	5	5	4	3	5	5	5
91	3	3	3	4	4	4	3	3	4
92	4	4	4	5	3	5	4	4	5
93	1	2	1	2	2	2	2	1	2

94	5	3	5	5	4	5	3	5	5
95	3	1	3	2	2	1	1	3	2
96	3	1	2	1	3	3	1	2	1
97	4	4	4	3	5	5	4	4	3
98	5	4	5	4	4	4	4	5	4
99	4	5	4	4	5	5	5	4	4
100	4	3	4	3	4	4	3	4	3
101	3	3	2	2	2	2	3	2	2
102	4	5	3	3	5	4	5	3	3
103	4	4	3	5	4	5	4	3	5
104	2	1	2	1	1	2	1	2	1
105	1	1	2	1	1	2	1	2	1
106	4	5	3	3	4	3	5	3	3
107	4	5	4	4	4	4	5	4	4
108	2	2	2	2	2	2	2	2	2
109	4	4	3	4	3	3	4	3	4
110	2	3	3	3	1	2	3	3	3
111	4	4	4	4	5	4	4	4	4
112	4	4	4	4	4	3	4	4	4
113	4	5	5	4	3	4	5	5	4
114	3	4	5	5	5	3	4	5	5
115	4	3	3	3	4	5	3	3	3
116	1	3	2	3	2	2	3	2	3
117	3	4	4	4	4	5	4	4	4
118	3	5	5	4	3	5	5	5	4
119	3	4	5	3	3	3	4	5	3
120	3	5	4	5	3	3	5	4	5
121	4	4	3	5	4	4	4	3	5
122	3	4	3	5	5	3	4	3	5
123	4	5	4	3	3	4	5	4	3
124	4	3	3	3	3	4	3	3	3
125	1	2	3	1	1	2	2	3	1
126	2	2	2	1	2	3	2	2	1
127	5	4	5	3	5	3	4	5	3
128	3	5	4	5	4	4	5	4	5
129	4	5	3	3	3	4	5	3	3
130	4	3	4	3	4	3	3	4	3
131	4	4	3	3	4	3	4	3	3
132	4	3	4	4	4	3	3	4	4
133	5	4	4	3	3	4	4	4	3
134	5	5	3	5	5	5	5	3	5
135	4	4	3	3	5	4	4	3	3
136	3	4	5	4	5	3	4	5	4
137	4	4	4	4	5	5	4	4	4
138	5	4	4	3	3	4	4	4	3
139	3	4	5	3	4	4	4	5	3
140	5	3	4	5	3	5	3	4	5

141	2	3	1	2	3	1	3	1	2
142	4	4	4	3	4	4	4	4	3
143	4	3	4	4	5	3	3	4	4
144	4	5	4	4	5	5	5	4	4
145	4	3	5	3	5	3	3	5	3
146	5	4	4	3	4	5	4	4	3
147	3	5	3	4	3	4	5	3	4
148	5	4	3	4	4	4	4	3	4
149	3	5	4	4	5	4	5	4	4
150	4	5	3	3	4	4	5	3	3
151	4	3	5	3	4	5	3	5	3
152	3	4	4	4	4	4	4	4	4
153	5	5	5	3	4	3	5	5	3
154	1	2	3	3	1	1	2	3	3
155	1	3	1	1	2	1	3	1	1
156	2	2	2	1	2	2	2	2	1
157	1	2	2	2	2	2	2	2	2
158	4	5	5	5	4	4	5	5	5
159	3	4	5	3	4	4	4	5	3
160	3	5	4	5	4	4	5	4	5
161	4	3	3	3	4	3	3	3	3
162	2	2	1	3	2	2	2	1	3
163	3	5	5	3	3	5	5	5	3
164	4	3	5	4	3	4	3	5	4
165	4	4	4	3	4	3	4	4	3
166	5	5	4	4	4	3	5	4	4
167	3	5	4	4	5	4	5	4	4
168	3	5	4	4	4	5	5	4	4
169	3	3	5	3	4	4	3	5	3
170	5	4	4	3	5	5	4	4	3
171	4	4	5	5	4	5	4	5	5
172	4	5	4	4	4	4	5	4	4
173	5	5	4	4	5	4	5	4	4
174	4	3	5	3	5	5	3	5	3
175	3	4	3	3	4	4	4	3	3
176	4	4	3	4	4	5	4	3	4
177	4	3	3	5	5	4	3	3	5
178	5	3	3	3	4	4	3	3	3
179	4	5	3	5	4	3	5	3	5
180	1	3	2	3	3	2	3	2	3
181	4	3	4	4	4	4	3	4	4
182	5	5	4	4	3	4	5	4	4
183	5	4	4	4	5	5	4	4	4
184	2	2	1	2	2	2	2	1	2
185	3	4	4	4	5	5	4	4	4
186	2	1	2	2	2	3	1	2	2
187	4	4	3	3	4	4	4	3	3

188	4	5	3	4	5	4	5	3	4
189	5	5	3	5	3	3	5	3	5
190	4	4	3	5	4	5	4	3	5
191	4	4	3	4	4	4	4	3	4
192	4	3	4	5	4	4	3	4	5
193	5	4	5	4	3	3	4	5	4
194	1	1	2	3	2	2	1	2	3
195	5	3	3	5	3	4	3	3	5
196	4	5	3	3	4	5	5	3	3
197	4	4	5	5	5	4	4	5	5
198	5	4	4	5	5	3	4	4	5
199	2	1	1	1	1	2	1	1	1
200	4	4	3	4	4	3	4	3	4
201	5	4	4	4	4	4	4	4	4
202	3	4	4	4	4	3	4	4	4
203	3	4	4	4	4	4	4	4	4
204	5	4	5	3	5	4	4	5	3
205	5	5	5	5	3	3	5	5	5
206	3	3	3	2	2	2	3	3	2
207	4	5	4	5	4	4	5	4	5
208	4	3	3	5	5	4	3	3	5
209	4	5	4	5	3	4	5	4	5
210	4	4	5	3	4	4	4	5	3
211	5	4	4	5	5	4	4	4	5
212	4	4	4	5	4	5	4	4	5
213	1	2	1	2	2	2	2	1	2
214	4	5	4	4	4	4	5	4	4
215	3	4	3	4	4	4	4	3	4
216	3	4	4	5	5	5	4	4	5
217	3	5	5	4	4	3	5	5	4
218	3	5	3	5	4	4	5	3	5
219	4	5	3	4	3	4	5	3	4
220	5	4	5	4	3	4	4	5	4
221	4	3	4	5	4	4	3	4	5
222	3	4	4	4	5	4	4	4	4
223	3	5	4	5	3	5	5	4	5
224	4	4	4	5	3	5	4	4	5
225	3	4	5	4	4	3	4	5	4
226	4	3	5	4	5	3	3	5	4
227	4	3	3	3	3	4	3	3	3
228	5	4	4	4	4	5	4	4	4
229	3	3	3	2	2	3	3	3	2
230	4	4	5	4	4	5	4	5	4
231	3	5	4	4	4	3	5	4	4
232	3	2	2	2	2	1	2	2	2
233	4	3	3	5	4	4	3	3	5
234	4	4	4	3	5	4	4	4	3

235	3	2	3	2	2	2	2	3	2
236	5	5	3	4	4	3	5	3	4
237	5	5	4	3	3	5	5	4	3
238	4	5	4	4	4	4	5	4	4
239	4	3	3	3	4	4	3	3	3
240	4	4	4	4	3	4	4	4	4
241	4	3	4	4	4	5	3	4	4
242	3	4	3	5	4	3	4	3	5
243	4	5	4	5	4	4	5	4	5
244	3	5	3	3	4	3	5	3	3
245	3	4	5	3	5	3	4	5	3
246	5	4	3	3	4	5	4	3	3
247	2	2	2	1	2	1	2	2	1
248	4	5	5	4	3	4	5	5	4
249	3	4	3	3	5	4	4	3	3
250	5	5	4	4	3	3	5	4	4
251	5	4	5	4	3	4	4	5	4
252	4	4	5	4	4	4	4	5	4
253	4	4	5	4	5	3	4	5	4
254	5	4	3	4	5	5	4	3	4
255	3	4	5	4	4	3	4	5	4
256	3	3	5	4	4	4	3	5	4
257	4	5	4	5	3	3	5	4	5
258	4	3	4	4	4	4	3	4	4
259	5	5	4	4	4	3	5	4	4
260	5	4	4	5	4	5	4	4	5
261	3	2	2	3	2	3	2	2	3
262	4	4	3	4	4	4	4	3	4
263	3	4	3	3	4	4	4	3	3
264	4	4	3	3	4	3	4	3	3
265	4	4	4	3	4	3	4	4	3
266	5	3	4	4	4	5	3	4	4
267	3	4	5	4	4	3	4	5	4
268	4	4	5	4	3	4	4	5	4
269	5	4	5	3	4	5	4	5	3
270	4	4	4	4	5	3	4	4	4
271	4	3	3	3	4	4	3	3	3
272	5	4	3	3	4	4	4	3	3
273	1	3	3	3	1	2	3	3	3
274	3	4	5	5	4	4	4	5	5
275	4	4	3	3	3	4	4	3	3
276	4	4	4	4	3	3	4	4	4
277	5	4	4	5	3	3	4	4	5
278	4	3	4	3	3	4	3	4	3
279	3	4	3	4	3	4	4	3	4
280	4	5	4	5	3	4	5	4	5
281	5	5	5	5	5	4	5	5	5

282	5	3	4	3	4	4	3	4	3
283	3	5	3	3	4	3	5	3	3
284	1	1	1	2	1	2	1	1	2
285	3	5	5	4	5	4	5	5	4
286	4	4	4	5	4	5	4	4	5
287	4	3	4	3	5	4	3	4	3
288	2	3	3	3	2	1	3	3	3
289	5	5	5	4	5	3	5	5	4
290	4	5	4	3	3	4	5	4	3
291	2	2	2	1	1	2	2	2	1
292	4	5	4	4	5	4	5	4	4
293	4	4	4	4	5	5	4	4	4
294	4	5	4	4	5	4	5	4	4
295	3	5	3	3	3	3	5	3	3
296	5	3	4	4	5	3	3	4	4
297	5	4	4	3	4	5	4	4	3
298	3	2	2	2	1	2	2	2	2
299	2	2	2	1	2	2	2	2	1
300	3	4	4	3	5	5	4	4	3
301	5	5	4	5	4	3	5	4	5
302	5	3	3	4	4	4	3	3	4
303	3	2	2	2	1	3	2	2	2
304	3	4	3	4	3	4	4	3	4
305	3	5	3	4	4	3	5	3	4
306	4	3	5	3	4	3	3	5	3
307	5	4	5	4	4	5	4	5	4
308	3	3	4	5	4	4	3	4	5
309	4	3	4	5	4	4	3	4	5
310	5	5	3	5	4	3	5	3	5
311	4	4	5	5	5	4	4	5	5
312	5	3	3	5	3	5	3	3	5
313	4	4	3	3	3	4	4	3	3
314	4	4	4	5	5	3	4	4	5
315	4	4	4	4	4	5	4	4	4
316	4	3	3	3	3	5	3	3	3
317	4	5	5	4	4	4	5	5	4
318	5	3	3	3	4	5	3	3	3
319	3	4	4	4	4	4	4	4	4
320	1	1	2	1	2	1	1	2	1
321	5	4	5	4	4	3	4	5	4
322	1	2	2	2	2	3	2	2	2
323	1	2	2	2	2	3	2	2	2
324	3	4	4	4	3	5	4	4	4
325	4	4	5	4	3	4	4	5	4
326	4	4	4	3	4	3	4	4	3
327	4	3	4	5	3	5	3	4	5
328	5	4	4	5	5	4	4	4	5

329	5	5	4	3	3	4	5	4	3
330	2	3	2	2	2	2	3	2	2
331	5	4	3	3	4	4	4	3	3
332	3	4	5	4	4	3	4	5	4
333	5	4	4	4	4	4	4	4	4
334	2	1	2	3	2	1	1	2	3
335	3	4	3	4	3	3	4	3	4
336	4	5	4	4	3	5	5	4	4
337	3	3	4	4	4	3	3	4	4
338	5	4	5	4	3	5	4	5	4
339	5	5	4	4	3	5	5	4	4
340	4	5	3	5	3	3	5	3	5
341	3	1	2	2	1	2	1	2	2
342	1	1	3	3	2	1	1	3	3
343	3	2	2	2	2	3	2	2	2
344	4	4	3	3	3	3	4	3	3
345	4	3	5	5	5	5	3	5	5
346	3	4	5	5	4	4	4	5	5
347	3	5	3	4	5	4	5	3	4
348	2	1	1	1	1	2	1	1	1
349	1	1	3	2	1	2	1	3	2
350	3	4	4	3	3	4	4	4	3
351	4	4	4	4	5	4	4	4	4
352	4	4	4	5	4	4	4	4	5
353	5	5	4	4	4	4	5	4	4
354	5	3	4	3	4	5	3	4	3
355	1	2	1	1	2	1	2	1	1
356	2	2	2	3	1	2	2	2	3
357	4	5	3	4	4	5	5	3	4
358	3	4	3	4	3	3	4	3	4
359	3	2	2	2	1	3	2	2	2
360	4	4	4	4	5	4	4	4	4
361	5	4	3	3	4	4	4	3	3
362	2	1	2	2	1	3	1	2	2
363	3	3	5	5	3	4	3	5	5
364	5	3	4	4	5	5	3	4	4
365	3	4	5	4	3	5	4	5	4
366	4	4	4	4	5	3	4	4	4
367	5	5	3	4	4	3	5	3	4
368	5	4	4	3	4	5	4	4	3
369	3	4	3	4	5	4	4	3	4
370	4	4	4	5	4	4	4	4	5
371	5	3	3	4	4	5	3	3	4
372	2	2	1	2	2	2	2	1	2
373	5	4	4	3	3	5	4	4	3
374	4	5	3	4	3	3	5	3	4
375	4	4	3	4	4	4	4	3	4

376	3	5	5	3	4	4	5	5	3
377	3	4	3	4	5	3	4	3	4
378	4	5	5	5	4	3	5	5	5
379	4	4	5	3	4	4	4	5	3
380	4	4	5	3	4	5	4	5	3
381	4	3	3	4	3	5	3	3	4
382	5	3	3	5	4	3	3	3	5
383	5	4	3	3	4	3	4	3	3
384	1	1	1	1	2	2	1	1	1
385	2	2	2	2	1	2	2	2	2
386	4	5	5	3	5	5	5	5	3
387	3	4	4	3	3	4	4	4	3
388	4	4	3	5	5	4	4	3	5
389	4	4	3	4	4	5	4	3	4
390	3	4	3	5	4	4	4	3	5
391	4	4	4	4	4	4	4	4	4
392	4	4	4	3	3	4	4	4	3
393	5	4	5	4	3	5	4	5	4
394	5	3	4	3	4	4	3	4	3
395	5	3	4	3	5	5	3	4	3
396	5	4	3	5	5	5	4	3	5
397	4	4	4	3	3	3	4	4	3
398	4	5	4	3	3	5	5	4	3
399	4	5	4	5	3	4	5	4	5
400	2	3	1	2	3	3	3	1	2
401	4	4	4	4	4	3	4	4	4
402	4	4	3	3	4	3	4	3	3
403	5	4	5	4	3	5	4	5	4
404	4	4	4	5	4	3	4	4	5
405	5	3	3	3	5	3	3	3	3
406	1	3	2	1	2	3	3	2	1
407	5	4	4	3	3	5	4	4	3
408	3	5	4	5	5	5	5	4	5
409	5	3	3	3	4	4	3	3	3
410	3	4	4	5	5	3	4	4	5
411	5	4	3	4	3	5	4	3	4
412	1	1	2	2	2	2	1	2	2
413	5	3	4	3	5	4	3	4	3
414	4	3	4	4	4	3	3	4	4
415	2	2	2	3	2	2	2	2	3
416	3	3	4	4	5	5	3	4	4
417	4	4	3	4	4	4	4	3	4
418	2	2	3	2	1	2	2	3	2
419	1	1	3	2	1	1	1	3	2
420	4	3	4	3	3	3	3	4	3
421	4	5	5	4	3	4	5	5	4
422	3	3	4	4	5	4	3	4	4

423	3	5	5	4	3	4	5	5	4
424	3	5	4	4	5	3	5	4	4
425	4	4	3	5	5	3	4	3	5
426	3	4	4	4	4	3	4	4	4
427	5	4	4	4	4	4	4	4	4
428	3	4	4	4	4	4	4	4	4
429	4	5	4	4	5	4	5	4	4
430	4	3	4	4	4	5	3	4	4
431	3	5	3	3	4	3	5	3	3
432	3	5	5	4	3	4	5	5	4
433	4	5	5	4	3	3	5	5	4
434	4	3	4	3	3	5	3	4	3
435	4	3	3	3	4	5	3	3	3
436	5	5	5	3	4	4	5	5	3
437	2	2	3	1	2	3	2	3	1
438	4	4	4	4	3	3	4	4	4
439	5	4	5	4	3	4	4	5	4
440	3	4	3	4	5	4	4	3	4
441	5	4	5	3	3	5	4	5	3
442	4	4	4	5	5	4	4	4	5
443	4	4	3	3	3	5	4	3	3
444	3	3	4	5	4	5	3	4	5
445	5	5	4	4	4	4	5	4	4
446	4	4	4	4	4	4	4	4	4
447	4	4	5	3	3	4	4	5	3
448	2	2	1	2	2	2	2	1	2
449	4	5	4	4	4	4	5	4	4
450	4	5	4	4	3	4	5	4	4
451	5	4	5	3	5	4	4	5	3
452	2	1	1	2	2	2	1	1	2
453	5	4	5	5	5	4	4	5	5
454	5	3	5	3	3	4	3	5	3
455	3	2	3	3	2	2	2	3	3
456	2	2	3	2	2	2	2	3	2
457	2	1	2	2	3	3	1	2	2
458	4	5	3	4	4	4	5	3	4
459	5	5	5	4	4	4	5	5	4
460	4	4	3	4	4	4	4	3	4
461	4	3	3	5	4	4	3	3	5
462	4	4	3	5	3	3	4	3	5
463	4	4	4	3	4	3	4	4	3
464	4	5	3	4	5	4	5	3	4
465	3	4	4	5	4	5	4	4	5
466	4	4	4	4	4	3	4	4	4
467	4	5	4	4	5	3	5	4	4
468	4	3	3	3	4	5	3	3	3
469	3	4	5	3	4	4	4	5	3

470	3	5	4	3	4	4	5	4	3
471	4	5	4	4	5	4	5	4	4
472	4	3	4	5	4	4	3	4	5
473	4	4	4	4	4	4	4	4	4
474	5	3	3	3	4	4	3	3	3
475	5	4	4	5	4	5	4	4	5
476	3	2	1	3	2	3	2	1	3
477	4	4	5	4	4	5	4	5	4
478	1	2	3	1	2	2	2	3	1
479	4	4	5	4	4	4	4	5	4
480	4	4	4	4	5	4	4	4	4
481	4	4	3	4	4	3	4	3	4
482	4	4	4	5	4	4	4	4	5
483	4	4	3	3	4	4	4	3	3
484	2	1	2	1	2	2	1	2	1
485	3	4	3	3	3	3	4	3	3
486	4	5	4	4	4	4	5	4	4
487	2	1	1	2	1	1	1	1	2
488	4	4	4	4	4	4	4	4	4
489	5	4	4	5	3	5	4	4	5
490	1	3	1	2	2	1	3	1	2
491	2	2	3	2	2	1	2	3	2
492	2	2	1	1	1	1	2	1	1
493	4	5	4	4	3	4	5	4	4
494	5	3	4	4	3	4	3	4	4
495	5	4	5	5	3	4	4	5	5
496	4	4	3	3	5	4	4	3	3
497	4	4	4	5	4	5	4	4	5
498	5	3	4	3	4	5	3	4	3
499	4	4	3	4	5	4	4	3	4
500	3	4	3	4	4	4	4	3	4
501	4	4	4	4	4	3	4	4	4
502	4	5	5	3	3	3	5	5	3
503	5	5	4	4	3	5	5	4	4
504	4	3	3	4	3	4	3	3	4
505	4	3	4	4	3	4	3	4	4
506	4	4	3	3	4	5	4	3	3
507	2	1	2	1	2	1	1	2	1
508	3	5	3	4	3	4	5	3	4
509	1	2	3	1	2	2	2	3	1
510	4	4	4	4	4	4	4	4	4
511	3	5	5	5	5	3	5	5	5
512	4	4	5	3	4	3	4	5	3
513	3	5	4	5	3	5	5	4	5
514	3	4	3	4	4	4	4	3	4
515	2	2	3	1	2	3	2	3	1
516	5	3	5	5	3	5	3	5	5

517	3	4	4	3	5	3	4	4	3
518	2	2	2	2	3	2	2	2	2
519	4	4	3	4	5	3	4	3	4
520	4	3	5	3	3	3	3	5	3
521	3	5	4	4	5	4	5	4	4
522	5	5	3	4	4	4	5	3	4
523	4	4	5	4	5	4	4	5	4
524	4	4	5	5	3	3	4	5	5
525	5	4	5	4	5	4	4	5	4
526	5	4	5	4	4	4	4	5	4
527	4	4	4	3	5	4	4	4	3
528	4	3	4	4	4	5	3	4	4
529	4	3	4	4	4	4	3	4	4
530	1	2	1	2	1	1	2	1	2
531	2	1	2	2	3	2	1	2	2
532	3	3	5	5	4	4	3	5	5
533	4	3	4	4	4	5	3	4	4
534	4	3	5	3	4	4	3	5	3
535	4	4	3	4	4	4	4	3	4
536	3	3	5	4	3	3	3	5	4
537	3	3	4	5	4	4	3	4	5
538	5	4	5	4	5	4	4	5	4
539	5	3	4	4	4	3	3	4	4
540	4	4	3	5	4	4	4	3	5
541	4	5	5	4	5	4	5	5	4
542	4	3	5	3	3	5	3	5	3
543	4	5	3	4	3	4	5	3	4
544	5	4	5	3	5	3	4	5	3
545	4	4	5	4	4	5	4	5	4
546	4	4	5	5	3	5	4	5	5
547	4	4	4	3	3	4	4	4	3
548	1	2	3	2	2	1	2	3	2
549	1	3	3	2	2	2	3	3	2
550	3	4	4	4	4	4	4	4	4
551	5	5	5	5	4	3	5	5	5
552	3	3	3	4	4	4	3	3	4
553	4	4	4	5	3	5	4	4	5
554	1	2	1	2	2	2	2	1	2
555	5	3	5	5	4	5	3	5	5
556	3	1	3	2	2	1	1	3	2
557	3	1	2	1	3	3	1	2	1
558	4	4	4	3	5	5	4	4	3
559	5	4	5	4	4	4	4	5	4
560	4	5	4	4	5	5	5	4	4
561	4	3	4	3	4	4	3	4	3
562	3	3	2	2	2	2	3	2	2
563	4	5	3	3	5	4	5	3	3

564	4	4	3	5	4	5	4	3	5
565	2	1	2	1	1	2	1	2	1
566	1	1	2	1	1	2	1	2	1
567	4	5	3	3	4	3	5	3	3
568	4	5	4	4	4	4	5	4	4
569	2	2	2	2	2	2	2	2	2
570	4	4	3	4	3	3	4	3	4
571	2	3	3	3	1	2	3	3	3
572	4	4	4	4	5	4	4	4	4
573	4	4	4	4	4	3	4	4	4
574	4	5	5	4	3	4	5	5	4
575	3	4	5	5	5	3	4	5	5
576	4	3	3	3	4	5	3	3	3
577	1	3	2	3	2	2	3	2	3
578	3	4	4	4	4	5	4	4	4
579	3	5	5	4	3	5	5	5	4
580	3	4	5	3	3	3	4	5	3
581	3	5	4	5	3	3	5	4	5
582	4	4	3	5	4	4	4	3	5
583	3	4	3	5	5	3	4	3	5
584	4	5	4	3	3	4	5	4	3
585	4	3	3	3	3	4	3	3	3
586	1	2	3	1	1	2	2	3	1
587	2	2	2	1	2	3	2	2	1
588	5	4	5	3	5	3	4	5	3
589	3	5	4	5	4	4	5	4	5
590	4	5	3	3	3	4	5	3	3
591	4	3	4	3	4	3	3	4	3
592	4	4	3	3	4	3	4	3	3
593	4	3	4	4	4	3	3	4	4
594	5	4	4	3	3	4	4	4	3
595	5	5	3	5	5	5	5	3	5
596	4	4	3	3	5	4	4	3	3
597	3	4	5	4	5	3	4	5	4
598	4	4	4	4	5	5	4	4	4
599	5	4	4	3	3	4	4	4	3
600	3	4	5	3	4	4	4	5	3
601	5	3	4	5	3	5	3	4	5
602	2	3	1	2	3	1	3	1	2
603	4	4	4	3	4	4	4	4	3
604	4	3	4	4	5	3	3	4	4
605	4	5	4	4	5	5	5	4	4
606	4	3	5	3	5	3	3	5	3
607	5	4	4	3	4	5	4	4	3
608	3	5	3	4	3	4	5	3	4
609	5	4	3	4	4	4	4	3	4
610	3	5	4	4	5	4	5	4	4

611	4	5	3	3	4	4	5	3	3
612	4	3	5	3	4	5	3	5	3
613	3	4	4	4	4	4	4	4	4
614	5	5	5	3	4	3	5	5	3
615	1	2	3	3	1	1	2	3	3
616	1	3	1	1	2	1	3	1	1
617	2	2	2	1	2	2	2	2	1
618	1	2	2	2	2	2	2	2	2
619	4	5	5	5	4	4	5	5	5
620	3	4	5	3	4	4	4	5	3
621	3	5	4	5	4	4	5	4	5
622	4	3	3	3	4	3	3	3	3
623	2	2	1	3	2	2	2	1	3
624	3	5	5	3	3	5	5	5	3
625	4	3	5	4	3	4	3	5	4
626	4	4	4	3	4	3	4	4	3
627	5	5	4	4	4	3	5	4	4
628	3	5	4	4	5	4	5	4	4
629	3	5	4	4	4	5	5	4	4
630	3	3	5	3	4	4	3	5	3
631	5	4	4	3	5	5	4	4	3
632	4	4	5	5	4	5	4	5	5
633	4	5	4	4	4	4	5	4	4
634	5	5	4	4	5	4	5	4	4
635	4	3	5	3	5	5	3	5	3
636	3	4	3	3	4	4	4	3	3
637	4	4	3	4	4	5	4	3	4
638	4	3	3	5	5	4	3	3	5
639	5	3	3	3	4	4	3	3	3
640	4	5	3	5	4	3	5	3	5
641	1	3	2	3	3	2	3	2	3
642	4	3	4	4	4	4	3	4	4
643	5	5	4	4	3	4	5	4	4
644	5	4	4	4	5	5	4	4	4
645	2	2	1	2	2	2	2	1	2
646	3	4	4	4	5	5	4	4	4
647	2	1	2	2	2	3	1	2	2
648	4	4	3	3	4	4	4	3	3
649	4	5	3	4	5	4	5	3	4
650	5	5	3	5	3	3	5	3	5
651	4	4	3	5	4	5	4	3	5
652	4	4	3	4	4	4	4	3	4
653	4	3	4	5	4	4	3	4	5
654	5	4	5	4	3	3	4	5	4
655	1	1	2	3	2	2	1	2	3
656	5	3	3	5	3	4	3	3	5
657	4	5	3	3	4	5	5	3	3

658	4	4	5	5	5	4	4	5	5
659	5	4	4	5	5	3	4	4	5
660	2	1	1	1	1	2	1	1	1
661	4	4	3	4	4	3	4	3	4
662	5	4	4	4	4	4	4	4	4
663	3	4	4	4	4	3	4	4	4
664	3	4	4	4	4	4	4	4	4
665	5	4	5	3	5	4	4	5	3
666	5	5	5	5	3	3	5	5	5
667	3	3	3	2	2	2	3	3	2
668	4	5	4	5	4	4	5	4	5
669	4	3	3	5	5	4	3	3	5
670	4	5	4	5	3	4	5	4	5
671	4	4	5	3	4	4	4	5	3
672	5	4	4	5	5	4	4	4	5
673	4	4	4	5	4	5	4	4	5
674	1	2	1	2	2	2	2	1	2
675	4	5	4	4	4	4	5	4	4
676	3	4	3	4	4	4	4	3	4
677	3	4	4	5	5	5	4	4	5
678	3	5	5	4	4	3	5	5	4
679	3	5	3	5	4	4	5	3	5
680	4	5	3	4	3	4	5	3	4
681	5	4	5	4	3	4	4	5	4
682	4	3	4	5	4	4	3	4	5
683	3	4	4	4	5	4	4	4	4
684	3	5	4	5	3	5	5	4	5
685	4	4	4	5	3	5	4	4	5
686	3	4	5	4	4	3	4	5	4
687	4	3	5	4	5	3	3	5	4
688	4	3	3	3	3	4	3	3	3
689	5	4	4	4	4	5	4	4	4
690	3	3	3	2	2	3	3	3	2
691	4	4	5	4	4	5	4	5	4
692	3	5	4	4	4	3	5	4	4
693	3	2	2	2	2	1	2	2	2
694	4	3	3	5	4	4	3	3	5
695	4	4	4	3	5	4	4	4	3
696	3	2	3	2	2	2	2	3	2
697	5	5	3	4	4	3	5	3	4
698	5	5	4	3	3	5	5	4	3
699	4	5	4	4	4	4	5	4	4
700	4	3	3	3	4	4	3	3	3
701	4	4	4	4	3	4	4	4	4
702	4	3	4	4	4	5	3	4	4
703	3	4	3	5	4	3	4	3	5
704	4	5	4	5	4	4	5	4	5

705	3	5	3	3	4	3	5	3	3
706	3	4	5	3	5	3	4	5	3
707	5	4	3	3	4	5	4	3	3
708	2	2	2	1	2	1	2	2	1
709	4	5	5	4	3	4	5	5	4
710	3	4	3	3	5	4	4	3	3
711	5	5	4	4	3	3	5	4	4
712	5	4	5	4	3	4	4	5	4
713	4	4	5	4	4	4	4	5	4
714	4	4	5	4	5	3	4	5	4
715	5	4	3	4	5	5	4	3	4
716	3	4	5	4	4	3	4	5	4
717	3	3	5	4	4	4	3	5	4
718	4	5	4	5	3	3	5	4	5
719	4	3	4	4	4	4	3	4	4
720	5	5	4	4	4	3	5	4	4
721	5	4	4	5	4	5	4	4	5
722	3	2	2	3	2	3	2	2	3
723	4	4	3	4	4	4	4	3	4
724	3	4	3	3	4	4	4	3	3
725	4	4	3	3	4	3	4	3	3
726	4	4	4	3	4	3	4	4	3
727	5	3	4	4	4	5	3	4	4
728	3	4	5	4	4	3	4	5	4
729	4	4	5	4	3	4	4	5	4
730	5	4	5	3	4	5	4	5	3
731	4	4	4	4	5	3	4	4	4
732	4	3	3	3	4	4	3	3	3
733	5	4	3	3	4	4	4	3	3
734	1	3	3	3	1	2	3	3	3
735	3	4	5	5	4	4	4	5	5
736	4	4	3	3	3	4	4	3	3
737	4	4	4	4	3	3	4	4	4
738	5	4	4	5	3	3	4	4	5
739	4	3	4	3	3	4	3	4	3
740	3	4	3	4	3	4	4	3	4
741	4	5	4	5	3	4	5	4	5
742	5	5	5	5	5	4	5	5	5
743	5	3	4	3	4	4	3	4	3
744	3	5	3	3	4	3	5	3	3
745	1	1	1	2	1	2	1	1	2
746	3	5	5	4	5	4	5	5	4
747	4	4	4	5	4	5	4	4	5
748	4	3	4	3	5	4	3	4	3
749	2	3	3	3	2	1	3	3	3
750	5	5	5	4	5	3	5	5	4
751	4	5	4	3	3	4	5	4	3

752	2	2	2	1	1	2	2	2	1
753	4	5	4	4	5	4	5	4	4
754	4	4	4	4	5	5	4	4	4
755	4	5	4	4	5	4	5	4	4
756	3	5	3	3	3	3	5	3	3
757	5	3	4	4	5	3	3	4	4
758	5	4	4	3	4	5	4	4	3
759	3	2	2	2	1	2	2	2	2
760	2	2	2	1	2	2	2	2	1
761	3	4	4	3	5	5	4	4	3
762	5	5	4	5	4	3	5	4	5
763	5	3	3	4	4	4	3	3	4
764	3	2	2	2	1	3	2	2	2
765	3	4	3	4	3	4	4	3	4
766	3	5	3	4	4	3	5	3	4
767	4	3	5	3	4	3	3	5	3
768	5	4	5	4	4	5	4	5	4
769	3	3	4	5	4	4	3	4	5
770	4	3	4	5	4	4	3	4	5
771	5	5	3	5	4	3	5	3	5
772	4	4	5	5	5	4	4	5	5
773	5	3	3	5	3	5	3	3	5
774	4	4	3	3	3	4	4	3	3
775	4	4	4	5	5	3	4	4	5
776	4	4	4	4	4	5	4	4	4
777	4	3	3	3	3	5	3	3	3
778	4	5	5	4	4	4	5	5	4
779	5	3	3	3	4	5	3	3	3
780	3	4	4	4	4	4	4	4	4
781	1	1	2	1	2	1	1	2	1
782	5	4	5	4	4	3	4	5	4
783	1	2	2	2	2	3	2	2	2
784	1	2	2	2	2	3	2	2	2
785	3	4	4	4	3	5	4	4	4
786	4	4	5	4	3	4	4	5	4
787	4	4	4	3	4	3	4	4	3
788	4	3	4	5	3	5	3	4	5
789	5	4	4	5	5	4	4	4	5
790	5	5	4	3	3	4	5	4	3
791	2	3	2	2	2	2	3	2	2
792	5	4	3	3	4	4	4	3	3
793	3	4	5	4	4	3	4	5	4
794	5	4	4	4	4	4	4	4	4
795	2	1	2	3	2	1	1	2	3
796	3	4	3	4	3	3	4	3	4
797	4	5	4	4	3	5	5	4	4
798	3	3	4	4	4	3	3	4	4

799	5	4	5	4	3	5	4	5	4
800	5	5	4	4	3	5	5	4	4
801	4	5	3	5	3	3	5	3	5
802	3	1	2	2	1	2	1	2	2
803	1	1	3	3	2	1	1	3	3
804	3	2	2	2	2	3	2	2	2
805	4	4	3	3	3	3	4	3	3
806	4	3	5	5	5	5	3	5	5
807	3	4	5	5	4	4	4	5	5
808	3	5	3	4	5	4	5	3	4
809	2	1	1	1	1	2	1	1	1
810	1	1	3	2	1	2	1	3	2
811	3	4	4	3	3	4	4	4	3
812	4	4	4	4	5	4	4	4	4
813	4	4	4	5	4	4	4	4	5
814	5	5	4	4	4	4	5	4	4
815	5	3	4	3	4	5	3	4	3
816	1	2	1	1	2	1	2	1	1
817	2	2	2	3	1	2	2	2	3
818	4	5	3	4	4	5	5	3	4
819	3	4	3	4	3	3	4	3	4
820	3	2	2	2	1	3	2	2	2
821	4	4	4	4	5	4	4	4	4
822	5	4	3	3	4	4	4	3	3
823	2	1	2	2	1	3	1	2	2
824	3	3	5	5	3	4	3	5	5
825	5	3	4	4	5	5	3	4	4
826	3	4	5	4	3	5	4	5	4
827	4	4	4	4	5	3	4	4	4
828	5	5	3	4	4	3	5	3	4
829	5	4	4	3	4	5	4	4	3
830	3	4	3	4	5	4	4	3	4
831	4	4	4	5	4	4	4	4	5
832	5	3	3	4	4	5	3	3	4
833	2	2	1	2	2	2	2	1	2
834	5	4	4	3	3	5	4	4	3
835	4	5	3	4	3	3	5	3	4
836	4	4	3	4	4	4	4	3	4
837	3	5	5	3	4	4	5	5	3
838	3	4	3	4	5	3	4	3	4
839	4	5	5	5	4	3	5	5	5
840	4	4	5	3	4	4	4	5	3
841	4	4	5	3	4	5	4	5	3
842	4	3	3	4	3	5	3	3	4
843	5	3	3	5	4	3	3	3	5
844	5	4	3	3	4	3	4	3	3
845	1	1	1	1	2	2	1	1	1

846	2	2	2	2	1	2	2	2	2
847	4	5	5	3	5	5	5	5	3
848	3	4	4	3	3	4	4	4	3
849	4	4	3	5	5	4	4	3	5
850	4	4	3	4	4	5	4	3	4
851	3	4	3	5	4	4	4	3	5
852	4	4	4	4	4	4	4	4	4
853	4	4	4	3	3	4	4	4	3
854	5	4	5	4	3	5	4	5	4
855	5	3	4	3	4	4	3	4	3
856	5	3	4	3	5	5	3	4	3
857	5	4	3	5	5	5	4	3	5
858	4	4	4	3	3	3	4	4	3
859	4	5	4	3	3	5	5	4	3
860	4	5	4	5	3	4	5	4	5
861	2	3	1	2	3	3	3	1	2
862	4	4	4	4	4	3	4	4	4
863	4	4	3	3	4	3	4	3	3
864	5	4	5	4	3	5	4	5	4
865	4	4	4	5	4	3	4	4	5
866	5	3	3	3	5	3	3	3	3
867	1	3	2	1	2	3	3	2	1
868	5	4	4	3	3	5	4	4	3
869	3	5	4	5	5	5	5	4	5
870	5	3	3	3	4	4	3	3	3
871	3	4	4	5	5	3	4	4	5
872	5	4	3	4	3	5	4	3	4
873	1	1	2	2	2	2	1	2	2
874	5	3	4	3	5	4	3	4	3
875	4	3	4	4	4	3	3	4	4
876	2	2	2	3	2	2	2	2	3
877	3	3	4	4	5	5	3	4	4
878	4	4	3	4	4	4	4	3	4
879	2	2	3	2	1	2	2	3	2
880	1	1	3	2	1	1	1	3	2
881	4	3	4	3	3	3	3	4	3
882	4	5	5	4	3	4	5	5	4
883	3	3	4	4	5	4	3	4	4
884	3	5	5	4	3	4	5	5	4
885	3	5	4	4	5	3	5	4	4
886	4	4	3	5	5	3	4	3	5
887	3	4	4	4	4	3	4	4	4
888	5	4	4	4	4	4	4	4	4
889	3	4	4	4	4	4	4	4	4
890	4	5	4	4	5	4	5	4	4
891	4	3	4	4	4	5	3	4	4
892	3	5	3	3	4	3	5	3	3

893	3	5	5	4	3	4	5	5	4
894	4	5	5	4	3	3	5	5	4
895	4	3	4	3	3	5	3	4	3
896	4	3	3	3	4	5	3	3	3
897	5	5	5	3	4	4	5	5	3
898	2	2	3	1	2	3	2	3	1
899	4	4	4	4	3	3	4	4	4
900	5	4	5	4	3	4	4	5	4
901	3	4	3	4	5	4	4	3	4
902	5	4	5	3	3	5	4	5	3
903	4	4	4	5	5	4	4	4	5
904	4	4	3	3	3	5	4	3	3
905	3	3	4	5	4	5	3	4	5
906	5	5	4	4	4	4	5	4	4
907	4	4	4	4	4	4	4	4	4
908	4	4	5	3	3	4	4	5	3
909	2	2	1	2	2	2	2	1	2
910	4	5	4	4	4	4	5	4	4
911	4	5	4	4	3	4	5	4	4
912	5	4	5	3	5	4	4	5	3
913	2	1	1	2	2	2	1	1	2
914	5	4	5	5	5	4	4	5	5
915	5	3	5	3	3	4	3	5	3
916	3	2	3	3	2	2	2	3	3
917	2	2	3	2	2	2	2	3	2
918	2	1	2	2	3	3	1	2	2
919	4	5	3	4	4	4	5	3	4
920	5	5	5	4	4	4	5	5	4
921	4	4	3	4	4	4	4	3	4
922	4	3	3	5	4	4	3	3	5
923	4	4	3	5	3	3	4	3	5
924	4	4	4	3	4	3	4	4	3
925	4	5	3	4	5	4	5	3	4
926	3	4	4	5	4	5	4	4	5
927	4	4	4	4	4	3	4	4	4
928	4	5	4	4	5	3	5	4	4
929	4	3	3	3	4	5	3	3	3
930	3	4	5	3	4	4	4	5	3
931	3	5	4	3	4	4	5	4	3
932	4	5	4	4	5	4	5	4	4
933	4	3	4	5	4	4	3	4	5
934	4	4	4	4	4	4	4	4	4
935	5	3	3	3	4	4	3	3	3
936	5	4	4	5	4	5	4	4	5
937	3	2	1	3	2	3	2	1	3
938	4	4	5	4	4	5	4	5	4
939	1	2	3	1	2	2	2	3	1

940	4	4	5	4	4	4	4	5	4
941	4	4	4	4	5	4	4	4	4
942	4	4	3	4	4	3	4	3	4
943	4	4	4	5	4	4	4	4	5
944	4	4	3	3	4	4	4	3	3
945	2	1	2	1	2	2	1	2	1
946	3	4	3	3	3	3	4	3	3
947	4	5	4	4	4	4	5	4	4
948	2	1	1	2	1	1	1	1	2
949	4	4	4	4	4	4	4	4	4
950	5	4	4	5	3	5	4	4	5
951	1	3	1	2	2	1	3	1	2
952	2	2	3	2	2	1	2	3	2
953	2	2	1	1	1	1	2	1	1
954	4	5	4	4	3	4	5	4	4
955	5	3	4	4	3	4	3	4	4
956	5	4	5	5	3	4	4	5	5
957	4	4	3	3	5	4	4	3	3
958	4	4	4	5	4	5	4	4	5
959	5	3	4	3	4	5	3	4	3
960	4	4	3	4	5	4	4	3	4
961	3	4	3	4	4	4	4	3	4
962	4	4	4	4	4	3	4	4	4
963	4	5	5	3	3	3	5	5	3
964	5	5	4	4	3	5	5	4	4
965	4	3	3	4	3	4	3	3	4
966	4	3	4	4	3	4	3	4	4
967	4	4	3	3	4	5	4	3	3
968	2	1	2	1	2	1	1	2	1
969	3	5	3	4	3	4	5	3	4
970	1	2	3	1	2	2	2	3	1
971	4	4	4	4	4	4	4	4	4
972	3	5	5	5	5	3	5	5	5
973	4	4	5	3	4	3	4	5	3
974	3	5	4	5	3	5	5	4	5
975	3	4	3	4	4	4	4	3	4
976	2	2	3	1	2	3	2	3	1
977	5	3	5	5	3	5	3	5	5
978	3	4	4	3	5	3	4	4	3
979	2	2	2	2	3	2	2	2	2
980	4	4	3	4	5	3	4	3	4
981	4	3	5	3	3	3	3	5	3
982	3	5	4	4	5	4	5	4	4
983	5	5	3	4	4	4	5	3	4
984	4	4	5	4	5	4	4	5	4
985	4	4	5	5	3	3	4	5	5
986	5	4	5	4	5	4	4	5	4

987	5	4	5	4	4	4	4	5	4
988	4	4	4	3	5	4	4	4	3
989	4	3	4	4	4	5	3	4	4
990	4	3	4	4	4	4	3	4	4
991	1	2	1	2	1	1	2	1	2
992	2	1	2	2	3	2	1	2	2
993	3	3	5	5	4	4	3	5	5
994	4	3	4	4	4	5	3	4	4
995	4	3	5	3	4	4	3	5	3
996	4	4	3	4	4	4	4	3	4
997	3	3	5	4	3	3	3	5	4
998	3	3	4	5	4	4	3	4	5
999	5	4	5	4	5	4	4	5	4
1000	5	3	4	4	4	3	3	4	4
1001	4	4	3	5	4	4	4	3	5
1002	4	5	5	4	5	4	5	5	4
1003	4	3	5	3	3	5	3	5	3
1004	4	5	3	4	3	4	5	3	4
1005	5	4	5	3	5	3	4	5	3
1006	4	4	5	4	4	5	4	5	4
1007	4	4	5	5	3	5	4	5	5
1008	4	4	4	3	3	4	4	4	3
1009	1	2	3	2	2	1	2	3	2
1010	1	3	3	2	2	2	3	3	2
1011	3	4	4	4	4	4	4	4	4
1012	5	5	5	5	4	3	5	5	5
1013	3	3	3	4	4	4	3	3	4
1014	4	4	4	5	3	5	4	4	5
1015	1	2	1	2	2	2	2	1	2
1016	5	3	5	5	4	5	3	5	5
1017	3	1	3	2	2	1	1	3	2
1018	3	1	2	1	3	3	1	2	1
1019	4	4	4	3	5	5	4	4	3
1020	5	4	5	4	4	4	4	5	4
1021	4	5	4	4	5	5	5	4	4
1022	4	3	4	3	4	4	3	4	3
1023	3	3	2	2	2	2	3	2	2
1024	4	5	3	3	5	4	5	3	3
1025	4	4	3	5	4	5	4	3	5
1026	2	1	2	1	1	2	1	2	1
1027	1	1	2	1	1	2	1	2	1
1028	4	5	3	3	4	3	5	3	3
1029	4	5	4	4	4	4	5	4	4
1030	2	2	2	2	2	2	2	2	2
1031	4	4	3	4	3	3	4	3	4
1032	2	3	3	3	1	2	3	3	3
1033	4	4	4	4	5	4	4	4	4

1034	4	4	4	4	4	3	4	4	4
1035	4	5	5	4	3	4	5	5	4
1036	3	4	5	5	5	3	4	5	5
1037	4	3	3	3	4	5	3	3	3
1038	1	3	2	3	2	2	3	2	3
1039	3	4	4	4	4	5	4	4	4
1040	3	5	5	4	3	5	5	5	4
1041	3	4	5	3	3	3	4	5	3
1042	3	5	4	5	3	3	5	4	5
1043	4	4	3	5	4	4	4	3	5
1044	3	4	3	5	5	3	4	3	5
1045	4	5	4	3	3	4	5	4	3
1046	4	3	3	3	3	4	3	3	3
1047	1	2	3	1	1	2	2	3	1
1048	2	2	2	1	2	3	2	2	1
1049	5	4	5	3	5	3	4	5	3
1050	3	5	4	5	4	4	5	4	5
1051	4	5	3	3	3	4	5	3	3
1052	4	3	4	3	4	3	3	4	3
1053	4	4	3	3	4	3	4	3	3
1054	4	3	4	4	4	3	3	4	4
1055	5	4	4	3	3	4	4	4	3
1056	5	5	3	5	5	5	5	3	5
1057	4	4	3	3	5	4	4	3	3
1058	3	4	5	4	5	3	4	5	4
1059	4	4	4	4	5	5	4	4	4
1060	5	4	4	3	3	4	4	4	3
1061	3	4	5	3	4	4	4	5	3
1062	5	3	4	5	3	5	3	4	5
1063	2	3	1	2	3	1	3	1	2
1064	4	4	4	3	4	4	4	4	3
1065	4	3	4	4	5	3	3	4	4
1066	4	5	4	4	5	5	5	4	4
1067	4	3	5	3	5	3	3	5	3
1068	5	4	4	3	4	5	4	4	3
1069	3	5	3	4	3	4	5	3	4
1070	5	4	3	4	4	4	4	3	4
1071	3	5	4	4	5	4	5	4	4
1072	4	5	3	3	4	4	5	3	3
1073	4	3	5	3	4	5	3	5	3
1074	3	4	4	4	4	4	4	4	4
1075	5	5	5	3	4	3	5	5	3
1076	1	2	3	3	1	1	2	3	3
1077	1	3	1	1	2	1	3	1	1
1078	2	2	2	1	2	2	2	2	1
1079	1	2	2	2	2	2	2	2	2
1080	4	5	5	5	4	4	5	5	5

1081	3	4	5	3	4	4	4	5	3
1082	3	5	4	5	4	4	5	4	5
1083	4	3	3	3	4	3	3	3	3
1084	2	2	1	3	2	2	2	1	3
1085	3	5	5	3	3	5	5	5	3
1086	4	3	5	4	3	4	3	5	4
1087	4	4	4	3	4	3	4	4	3
1088	5	5	4	4	4	3	5	4	4
1089	3	5	4	4	5	4	5	4	4
1090	3	5	4	4	4	5	5	4	4
1091	3	3	5	3	4	4	3	5	3
1092	5	4	4	3	5	5	4	4	3
1093	4	4	5	5	4	5	4	5	5
1094	4	5	4	4	4	4	5	4	4
1095	5	5	4	4	5	4	5	4	4
1096	4	3	5	3	5	5	3	5	3
1097	3	4	3	3	4	4	4	3	3
1098	4	4	3	4	4	5	4	3	4
1099	4	3	3	5	5	4	3	3	5
1100	5	3	3	3	4	4	3	3	3
1101	4	5	3	5	4	3	5	3	5
1102	1	3	2	3	3	2	3	2	3
1103	4	3	4	4	4	4	3	4	4
1104	5	5	4	4	3	4	5	4	4
1105	5	4	4	4	5	5	4	4	4
1106	2	2	1	2	2	2	2	1	2
1107	3	4	4	4	5	5	4	4	4
1108	2	1	2	2	2	3	1	2	2
1109	4	4	3	3	4	4	4	3	3
1110	4	5	3	4	5	4	5	3	4
1111	5	5	3	5	3	3	5	3	5
1112	4	4	3	5	4	5	4	3	5
1113	4	4	3	4	4	4	4	3	4
1114	4	3	4	5	4	4	3	4	5
1115	5	4	5	4	3	3	4	5	4
1116	1	1	2	3	2	2	1	2	3
1117	5	3	3	5	3	4	3	3	5
1118	4	5	3	3	4	5	5	3	3
1119	4	4	5	5	5	4	4	5	5
1120	5	4	4	5	5	3	4	4	5
1121	2	1	1	1	1	2	1	1	1
1122	4	4	3	4	4	3	4	3	4
1123	5	4	4	4	4	4	4	4	4
1124	3	4	4	4	4	3	4	4	4
1125	3	4	4	4	4	4	4	4	4
1126	5	4	5	3	5	4	4	5	3
1127	5	5	5	5	3	3	5	5	5

1128	3	3	3	2	2	2	3	3	2
1129	4	5	4	5	4	4	5	4	5
1130	4	3	3	5	5	4	3	3	5
1131	4	5	4	5	3	4	5	4	5
1132	4	4	5	3	4	4	4	5	3
1133	5	4	4	5	5	4	4	4	5
1134	4	4	4	5	4	5	4	4	5
1135	1	2	1	2	2	2	2	1	2
1136	4	5	4	4	4	4	5	4	4
1137	3	4	3	4	4	4	4	3	4
1138	3	4	4	5	5	5	4	4	5
1139	3	5	5	4	4	3	5	5	4
1140	3	5	3	5	4	4	5	3	5
1141	4	5	3	4	3	4	5	3	4
1142	5	4	5	4	3	4	4	5	4
1143	4	3	4	5	4	4	3	4	5
1144	3	4	4	4	5	4	4	4	4
1145	3	5	4	5	3	5	5	4	5
1146	4	4	4	5	3	5	4	4	5
1147	3	4	5	4	4	3	4	5	4
1148	4	3	5	4	5	3	3	5	4
1149	4	3	3	3	3	4	3	3	3
1150	5	4	4	4	4	5	4	4	4
1151	3	3	3	2	2	3	3	3	2
1152	4	4	5	4	4	5	4	5	4
1153	3	5	4	4	4	3	5	4	4
1154	3	2	2	2	2	1	2	2	2
1155	4	3	3	5	4	4	3	3	5
1156	4	4	4	3	5	4	4	4	3
1157	3	2	3	2	2	2	2	3	2
1158	5	5	3	4	4	3	5	3	4
1159	5	5	4	3	3	5	5	4	3
1160	4	5	4	4	4	4	5	4	4
1161	4	3	3	3	4	4	3	3	3
1162	4	4	4	4	3	4	4	4	4
1163	4	3	4	4	4	5	3	4	4
1164	3	4	3	5	4	3	4	3	5
1165	4	5	4	5	4	4	5	4	5
1166	3	5	3	3	4	3	5	3	3
1167	3	4	5	3	5	3	4	5	3
1168	5	4	3	3	4	5	4	3	3
1169	2	2	2	1	2	1	2	2	1
1170	4	5	5	4	3	4	5	5	4
1171	3	4	3	3	5	4	4	3	3
1172	5	5	4	4	3	3	5	4	4
1173	5	4	5	4	3	4	4	5	4
1174	4	4	5	4	4	4	4	5	4

1175	4	4	5	4	5	3	4	5	4
1176	5	4	3	4	5	5	4	3	4
1177	3	4	5	4	4	3	4	5	4
1178	3	3	5	4	4	4	3	5	4
1179	4	5	4	5	3	3	5	4	5
1180	4	3	4	4	4	4	3	4	4
1181	5	5	4	4	4	3	5	4	4
1182	5	4	4	5	4	5	4	4	5
1183	3	2	2	3	2	3	2	2	3
1184	4	4	3	4	4	4	4	3	4
1185	3	4	3	3	4	4	4	3	3
1186	4	4	3	3	4	3	4	3	3
1187	4	4	4	3	4	3	4	4	3
1188	5	3	4	4	4	5	3	4	4
1189	3	4	5	4	4	3	4	5	4
1190	4	4	5	4	3	4	4	5	4
1191	5	4	5	3	4	5	4	5	3
1192	4	4	4	4	5	3	4	4	4
1193	4	3	3	3	4	4	3	3	3
1194	5	4	3	3	4	4	4	3	3
1195	1	3	3	3	1	2	3	3	3
1196	3	4	5	5	4	4	4	5	5
1197	4	4	3	3	3	4	4	3	3
1198	4	4	4	4	3	3	4	4	4
1199	5	4	4	5	3	3	4	4	5
1200	4	3	4	3	3	4	3	4	3
1201	3	4	3	4	3	4	4	3	4
1202	4	5	4	5	3	4	5	4	5
1203	5	5	5	5	5	4	5	5	5
1204	5	3	4	3	4	4	3	4	3
1205	3	5	3	3	4	3	5	3	3
1206	1	1	1	2	1	2	1	1	2
1207	3	5	5	4	5	4	5	5	4
1208	4	4	4	5	4	5	4	4	5
1209	4	3	4	3	5	4	3	4	3
1210	2	3	3	3	2	1	3	3	3
1211	5	5	5	4	5	3	5	5	4
1212	4	5	4	3	3	4	5	4	3
1213	2	2	2	1	1	2	2	2	1
1214	4	5	4	4	5	4	5	4	4
1215	4	4	4	4	5	5	4	4	4
1216	4	5	4	4	5	4	5	4	4
1217	3	5	3	3	3	3	5	3	3
1218	5	3	4	4	5	3	3	4	4
1219	5	4	4	3	4	5	4	4	3
1220	3	2	2	2	1	2	2	2	2
1221	2	2	2	1	2	2	2	2	1

1222	3	4	4	3	5	5	4	4	3
1223	5	5	4	5	4	3	5	4	5
1224	5	3	3	4	4	4	3	3	4
1225	3	2	2	2	1	3	2	2	2
1226	3	4	3	4	3	4	4	3	4
1227	3	5	3	4	4	3	5	3	4
1228	4	3	5	3	4	3	3	5	3
1229	5	4	5	4	4	5	4	5	4
1230	3	3	4	5	4	4	3	4	5
1231	4	3	4	5	4	4	3	4	5
1232	5	5	3	5	4	3	5	3	5
1233	4	4	5	5	5	4	4	5	5
1234	5	3	3	5	3	5	3	3	5
1235	4	4	3	3	3	4	4	3	3
1236	4	4	4	5	5	3	4	4	5
1237	4	4	4	4	4	5	4	4	4
1238	4	3	3	3	3	5	3	3	3
1239	4	5	5	4	4	4	5	5	4
1240	5	3	3	3	4	5	3	3	3
1241	3	4	4	4	4	4	4	4	4
1242	1	1	2	1	2	1	1	2	1
1243	5	4	5	4	4	3	4	5	4
1244	1	2	2	2	2	3	2	2	2
1245	1	2	2	2	2	3	2	2	2
1246	3	4	4	4	3	5	4	4	4
1247	4	4	5	4	3	4	4	5	4
1248	4	4	4	3	4	3	4	4	3
1249	4	3	4	5	3	5	3	4	5
1250	5	4	4	5	5	4	4	4	5
1251	5	5	4	3	3	4	5	4	3
1252	2	3	2	2	2	2	3	2	2
1253	5	4	3	3	4	4	4	3	3
1254	3	4	5	4	4	3	4	5	4
1255	5	4	4	4	4	4	4	4	4
1256	2	1	2	3	2	1	1	2	3
1257	3	4	3	4	3	3	4	3	4
1258	4	5	4	4	3	5	5	4	4
1259	3	3	4	4	4	3	3	4	4
1260	5	4	5	4	3	5	4	5	4
1261	5	5	4	4	3	5	5	4	4
1262	4	5	3	5	3	3	5	3	5
1263	3	1	2	2	1	2	1	2	2
1264	1	1	3	3	2	1	1	3	3
1265	3	2	2	2	2	3	2	2	2
1266	4	4	3	3	3	3	4	3	3
1267	4	3	5	5	5	5	3	5	5
1268	3	4	5	5	4	4	4	5	5

1269	3	5	3	4	5	4	5	3	4
1270	2	1	1	1	1	2	1	1	1
1271	1	1	3	2	1	2	1	3	2
1272	3	4	4	3	3	4	4	4	3
1273	4	4	4	4	5	4	4	4	4
1274	4	4	4	5	4	4	4	4	5
1275	5	5	4	4	4	4	5	4	4
1276	5	3	4	3	4	5	3	4	3
1277	1	2	1	1	2	1	2	1	1
1278	2	2	2	3	1	2	2	2	3
1279	4	5	3	4	4	5	5	3	4
1280	3	4	3	4	3	3	4	3	4
1281	3	2	2	2	1	3	2	2	2
1282	4	4	4	4	5	4	4	4	4
1283	5	4	3	3	4	4	4	3	3
1284	2	1	2	2	1	3	1	2	2
1285	3	3	5	5	3	4	3	5	5
1286	5	3	4	4	5	5	3	4	4
1287	3	4	5	4	3	5	4	5	4
1288	4	4	4	4	5	3	4	4	4
1289	5	5	3	4	4	3	5	3	4
1290	5	4	4	3	4	5	4	4	3
1291	3	4	3	4	5	4	4	3	4
1292	4	4	4	5	4	4	4	4	5
1293	5	3	3	4	4	5	3	3	4
1294	2	2	1	2	2	2	2	1	2
1295	5	4	4	3	3	5	4	4	3
1296	4	5	3	4	3	3	5	3	4
1297	4	4	3	4	4	4	4	3	4
1298	3	5	5	3	4	4	5	5	3
1299	3	4	3	4	5	3	4	3	4
1300	4	5	5	5	4	3	5	5	5
1301	4	4	5	3	4	4	4	5	3
1302	4	4	5	3	4	5	4	5	3
1303	4	3	3	4	3	5	3	3	4
1304	5	3	3	5	4	3	3	3	5
1305	5	4	3	3	4	3	4	3	3
1306	1	1	1	1	2	2	1	1	1
1307	2	2	2	2	1	2	2	2	2
1308	4	5	5	3	5	5	5	5	3
1309	3	4	4	3	3	4	4	4	3
1310	4	4	3	5	5	4	4	3	5
1311	4	4	3	4	4	5	4	3	4
1312	3	4	3	5	4	4	4	3	5
1313	4	4	4	4	4	4	4	4	4
1314	4	4	4	3	3	4	4	4	3
1315	5	4	5	4	3	5	4	5	4

1316	5	3	4	3	4	4	3	4	3
1317	5	3	4	3	5	5	3	4	3
1318	5	4	3	5	5	5	4	3	5
1319	4	4	4	3	3	3	4	4	3
1320	4	5	4	3	3	5	5	4	3
1321	4	5	4	5	3	4	5	4	5
1322	2	3	1	2	3	3	3	1	2
1323	4	4	4	4	4	3	4	4	4
1324	4	4	3	3	4	3	4	3	3
1325	5	4	5	4	3	5	4	5	4
1326	4	4	4	5	4	3	4	4	5
1327	5	3	3	3	5	3	3	3	3
1328	1	3	2	1	2	3	3	2	1
1329	5	4	4	3	3	5	4	4	3
1330	3	5	4	5	5	5	5	4	5
1331	5	3	3	3	4	4	3	3	3
1332	3	4	4	5	5	3	4	4	5
1333	5	4	3	4	3	5	4	3	4
1334	1	1	2	2	2	2	1	2	2
1335	5	3	4	3	5	4	3	4	3
1336	4	3	4	4	4	3	3	4	4
1337	2	2	2	3	2	2	2	2	3
1338	3	3	4	4	5	5	3	4	4
1339	4	4	3	4	4	4	4	3	4
1340	2	2	3	2	1	2	2	3	2
1341	1	1	3	2	1	1	1	3	2
1342	4	3	4	3	3	3	3	4	3
1343	4	5	5	4	3	4	5	5	4
1344	3	3	4	4	5	4	3	4	4
1345	3	5	5	4	3	4	5	5	4
1346	3	5	4	4	5	3	5	4	4
1347	4	4	3	5	5	3	4	3	5
1348	3	4	4	4	4	3	4	4	4
1349	5	4	4	4	4	4	4	4	4
1350	3	4	4	4	4	4	4	4	4
1351	4	5	4	4	5	4	5	4	4
1352	4	3	4	4	4	5	3	4	4
1353	3	5	3	3	4	3	5	3	3
1354	3	5	5	4	3	4	5	5	4
1355	4	5	5	4	3	3	5	5	4
1356	4	3	4	3	3	5	3	4	3
1357	4	3	3	3	4	5	3	3	3
1358	5	5	5	3	4	4	5	5	3
1359	2	2	3	1	2	3	2	3	1
1360	4	4	4	4	3	3	4	4	4
1361	5	4	5	4	3	4	4	5	4
1362	3	4	3	4	5	4	4	3	4

1363	5	4	5	3	3	5	4	5	3
1364	4	4	4	5	5	4	4	4	5
1365	4	4	3	3	3	5	4	3	3
1366	3	3	4	5	4	5	3	4	5
1367	5	5	4	4	4	4	5	4	4
1368	4	4	4	4	4	4	4	4	4
1369	4	4	5	3	3	4	4	5	3
1370	2	2	1	2	2	2	2	1	2
1371	4	5	4	4	4	4	5	4	4
1372	4	5	4	4	3	4	5	4	4
1373	5	4	5	3	5	4	4	5	3
1374	2	1	1	2	2	2	1	1	2
1375	5	4	5	5	5	4	4	5	5
1376	5	3	5	3	3	4	3	5	3
1377	3	2	3	3	2	2	2	3	3
1378	2	2	3	2	2	2	2	3	2
1379	2	1	2	2	3	3	1	2	2
1380	4	5	3	4	4	4	5	3	4
1381	5	5	5	4	4	4	5	5	4
1382	4	4	3	4	4	4	4	3	4
1383	4	3	3	5	4	4	3	3	5
1384	4	5	5	4	3	3	5	5	4
1385	4	3	4	3	3	5	3	4	3
1386	4	3	3	3	4	5	3	3	3
1387	5	5	5	3	4	4	5	5	3
1388	2	2	3	1	2	3	2	3	1
1389	4	4	4	4	3	3	4	4	4
1390	5	4	5	4	3	4	4	5	4
1391	3	4	3	4	5	4	4	3	4
1392	5	4	5	3	3	5	4	5	3
1393	4	4	4	5	5	4	4	4	5
1394	4	4	3	3	3	5	4	3	3
1395	3	3	4	5	4	5	3	4	5
1396	5	5	4	4	4	4	5	4	4
1397	4	4	4	4	4	4	4	4	4
1398	4	4	5	3	3	4	4	5	3
1399	2	2	1	2	2	2	2	1	2
1400	4	5	4	4	4	4	5	4	4
1401	4	5	4	4	3	4	5	4	4
1402	5	4	5	3	5	4	4	5	3
1403	2	1	1	2	2	2	1	1	2
1404	5	4	5	5	5	4	4	5	5
1405	5	3	5	3	3	4	3	5	3
1406	3	2	3	3	2	2	2	3	3
1407	2	2	3	2	2	2	2	3	2
1408	2	1	2	2	3	3	1	2	2
1409	4	5	3	4	4	4	5	3	4

1410	5	5	5	4	4	4	5	5	4
1411	4	4	3	4	4	4	4	3	4
1412	4	3	3	5	4	4	3	3	5
1413	4	5	5	4	3	3	5	5	4
1414	4	3	4	3	3	5	3	4	3
1415	4	3	3	3	4	5	3	3	3
1416	5	5	5	3	4	4	5	5	3
1417	2	2	3	1	2	3	2	3	1
1418	4	4	4	4	3	3	4	4	4
1419	5	4	5	4	3	4	4	5	4
1420	3	4	3	4	5	4	4	3	4
1421	5	4	5	3	3	5	4	5	3
1422	4	4	4	5	5	4	4	4	5
1423	4	4	3	3	3	5	4	3	3
1424	3	3	4	5	4	5	3	4	5
1425	5	5	4	4	4	4	5	4	4
1426	4	4	4	4	4	4	4	4	4
1427	4	4	5	3	3	4	4	5	3
1428	2	2	1	2	2	2	2	1	2
1429	4	5	4	4	4	4	5	4	4
1430	4	5	4	4	3	4	5	4	4
1431	5	4	5	3	5	4	4	5	3
1432	2	1	1	2	2	2	1	1	2
1433	5	4	5	5	5	4	4	5	5
1434	5	3	5	3	3	4	3	5	3
1435	3	2	3	3	2	2	2	3	3
1436	2	2	3	2	2	2	2	3	2
1437	2	1	2	2	3	3	1	2	2
1438	4	5	3	4	4	4	5	3	4
1439	5	5	5	4	4	4	5	5	4
1440	4	4	3	4	4	4	4	3	4
1441	4	3	3	5	4	4	3	3	5
1442	4	5	5	4	3	3	5	5	4
1443	4	3	4	3	3	5	3	4	3
1444	4	3	3	3	4	5	3	3	3
1445	5	5	5	3	4	4	5	5	3
1446	2	2	3	1	2	3	2	3	1
1447	4	4	4	4	3	3	4	4	4
1448	5	4	5	4	3	4	4	5	4
1449	3	4	3	4	5	4	4	3	4
1450	5	4	5	3	3	5	4	5	3
1451	4	4	4	5	5	4	4	4	5
1452	4	4	3	3	3	5	4	3	3
1453	3	3	4	5	4	5	3	4	5
1454	5	5	4	4	4	4	5	4	4
1455	4	4	4	4	4	4	4	4	4
1456	4	4	5	3	3	4	4	5	3

1457	2	2	1	2	2	2	2	1	2
1458	4	5	4	4	4	4	5	4	4
1459	4	5	4	4	3	4	5	4	4
1460	5	4	5	3	5	4	4	5	3
1461	2	1	1	2	2	2	1	1	2
1462	5	4	5	5	5	4	4	5	5
1463	5	3	5	3	3	4	3	5	3
1464	3	2	3	3	2	2	2	3	3
1465	2	2	3	2	2	2	2	3	2
1466	2	1	2	2	3	3	1	2	2
1467	4	5	3	4	4	4	5	3	4
1468	5	5	5	4	4	4	5	5	4
1469	4	4	3	4	4	4	4	3	4
1470	4	3	3	5	4	4	3	3	5
1471	3	2	3	3	2	2	2	3	3
1472	2	2	3	2	2	2	2	3	2
1473	2	1	2	2	3	3	1	2	2
1474	4	5	3	4	4	4	5	3	4
1475	5	5	5	4	4	4	5	5	4
1476	4	4	3	4	4	4	4	3	4
1477	4	3	3	5	4	4	3	3	5
1478	3	2	3	3	2	2	2	3	3
1479	2	2	3	2	2	2	2	3	2
1480	2	1	2	2	3	3	1	2	2
1481	4	5	3	4	4	4	5	3	4
1482	2	1	2	2	3	3	1	2	2

BPS_1	BPS_2	BPS_3	BPS_4	BPS_5	BPS_6	BPS_7	BPS_8	BPS_9	IAS_1
4	5	4	4	4	4	4	4	3	4
1	2	1	2	1	2	4	4	4	4
5	4	4	5	5	4	4	5	3	5
4	4	4	4	4	4	3	4	4	4
4	5	4	4	3	4	4	4	4	5
4	4	4	4	3	5	4	5	4	3
3	3	4	3	4	4	4	3	3	4
4	4	3	5	3	5	3	4	5	3
5	4	3	4	5	3	3	5	4	4
4	4	5	5	4	3	4	5	4	4
4	5	4	5	4	4	4	3	4	4
3	4	4	4	4	4	4	4	4	4
5	4	4	3	5	4	5	3	3	3
4	3	3	4	5	4	5	4	4	4
1	1	3	2	3	2	3	2	1	1
4	4	3	5	4	3	4	4	5	3
3	1	2	2	3	2	1	2	3	2
4	4	4	5	5	5	4	4	5	4
4	3	4	5	3	4	4	4	4	5
5	3	5	3	4	4	4	4	3	5
3	5	5	4	4	4	4	4	4	4
4	5	4	4	4	3	4	4	3	1
1	3	2	2	3	2	2	1	2	2
4	5	5	4	3	5	3	4	3	4
5	5	3	5	5	4	4	5	4	1
5	3	5	3	4	4	2	1	1	4
5	3	4	4	4	4	4	4	4	4
4	4	3	5	3	4	5	4	4	4
2	2	2	2	2	2	1	3	1	3
2	2	2	2	2	2	2	2	3	1
4	4	3	4	4	3	2	2	1	5
4	3	5	4	4	4	4	5	4	5
4	4	5	5	5	3	5	3	4	5
2	2	1	2	1	1	5	4	5	4
5	4	4	3	3	4	4	4	3	5
5	4	4	3	5	4	4	4	4	3
3	4	4	3	4	3	5	3	4	4
5	4	4	4	4	3	4	4	3	5
5	4	4	4	4	5	3	4	3	5
5	5	3	5	5	3	4	4	4	4
3	4	5	3	4	4	4	5	5	4
4	3	4	5	4	3	5	5	4	3
4	5	4	5	4	4	4	3	3	5
4	4	5	3	3	5	4	3	4	4
4	5	3	3	3	4	4	4	3	4
2	1	2	2	3	1	2	1	2	1

4	4	3	3	3	4	3	5	3	4
2	1	2	3	2	2	1	2	3	3
4	3	4	4	3	3	4	4	4	4
3	5	3	4	3	3	3	5	5	5
4	4	5	4	4	3	4	4	5	3
3	4	3	4	3	4	3	5	4	4
4	5	5	4	3	3	3	4	3	4
1	2	1	2	1	3	2	2	3	3
5	5	4	5	3	4	5	3	5	4
4	4	3	4	4	5	3	4	4	4
2	3	3	3	2	2	2	2	2	2
3	3	3	4	5	4	4	4	3	4
3	4	4	5	3	4	4	3	5	4
4	5	5	4	5	5	3	5	4	4
5	4	4	4	4	3	5	5	3	5
5	4	4	3	3	4	4	4	5	4
4	5	4	4	4	5	4	4	5	2
4	4	4	3	5	4	5	4	5	4
4	4	4	4	3	5	5	4	5	3
4	4	5	4	4	5	4	4	4	3
3	4	4	3	4	4	4	3	4	4
4	4	4	3	3	3	4	3	4	3
3	4	3	5	3	5	1	2	1	4
3	2	2	3	2	2	2	1	2	3
4	5	5	4	5	5	3	3	5	5
4	3	5	4	3	4	4	3	4	5
3	4	4	4	5	4	4	3	5	3
5	4	4	5	3	5	4	4	3	5
3	5	4	4	3	3	3	3	5	5
4	4	4	4	4	4	3	3	4	4
5	5	5	5	5	4	5	4	5	3
3	4	5	4	4	5	5	3	4	4
3	4	3	4	5	4	4	4	3	4
3	4	4	4	4	4	4	5	5	4
4	5	5	4	5	5	4	3	5	5
2	2	1	1	2	1	4	5	3	4
4	4	4	3	4	5	5	4	5	4
5	4	5	4	4	5	4	4	5	4
5	4	4	5	4	4	4	4	5	4
4	4	5	3	5	5	4	4	4	4
3	2	2	2	2	2	1	2	3	1
3	2	1	2	2	2	1	3	3	2
4	4	4	3	3	4	3	4	4	5
3	4	5	4	4	3	5	5	5	4
5	4	3	4	3	4	3	3	3	4
4	5	4	3	3	5	4	4	4	4
3	4	5	5	3	3	1	2	1	3

4	4	5	4	5	5	5	3	5	4
1	2	2	1	2	3	3	1	3	2
2	2	1	2	3	1	3	1	2	1
4	5	5	4	4	4	4	4	4	4
4	5	3	5	3	3	5	4	5	3
4	4	5	4	4	4	4	5	4	1
5	3	4	4	3	4	4	3	4	4
2	2	2	3	1	2	3	3	2	2
4	4	5	4	3	5	4	5	3	4
4	4	5	5	4	4	4	4	3	4
1	3	2	2	2	2	2	1	2	3
3	5	4	5	4	4	1	1	2	5
5	5	4	3	3	4	4	5	3	3
5	5	3	4	5	4	4	5	4	5
4	4	3	4	3	3	2	2	2	5
4	5	3	4	4	5	4	4	3	4
2	3	2	1	1	2	2	3	3	2
4	4	4	4	3	4	4	4	4	4
5	5	5	4	4	5	4	4	4	5
4	4	4	3	3	3	4	5	5	3
4	3	3	5	4	5	3	4	5	4
5	4	3	4	4	5	4	3	3	5
3	2	2	3	2	2	1	3	2	3
4	4	5	4	3	4	3	4	4	3
3	5	3	4	3	4	3	5	5	5
4	4	5	5	3	5	3	4	5	4
5	5	3	3	3	5	3	5	4	3
4	5	4	3	4	5	4	4	3	5
5	5	4	4	5	3	3	4	3	4
5	5	5	4	3	5	4	5	4	5
2	1	1	2	1	1	4	3	3	3
2	2	2	3	3	1	1	2	3	3
2	2	3	3	3	1	2	2	2	1
5	3	4	5	5	5	5	4	5	5
5	4	4	5	4	4	3	5	4	3
3	3	5	4	4	5	4	5	3	5
4	4	4	3	4	5	4	3	4	3
4	4	4	4	4	4	4	4	3	4
4	5	4	4	4	4	4	3	4	5
3	3	4	4	4	5	5	4	4	4
5	4	3	3	3	4	5	5	3	4
3	5	4	3	5	4	4	4	3	4
2	1	1	2	1	2	3	4	5	3
4	4	5	4	3	4	4	4	4	3
4	4	4	4	4	3	5	4	4	3
5	4	3	3	3	5	3	4	5	5
4	4	4	3	4	3	5	3	4	4

2	2	2	1	1	2	2	3	1	2
3	4	4	4	4	3	4	4	4	5
4	5	4	5	5	4	4	3	4	3
3	5	5	4	4	3	4	5	4	4
4	5	5	4	4	3	4	3	5	5
4	4	5	3	4	4	5	4	4	3
5	5	3	3	4	3	3	5	3	3
2	2	1	2	2	2	5	4	3	4
4	3	4	5	4	4	3	5	4	4
3	4	5	4	4	4	4	5	3	4
4	5	4	5	4	4	4	3	5	3
4	3	5	4	3	4	3	4	4	2
1	1	2	1	2	1	5	5	5	4
2	1	2	2	2	2	1	2	3	2
2	2	2	1	3	3	1	3	1	2
4	4	4	4	3	4	2	2	2	5
2	2	1	2	2	3	1	2	2	1
4	3	5	4	4	4	4	5	5	5
3	4	5	5	4	5	3	4	5	3
3	4	4	3	5	5	3	5	4	5
4	4	4	4	5	4	4	3	3	4
1	2	2	2	1	3	2	2	1	2
3	5	4	5	4	4	3	5	5	1
3	4	5	5	3	4	4	3	5	4
4	3	5	3	4	3	4	4	4	3
4	4	4	4	4	4	5	5	4	3
5	4	3	3	4	4	3	5	4	4
4	5	4	5	3	4	3	5	4	3
5	4	4	3	5	5	3	3	5	4
5	3	3	3	4	4	5	4	4	4
4	4	3	4	4	3	4	4	5	5
5	4	5	3	4	3	4	5	4	3
5	4	4	4	3	5	5	5	4	1
3	5	5	4	4	4	4	3	5	2
5	3	4	4	4	3	3	4	3	3
5	3	3	5	4	5	4	4	3	4
3	4	3	4	4	4	4	3	3	3
5	4	4	4	4	3	5	3	3	4
3	3	4	4	5	4	4	5	3	4
2	3	3	3	1	2	1	3	2	2
4	3	5	4	5	4	4	3	4	5
3	4	5	4	5	5	5	5	4	5
4	4	5	3	4	3	5	4	4	2
1	2	2	2	2	2	2	2	1	3
4	4	4	4	4	5	3	4	4	4
3	2	1	3	2	1	2	1	2	2
4	4	5	4	4	4	4	4	3	4

3	4	3	5	4	5	4	5	3	5
3	4	4	4	5	4	5	5	3	5
4	3	3	4	4	4	4	4	3	4
3	4	4	4	4	3	4	4	3	5
5	4	5	4	3	4	4	3	4	3
1	2	2	1	2	2	5	4	5	3
1	3	1	3	2	3	1	1	2	1
5	3	4	4	3	4	5	3	3	5
4	3	3	4	4	4	4	5	3	4
5	4	3	5	5	4	4	4	5	4
5	5	5	3	3	5	5	4	4	4
3	1	2	3	3	1	2	1	1	2
5	4	5	4	4	3	4	4	3	5
3	4	4	4	5	4	5	4	4	5
3	5	5	3	3	3	3	4	4	5
3	4	4	4	4	3	3	4	4	5
1	2	1	2	1	2	5	4	5	5
4	5	4	3	5	4	5	5	5	5
3	2	1	1	2	2	3	3	3	1
4	4	3	5	4	4	4	5	4	5
4	5	3	4	4	3	4	3	3	5
4	4	4	3	5	4	4	5	4	5
3	4	4	5	4	3	4	4	5	4
5	4	3	5	3	4	5	4	4	3
5	3	4	4	5	4	4	4	4	3
4	4	3	5	5	4	1	2	1	3
4	4	4	4	3	3	4	5	4	4
3	4	4	3	3	4	3	4	3	5
5	4	4	4	4	4	3	4	4	3
4	4	5	4	4	4	3	5	5	4
3	5	4	3	4	3	3	5	3	3
3	4	3	5	4	4	4	5	3	4
5	4	4	4	4	3	5	4	5	4
4	4	4	5	4	4	4	3	4	3
3	5	5	4	4	5	3	4	4	4
5	4	4	4	4	5	3	5	4	3
4	5	3	4	5	4	4	4	4	4
4	5	3	4	4	4	3	4	5	3
4	4	5	4	5	5	4	3	5	5
4	3	5	5	3	4	4	3	3	4
4	3	4	4	4	3	5	4	4	4
2	2	1	1	2	2	3	3	3	1
5	4	3	4	4	5	4	4	5	4
4	3	5	3	5	3	3	5	4	4
3	2	3	2	2	3	3	2	2	2
3	3	4	5	3	4	4	3	3	3
4	5	4	3	4	4	4	4	4	5

2	2	1	3	3	2	3	2	3	1
4	4	4	3	4	5	5	5	3	4
4	4	4	4	5	5	5	5	4	4
5	5	4	5	3	3	4	5	4	5
3	4	3	4	4	4	4	3	3	5
3	3	3	3	5	4	4	4	4	3
3	4	5	5	4	4	4	3	4	4
3	5	4	4	3	4	3	4	3	4
4	4	5	5	4	4	4	5	4	3
4	4	3	4	3	5	3	5	3	4
5	3	4	4	3	4	3	4	5	4
4	4	3	3	4	4	5	4	3	4
2	3	1	2	1	3	2	2	2	2
5	3	5	5	4	3	4	5	5	3
5	4	4	4	5	3	3	4	3	5
3	5	5	4	4	5	5	5	4	3
3	3	3	3	4	4	5	4	5	4
4	5	4	4	3	5	4	4	5	4
4	5	3	3	3	5	4	4	5	4
3	4	3	5	3	4	5	4	3	4
4	3	4	4	5	5	3	4	5	4
4	4	5	3	4	3	3	3	5	4
5	5	4	4	4	5	4	5	4	5
4	4	4	4	3	5	4	3	4	4
4	5	5	3	5	3	5	5	4	5
5	5	3	3	4	5	5	4	4	3
1	2	2	2	2	1	3	2	2	2
5	3	4	4	4	3	4	4	3	4
5	3	4	3	5	4	3	4	3	4
5	3	4	5	4	5	4	4	3	4
4	4	4	4	3	4	4	4	4	4
3	4	5	4	5	4	5	3	4	4
5	5	4	5	5	4	3	4	5	4
5	3	3	5	3	3	4	4	5	4
5	3	5	5	3	3	5	4	5	5
5	5	3	5	4	4	4	4	4	4
4	4	3	3	5	4	4	3	3	3
4	5	5	5	4	5	5	4	3	4
2	2	2	2	3	2	1	3	3	2
4	4	4	5	4	4	3	4	5	5
4	3	4	3	5	4	4	4	3	5
4	4	4	4	5	5	4	4	4	1
3	4	4	5	4	5	5	4	4	5
4	4	3	4	4	5	4	3	4	5
1	1	2	1	2	1	3	4	3	4
5	4	4	4	3	4	4	5	4	4
3	5	3	4	4	3	5	5	5	4

4	4	4	4	3	3	5	3	4	5
1	2	1	1	1	2	3	5	3	4
3	4	3	4	3	5	1	1	1	4
4	4	5	4	4	4	3	5	5	5
4	3	5	3	4	4	4	4	4	5
5	5	4	4	4	3	4	3	4	3
3	3	1	1	3	2	2	3	3	1
4	3	4	4	3	4	5	5	5	5
4	4	5	3	4	4	4	5	4	4
3	4	4	4	3	5	2	2	2	4
4	4	4	4	5	4	4	5	4	4
4	5	4	4	3	4	4	4	4	4
4	3	3	4	3	3	4	5	4	4
3	5	4	4	5	5	3	5	3	3
3	4	5	4	5	3	5	3	4	3
4	4	5	4	5	4	5	4	4	4
3	1	2	3	2	1	3	2	2	3
3	4	4	5	5	5	2	2	2	5
4	3	4	4	4	3	3	4	4	1
4	3	5	4	4	4	5	5	4	5
4	4	4	5	3	5	5	3	3	2
3	2	2	2	2	2	3	2	2	2
1	2	2	2	2	2	3	4	3	3
5	4	3	4	4	4	3	5	3	5
4	4	5	4	4	3	4	3	5	4
5	4	4	5	4	3	5	4	5	5
4	5	4	3	5	4	3	3	4	4
4	5	3	4	4	5	4	3	4	4
5	4	4	4	3	4	5	5	3	3
4	5	5	4	4	3	4	4	5	4
4	3	4	4	3	4	5	3	3	4
4	4	3	3	5	4	4	4	3	5
4	5	4	4	3	3	4	4	4	4
4	4	5	4	3	4	4	4	4	4
4	3	4	4	4	3	4	3	3	5
2	1	2	1	2	2	4	5	5	3
4	4	3	3	5	4	5	3	3	4
4	5	3	3	3	4	3	4	4	5
5	4	4	5	4	4	1	1	2	4
4	4	4	5	4	5	5	4	5	3
2	2	1	2	1	2	1	2	2	2
3	1	1	2	3	2	1	2	2	1
4	4	4	5	3	3	3	4	4	3
5	4	5	4	3	4	4	4	5	5
3	4	3	4	5	3	4	4	4	5
4	5	4	4	5	5	4	3	4	3
3	4	4	5	4	3	5	4	4	4

5	4	3	5	3	4	5	5	4	5
2	1	1	2	2	2	2	3	2	3
4	5	5	5	3	4	5	4	3	3
3	5	4	4	4	3	3	4	5	1
5	4	4	5	4	4	5	4	4	3
1	1	3	3	2	2	2	1	2	2
4	5	4	4	4	5	3	4	3	4
4	5	4	3	4	3	4	5	4	3
3	4	4	4	4	3	3	3	4	4
3	3	4	5	3	4	5	4	5	4
3	4	3	4	4	3	5	5	4	2
3	5	3	4	4	5	4	5	3	4
2	2	1	2	2	1	3	1	2	1
2	3	3	3	2	3	1	1	3	2
2	1	2	2	1	1	3	2	2	2
4	3	3	5	4	5	4	4	3	4
3	5	4	3	5	5	4	3	5	4
4	5	3	3	4	4	3	4	5	3
1	1	2	1	2	1	3	5	3	3
5	5	4	5	5	5	2	1	1	4
3	3	2	2	2	1	1	1	3	2
3	4	5	3	5	3	3	4	4	4
4	5	4	3	4	4	4	4	4	3
3	3	4	5	5	3	4	4	4	3
4	4	5	5	5	4	5	5	4	5
4	4	4	4	4	4	5	3	4	5
4	4	4	4	4	4	1	2	1	5
1	2	3	2	2	3	2	2	2	2
4	5	4	4	4	3	4	5	3	5
4	3	4	5	3	3	3	4	3	5
2	2	1	3	3	2	3	2	2	1
1	1	1	2	1	2	4	4	4	4
1	1	1	2	1	1	5	4	3	4
2	2	2	3	2	2	2	1	2	3
2	1	2	1	1	2	3	3	5	4
3	4	4	4	3	3	5	3	4	4
4	5	5	4	4	4	3	4	5	3
5	4	4	4	3	4	4	4	4	4
5	4	5	4	5	4	5	5	3	4
3	4	3	5	4	5	5	4	4	4
3	5	3	5	4	4	3	4	3	5
5	4	4	4	4	4	4	4	4	5
3	3	3	4	4	4	5	3	3	5
2	1	2	2	3	2	2	2	1	2
4	4	4	4	5	5	5	4	4	4
3	4	5	5	3	4	4	5	3	3
3	5	4	3	4	3	4	4	3	3

4	4	3	5	4	5	3	5	5	4
4	3	4	3	3	4	3	4	3	3
4	4	4	4	4	3	4	5	5	4
4	3	3	4	3	4	4	4	5	4
4	3	3	4	4	5	4	4	5	5
5	5	4	4	5	5	4	3	3	3
3	4	3	4	4	3	5	3	3	4
5	3	3	4	3	4	5	4	3	5
3	5	5	4	5	4	1	1	1	4
3	3	5	4	5	4	2	2	2	5
3	4	4	3	4	4	4	5	5	4
4	5	3	5	4	5	3	4	4	3
4	4	4	3	5	4	4	4	3	4
2	1	1	2	2	1	4	4	3	4
4	5	3	5	4	4	3	4	3	5
4	3	4	4	3	3	4	4	4	4
3	4	5	5	4	4	4	4	4	4
3	4	4	5	4	3	5	4	5	4
5	4	5	5	4	4	5	3	4	4
4	3	4	5	5	4	5	3	4	4
3	4	3	4	4	5	5	4	3	4
4	3	5	5	4	4	4	4	4	4
4	3	5	5	3	4	4	5	4	4
3	5	3	4	5	4	4	5	4	4
2	2	2	2	2	1	2	3	1	3
5	3	4	4	4	4	4	4	4	1
4	3	5	4	4	3	4	4	3	2
5	4	5	3	4	4	5	4	5	4
5	4	4	5	4	5	4	4	4	5
5	5	3	4	5	5	5	3	3	4
2	3	1	2	3	2	1	3	2	2
4	4	4	5	5	4	5	4	4	5
5	5	4	4	3	4	3	5	4	3
3	4	5	3	5	4	5	3	3	3
5	4	4	3	4	4	3	4	4	4
4	5	4	3	5	4	5	4	3	4
4	4	5	4	4	4	1	1	2	4
3	4	4	4	5	5	5	3	4	3
4	5	4	4	5	5	4	3	4	5
1	2	2	2	1	3	2	2	2	3
5	4	3	4	5	3	3	3	4	4
4	4	5	3	4	4	4	4	3	4
2	2	1	3	3	1	2	2	3	2
2	2	1	2	2	2	1	1	3	1
2	1	2	1	2	2	4	3	4	4
4	4	4	5	4	5	4	5	5	3
4	4	4	3	4	3	3	3	4	4

4	5	5	5	4	3	3	5	5	3
4	4	5	5	4	4	3	5	4	5
5	3	4	4	3	3	4	4	3	5
4	4	5	4	4	3	3	4	4	4
4	4	3	5	4	5	5	4	4	4
4	3	5	4	3	3	3	4	4	4
3	3	4	3	4	4	4	5	4	4
3	5	3	4	5	4	4	3	4	4
4	4	4	4	4	4	3	5	3	4
4	5	4	3	4	5	3	5	5	5
4	4	4	3	4	3	4	5	5	3
4	4	4	5	4	3	4	3	4	4
1	1	1	2	2	2	4	3	3	4
4	4	5	3	4	4	5	5	5	2
3	2	1	3	1	1	2	2	3	1
3	4	3	4	5	3	4	4	4	4
5	3	3	3	4	4	5	4	5	4
5	4	4	4	5	4	3	4	3	3
3	4	4	3	4	3	5	4	5	1
3	4	4	5	4	5	4	4	4	3
4	4	4	3	5	3	4	4	3	3
3	3	5	4	3	4	3	3	4	4
4	4	5	5	4	4	5	5	4	5
4	5	3	5	3	3	4	4	4	3
3	5	5	5	4	4	4	4	5	5
1	1	2	2	3	2	2	2	1	1
4	5	3	3	5	3	4	5	4	4
5	4	3	4	4	4	4	5	4	4
4	5	4	4	3	4	5	4	5	3
4	4	3	4	3	3	2	1	1	4
4	4	5	4	4	5	5	4	5	4
5	3	3	4	5	5	5	3	5	4
2	3	1	1	1	2	3	2	3	2
2	3	3	2	2	2	2	2	3	3
1	2	2	1	2	2	2	1	2	1
4	3	3	4	4	5	4	5	3	5
3	3	4	5	4	4	5	5	5	4
3	3	3	4	3	3	4	4	3	3
4	3	4	4	5	4	4	3	3	3
4	5	4	4	4	4	4	4	3	4
1	2	1	2	1	2	4	4	4	4
5	4	4	5	5	4	4	5	3	5
4	4	4	4	4	4	3	4	4	4
4	5	4	4	3	4	4	4	4	5
4	4	4	4	3	5	4	5	4	3
3	3	4	3	4	4	4	3	3	4
4	4	3	5	3	5	3	4	5	3

5	4	3	4	5	3	3	5	4	4
4	4	5	5	4	3	4	5	4	4
4	5	4	5	4	4	4	3	4	4
3	4	4	4	4	4	4	4	4	4
5	4	4	3	5	4	5	3	3	3
4	3	3	4	5	4	5	4	4	4
1	1	3	2	3	2	3	2	1	1
4	4	3	5	4	3	4	4	5	3
3	1	2	2	3	2	1	2	3	2
4	4	4	5	5	5	4	4	5	4
4	3	4	5	3	4	4	4	4	5
5	3	5	3	4	4	4	4	3	5
3	5	5	4	4	4	4	4	4	4
4	5	4	4	4	3	4	4	3	1
1	3	2	2	3	2	2	1	2	2
4	5	5	4	3	5	3	4	3	4
5	5	3	5	5	4	4	5	4	1
5	3	5	3	4	4	2	1	1	4
5	3	4	4	4	4	4	4	4	4
4	4	3	5	3	4	5	4	4	4
2	2	2	2	2	2	1	3	1	3
2	2	2	2	2	2	2	2	3	1
4	4	3	4	4	3	2	2	1	5
4	3	5	4	4	4	4	5	4	5
4	4	5	5	5	3	5	3	4	5
2	2	1	2	1	1	5	4	5	4
5	4	4	3	3	4	4	4	3	5
5	4	4	3	5	4	4	4	4	3
3	4	4	3	4	3	5	3	4	4
5	4	4	4	4	3	4	4	3	5
5	4	4	4	4	5	3	4	3	5
5	5	3	5	5	3	4	4	4	4
3	4	5	3	4	4	4	5	5	4
4	3	4	5	4	3	5	5	4	3
4	5	4	5	4	4	4	3	3	5
4	4	5	3	3	5	4	3	4	4
4	5	3	3	3	4	4	4	3	4
2	1	2	2	3	1	2	1	2	1
4	4	3	3	3	4	3	5	3	4
2	1	2	3	2	2	1	2	3	3
4	3	4	4	3	3	4	4	4	4
3	5	3	4	3	3	3	5	5	5
4	4	5	4	4	3	4	4	5	3
3	4	3	4	3	4	3	5	4	4
4	5	5	4	3	3	3	4	3	4
1	2	1	2	1	3	2	2	3	3
5	5	4	5	3	4	5	3	5	4

4	4	3	4	4	5	3	4	4	4
2	3	3	3	2	2	2	2	2	2
3	3	3	4	5	4	4	4	3	4
3	4	4	5	3	4	4	3	5	4
4	5	5	4	5	5	3	5	4	4
5	4	4	4	4	3	5	5	3	5
5	4	4	3	3	4	4	4	5	4
4	5	4	4	4	5	4	4	5	2
4	4	4	3	5	4	5	4	5	4
4	4	4	4	3	5	5	4	5	3
4	4	5	4	4	5	4	4	4	3
3	4	4	3	4	4	4	3	4	4
4	4	4	3	3	3	4	3	4	3
3	4	3	5	3	5	1	2	1	4
3	2	2	3	2	2	2	1	2	3
4	5	5	4	5	5	3	3	5	5
4	3	5	4	3	4	4	3	4	5
3	4	4	4	5	4	4	3	5	3
5	4	4	5	3	5	4	4	3	5
3	5	4	4	3	3	3	3	5	5
4	4	4	4	4	4	3	3	4	4
5	5	5	5	5	4	5	4	5	3
3	4	5	4	4	5	5	3	4	4
3	4	3	4	5	4	4	4	3	4
3	4	4	4	4	4	4	5	5	4
4	5	5	4	5	5	4	3	5	5
2	2	1	1	2	1	4	5	3	4
4	4	4	3	4	5	5	4	5	4
5	4	5	4	4	5	4	4	5	4
5	4	4	5	4	4	4	4	5	4
4	4	5	3	5	5	4	4	4	4
3	2	2	2	2	2	1	2	3	1
3	2	1	2	2	2	1	3	3	2
4	4	4	3	3	4	3	4	4	5
3	4	5	4	4	3	5	5	5	4
5	4	3	4	3	4	3	3	3	4
4	5	4	3	3	5	4	4	4	4
3	4	5	5	3	3	1	2	1	3
4	4	5	4	5	5	5	3	5	4
1	2	2	1	2	3	3	1	3	2
2	2	1	2	3	1	3	1	2	1
4	5	5	4	4	4	4	4	4	4
4	5	3	5	3	3	5	4	5	3
4	4	5	4	4	4	4	5	4	1
5	3	4	4	3	4	4	3	4	4
2	2	2	3	1	2	3	3	2	2
4	4	5	4	3	5	4	5	3	4

4	4	5	5	4	4	4	4	3	4
1	3	2	2	2	2	2	1	2	3
3	5	4	5	4	4	1	1	2	5
5	5	4	3	3	4	4	5	3	3
5	5	3	4	5	4	4	5	4	5
4	4	3	4	3	3	2	2	2	5
4	5	3	4	4	5	4	4	3	4
2	3	2	1	1	2	2	3	3	2
4	4	4	4	3	4	4	4	4	4
5	5	5	4	4	5	4	4	4	5
4	4	4	3	3	3	4	5	5	3
4	3	3	5	4	5	3	4	5	4
5	4	3	4	4	5	4	3	3	5
3	2	2	3	2	2	1	3	2	3
4	4	5	4	3	4	3	4	4	3
3	5	3	4	3	4	3	5	5	5
4	4	5	5	3	5	3	4	5	4
5	5	3	3	3	5	3	5	4	3
4	5	4	3	4	5	4	4	3	5
5	5	4	4	5	3	3	4	3	4
5	5	5	4	3	5	4	5	4	5
2	1	1	2	1	1	4	3	3	3
2	2	2	3	3	1	1	2	3	3
2	2	3	3	3	1	2	2	2	1
5	3	4	5	5	5	5	4	5	5
5	4	4	5	4	4	3	5	4	3
3	3	5	4	4	5	4	5	3	5
4	4	4	3	4	5	4	3	4	3
4	4	4	4	4	4	4	4	3	4
4	5	4	4	4	4	4	3	4	5
3	3	4	4	4	5	5	4	4	4
5	4	3	3	3	4	5	5	3	4
3	5	4	3	5	4	4	4	3	4
2	1	1	2	1	2	3	4	5	3
4	4	5	4	3	4	4	4	4	3
4	4	4	4	4	3	5	4	4	3
5	4	3	3	3	5	3	4	5	5
4	4	4	3	4	3	5	3	4	4
2	2	2	1	1	2	2	3	1	2
3	4	4	4	4	3	4	4	4	5
4	5	4	5	5	4	4	3	4	3
3	5	5	4	4	3	4	5	4	4
4	5	5	4	4	3	4	3	5	5
4	4	5	3	4	4	5	4	4	3
5	5	3	3	4	3	3	5	3	3
2	2	1	2	2	2	5	4	3	4
4	3	4	5	4	4	3	5	4	4

3	4	5	4	4	4	4	5	3	4
4	5	4	5	4	4	4	3	5	3
4	3	5	4	3	4	3	4	4	2
1	1	2	1	2	1	5	5	5	4
2	1	2	2	2	2	1	2	3	2
2	2	2	1	3	3	1	3	1	2
4	4	4	4	3	4	2	2	2	5
2	2	1	2	2	3	1	2	2	1
4	3	5	4	4	4	4	5	5	5
3	4	5	5	4	5	3	4	5	3
3	4	4	3	5	5	3	5	4	5
4	4	4	4	5	4	4	3	3	4
1	2	2	2	1	3	2	2	1	2
3	5	4	5	4	4	3	5	5	1
3	4	5	5	3	4	4	3	5	4
4	3	5	3	4	3	4	4	4	3
4	4	4	4	4	4	5	5	4	3
5	4	3	3	4	4	3	5	4	4
4	5	4	5	3	4	3	5	4	3
5	4	4	3	5	5	3	3	5	4
5	3	3	3	4	4	5	4	4	4
4	4	3	4	4	3	4	4	5	5
5	4	5	3	4	3	4	5	4	3
5	4	4	4	3	5	5	5	4	1
3	5	5	4	4	4	4	3	5	2
5	3	4	4	4	3	3	4	3	3
5	3	3	5	4	5	4	4	3	4
3	4	3	4	4	4	4	3	3	3
5	4	4	4	4	3	5	3	3	4
3	3	4	4	5	4	4	5	3	4
2	3	3	3	1	2	1	3	2	2
4	3	5	4	5	4	4	3	4	5
3	4	5	4	5	5	5	5	4	5
4	4	5	3	4	3	5	4	4	2
1	2	2	2	2	2	2	2	1	3
4	4	4	4	4	5	3	4	4	4
3	2	1	3	2	1	2	1	2	2
4	4	5	4	4	4	4	4	3	4
3	4	3	5	4	5	4	5	3	5
3	4	4	4	5	4	5	5	3	5
4	3	3	4	4	4	4	4	3	4
3	4	4	4	4	3	4	4	3	5
5	4	5	4	3	4	4	3	4	3
1	2	2	1	2	2	5	4	5	3
1	3	1	3	2	3	1	1	2	1
5	3	4	4	3	4	5	3	3	5
4	3	3	4	4	4	4	5	3	4

5	4	3	5	5	4	4	4	5	4
5	5	5	3	3	5	5	4	4	4
3	1	2	3	3	1	2	1	1	2
5	4	5	4	4	3	4	4	3	5
3	4	4	4	5	4	5	4	4	5
3	5	5	3	3	3	3	4	4	5
3	4	4	4	4	3	3	4	4	5
1	2	1	2	1	2	5	4	5	5
4	5	4	3	5	4	5	5	5	5
3	2	1	1	2	2	3	3	3	1
4	4	3	5	4	4	4	5	4	5
4	5	3	4	4	3	4	3	3	5
4	4	4	3	5	4	4	5	4	5
3	4	4	5	4	3	4	4	5	4
5	4	3	5	3	4	5	4	4	3
5	3	4	4	5	4	4	4	4	3
4	4	3	5	5	4	1	2	1	3
4	4	4	4	3	3	4	5	4	4
3	4	4	3	3	4	3	4	3	5
5	4	4	4	4	4	3	4	4	3
4	4	5	4	4	4	3	5	5	4
3	5	4	3	4	3	3	5	3	3
3	4	3	5	4	4	4	5	3	4
5	4	4	4	4	3	5	4	5	4
4	4	4	5	4	4	4	3	4	3
3	5	5	4	4	5	3	4	4	4
5	4	4	4	4	5	3	5	4	3
4	5	3	4	5	4	4	4	4	4
4	5	3	4	4	4	3	4	5	3
4	4	5	4	5	5	4	3	5	5
4	3	5	5	3	4	4	3	3	4
4	3	4	4	4	3	5	4	4	4
2	2	1	1	2	2	3	3	3	1
5	4	3	4	4	5	4	4	5	4
4	3	5	3	5	3	3	5	4	4
3	2	3	2	2	3	3	2	2	2
3	3	4	5	3	4	4	3	3	3
4	5	4	3	4	4	4	4	4	5
2	2	1	3	3	2	3	2	3	1
4	4	4	3	4	5	5	5	3	4
4	4	4	4	5	5	5	5	4	4
5	5	4	5	3	3	4	5	4	5
3	4	3	4	4	4	4	3	3	5
3	3	3	3	5	4	4	4	4	3
3	4	5	5	4	4	4	3	4	4
3	5	4	4	3	4	3	4	3	4
4	4	5	5	4	4	4	5	4	3

4	4	3	4	3	5	3	5	3	4
5	3	4	4	3	4	3	4	5	4
4	4	3	3	4	4	5	4	3	4
2	3	1	2	1	3	2	2	2	2
5	3	5	5	4	3	4	5	5	3
5	4	4	4	5	3	3	4	3	5
3	5	5	4	4	5	5	5	4	3
3	3	3	3	4	4	5	4	5	4
4	5	4	4	3	5	4	4	5	4
4	5	3	3	3	5	4	4	5	4
3	4	3	5	3	4	5	4	3	4
4	3	4	4	5	5	3	4	5	4
4	4	5	3	4	3	3	3	5	4
5	5	4	4	4	5	4	5	4	5
4	4	4	4	3	5	4	3	4	4
4	5	5	3	5	3	5	5	4	5
5	5	3	3	4	5	5	4	4	3
1	2	2	2	2	1	3	2	2	2
5	3	4	4	4	3	4	4	3	4
5	3	4	3	5	4	3	4	3	4
5	3	4	5	4	5	4	4	3	4
4	4	4	4	3	4	4	4	4	4
3	4	5	4	5	4	5	3	4	4
5	5	4	5	5	4	3	4	5	4
5	3	3	5	3	3	4	4	5	4
5	3	5	5	3	3	5	4	5	5
5	5	3	5	4	4	4	4	4	4
4	4	3	3	5	4	4	3	3	3
4	5	5	5	4	5	5	4	3	4
2	2	2	2	3	2	1	3	3	2
4	4	4	5	4	4	3	4	5	5
4	3	4	3	5	4	4	4	3	5
4	4	4	4	5	5	4	4	4	1
3	4	4	5	4	5	5	4	4	5
4	4	3	4	4	5	4	3	4	5
1	1	2	1	2	1	3	4	3	4
5	4	4	4	3	4	4	5	4	4
3	5	3	4	4	3	5	5	5	4
4	4	4	4	3	3	5	3	4	5
1	2	1	1	1	2	3	5	3	4
3	4	3	4	3	5	1	1	1	4
4	4	5	4	4	4	3	5	5	5
4	3	5	3	4	4	4	4	4	5
5	5	4	4	4	3	4	3	4	3
3	3	1	1	3	2	2	3	3	1
4	3	4	4	3	4	5	5	5	5
4	4	5	3	4	4	4	5	4	4

3	4	4	4	3	5	2	2	2	4
4	4	4	4	5	4	4	5	4	4
4	5	4	4	3	4	4	4	4	4
4	3	3	4	3	3	4	5	4	4
3	5	4	4	5	5	3	5	3	3
3	4	5	4	5	3	5	3	4	3
4	4	5	4	5	4	5	4	4	4
3	1	2	3	2	1	3	2	2	3
3	4	4	5	5	5	2	2	2	5
4	3	4	4	4	3	3	4	4	1
4	3	5	4	4	4	5	5	4	5
4	4	4	5	3	5	5	3	3	2
3	2	2	2	2	2	3	2	2	2
1	2	2	2	2	2	3	4	3	3
5	4	3	4	4	4	3	5	3	5
4	4	5	4	4	3	4	3	5	4
5	4	4	5	4	3	5	4	5	5
4	5	4	3	5	4	3	3	4	4
4	5	3	4	4	5	4	3	4	4
5	4	4	4	3	4	5	5	3	3
4	5	5	4	4	3	4	4	5	4
4	3	4	4	3	4	5	3	3	4
4	4	3	3	5	4	4	4	3	5
4	5	4	4	3	3	4	4	4	4
4	4	5	4	3	4	4	4	4	4
4	3	4	4	4	3	4	3	3	5
2	1	2	1	2	2	4	5	5	3
4	4	3	3	5	4	5	3	3	4
4	5	3	3	3	4	3	4	4	5
5	4	4	5	4	4	1	1	2	4
4	4	4	5	4	5	5	4	5	3
2	2	1	2	1	2	1	2	2	2
3	1	1	2	3	2	1	2	2	1
4	4	4	5	3	3	3	4	4	3
5	4	5	4	3	4	4	4	5	5
3	4	3	4	5	3	4	4	4	5
4	5	4	4	5	5	4	3	4	3
3	4	4	5	4	3	5	4	4	4
5	4	3	5	3	4	5	5	4	5
2	1	1	2	2	2	2	3	2	3
4	5	5	5	3	4	5	4	3	3
3	5	4	4	4	3	3	4	5	1
5	4	4	5	4	4	5	4	4	3
1	1	3	3	2	2	2	1	2	2
4	5	4	4	4	5	3	4	3	4
4	5	4	3	4	3	4	5	4	3
3	4	4	4	4	3	3	3	4	4

3	3	4	5	3	4	5	4	5	4
3	4	3	4	4	3	5	5	4	2
3	5	3	4	4	5	4	5	3	4
2	2	1	2	2	1	3	1	2	1
2	3	3	3	2	3	1	1	3	2
2	1	2	2	1	1	3	2	2	2
4	3	3	5	4	5	4	4	3	4
3	5	4	3	5	5	4	3	5	4
4	5	3	3	4	4	3	4	5	3
1	1	2	1	2	1	3	5	3	3
5	5	4	5	5	5	2	1	1	4
3	3	2	2	2	1	1	1	3	2
3	4	5	3	5	3	3	4	4	4
4	5	4	3	4	4	4	4	4	3
3	3	4	5	5	3	4	4	4	3
4	4	5	5	5	4	5	5	4	5
4	4	4	4	4	4	5	3	4	5
4	4	4	4	4	4	1	2	1	5
1	2	3	2	2	3	2	2	2	2
4	5	4	4	4	3	4	5	3	5
4	3	4	5	3	3	3	4	3	5
2	2	1	3	3	2	3	2	2	1
1	1	1	2	1	2	4	4	4	4
1	1	1	2	1	1	5	4	3	4
2	2	2	3	2	2	2	1	2	3
2	1	2	1	1	2	3	3	5	4
3	4	4	4	3	3	5	3	4	4
4	5	5	4	4	4	3	4	5	3
5	4	4	4	3	4	4	4	4	4
5	4	5	4	5	4	5	5	3	4
3	4	3	5	4	5	5	4	4	4
3	5	3	5	4	4	3	4	3	5
5	4	4	4	4	4	4	4	4	5
3	3	3	4	4	4	5	3	3	5
2	1	2	2	3	2	2	2	1	2
4	4	4	4	5	5	5	4	4	4
3	4	5	5	3	4	4	5	3	3
3	5	4	3	4	3	4	4	3	3
4	4	3	5	4	5	3	5	5	4
4	3	4	3	3	4	3	4	3	3
4	4	4	4	4	3	4	5	5	4
4	3	3	4	3	4	4	4	5	4
4	3	3	4	4	5	4	4	5	5
5	5	4	4	5	5	4	3	3	3
3	4	3	4	4	3	5	3	3	4
5	3	3	4	3	4	5	4	3	5
3	5	5	4	5	4	1	1	1	4

3	3	5	4	5	4	2	2	2	5
3	4	4	3	4	4	4	5	5	4
4	5	3	5	4	5	3	4	4	3
4	4	4	3	5	4	4	4	3	4
2	1	1	2	2	1	4	4	3	4
4	5	3	5	4	4	3	4	3	5
4	3	4	4	3	3	4	4	4	4
3	4	5	5	4	4	4	4	4	4
3	4	4	5	4	3	5	4	5	4
5	4	5	5	4	4	5	3	4	4
4	3	4	5	5	4	5	3	4	4
3	4	3	4	4	5	5	4	3	4
4	3	5	5	4	4	4	4	4	4
4	3	5	5	3	4	4	5	4	4
3	5	3	4	5	4	4	5	4	4
2	2	2	2	2	1	2	3	1	3
5	3	4	4	4	4	4	4	4	1
4	3	5	4	4	3	4	4	3	2
5	4	5	3	4	4	5	4	5	4
5	4	4	5	4	5	4	4	4	5
5	5	3	4	5	5	5	3	3	4
2	3	1	2	3	2	1	3	2	2
4	4	4	5	5	4	5	4	4	5
5	5	4	4	3	4	3	5	4	3
3	4	5	3	5	4	5	3	3	3
5	4	4	3	4	4	3	4	4	4
4	5	4	3	5	4	5	4	3	4
4	4	5	4	4	4	1	1	2	4
3	4	4	4	5	5	5	3	4	3
4	5	4	4	5	5	4	3	4	5
1	2	2	2	1	3	2	2	2	3
5	4	3	4	5	3	3	3	4	4
4	4	5	3	4	4	4	4	3	4
2	2	1	3	3	1	2	2	3	2
2	2	1	2	2	2	1	1	3	1
2	1	2	1	2	2	4	3	4	4
4	4	4	5	4	5	4	5	5	3
4	4	4	3	4	3	3	3	4	4
4	5	5	5	4	3	3	5	5	3
4	4	5	5	4	4	3	5	4	5
5	3	4	4	3	3	4	4	3	5
4	4	5	4	4	3	3	4	4	4
4	4	3	5	4	5	5	4	4	4
4	3	5	4	3	3	3	4	4	4
3	3	4	3	4	4	4	5	4	4
3	5	3	4	5	4	4	3	4	4
4	4	4	4	4	4	3	5	3	4

4	5	4	3	4	5	3	5	5	5
4	4	4	3	4	3	4	5	5	3
4	4	4	5	4	3	4	3	4	4
1	1	1	2	2	2	4	3	3	4
4	4	5	3	4	4	5	5	5	2
3	2	1	3	1	1	2	2	3	1
3	4	3	4	5	3	4	4	4	4
5	3	3	3	4	4	5	4	5	4
5	4	4	4	5	4	3	4	3	3
3	4	4	3	4	3	5	4	5	1
3	4	4	5	4	5	4	4	4	3
4	4	4	3	5	3	4	4	3	3
3	3	5	4	3	4	3	3	4	4
4	4	5	5	4	4	5	5	4	5
4	5	3	5	3	3	4	4	4	3
3	5	5	5	4	4	4	4	5	5
1	1	2	2	3	2	2	2	1	1
4	5	3	3	5	3	4	5	4	4
5	4	3	4	4	4	4	5	4	4
4	5	4	4	3	4	5	4	5	3
4	4	3	4	3	3	2	1	1	4
4	4	5	4	4	5	5	4	5	4
5	3	3	4	5	5	5	3	5	4
2	3	1	1	1	2	3	2	3	2
2	3	3	2	2	2	2	2	3	3
1	2	2	1	2	2	2	1	2	1
4	3	3	4	4	5	4	5	3	5
3	3	4	5	4	4	5	5	5	4
3	3	3	4	3	3	4	4	3	3
4	3	4	4	5	4	4	3	3	3
4	5	4	4	4	4	4	4	3	4
1	2	1	2	1	2	4	4	4	4
5	4	4	5	5	4	4	5	3	5
4	4	4	4	4	4	3	4	4	4
4	5	4	4	3	4	4	4	4	5
4	4	4	4	3	5	4	5	4	3
3	3	4	3	4	4	4	3	3	4
4	4	3	5	3	5	3	4	5	3
5	4	3	4	5	3	3	5	4	4
4	4	5	5	4	3	4	5	4	4
4	5	4	5	4	4	4	3	4	4
3	4	4	4	4	4	4	4	4	4
5	4	4	3	5	4	5	3	3	3
4	3	3	4	5	4	5	4	4	4
1	1	3	2	3	2	3	2	1	1
4	4	3	5	4	3	4	4	5	3
3	1	2	2	3	2	1	2	3	2

4	4	4	5	5	5	4	4	5	4
4	3	4	5	3	4	4	4	4	5
5	3	5	3	4	4	4	4	3	5
3	5	5	4	4	4	4	4	4	4
4	5	4	4	4	3	4	4	3	1
1	3	2	2	3	2	2	1	2	2
4	5	5	4	3	5	3	4	3	4
5	5	3	5	5	4	4	5	4	1
5	3	5	3	4	4	2	1	1	4
5	3	4	4	4	4	4	4	4	4
4	4	3	5	3	4	5	4	4	4
2	2	2	2	2	2	1	3	1	3
2	2	2	2	2	2	2	2	3	1
4	4	3	4	4	3	2	2	1	5
4	3	5	4	4	4	4	5	4	5
4	4	5	5	5	3	5	3	4	5
2	2	1	2	1	1	5	4	5	4
5	4	4	3	3	4	4	4	3	5
5	4	4	3	5	4	4	4	4	3
3	4	4	3	4	3	5	3	4	4
5	4	4	4	4	3	4	4	3	5
5	4	4	4	4	5	3	4	3	5
5	5	3	5	5	3	4	4	4	4
3	4	5	3	4	4	4	5	5	4
4	3	4	5	4	3	5	5	4	3
4	5	4	5	4	4	4	3	3	5
4	4	5	3	3	5	4	3	4	4
4	5	3	3	3	4	4	4	3	4
2	1	2	2	3	1	2	1	2	1
4	4	3	3	3	4	3	5	3	4
2	1	2	3	2	2	1	2	3	3
4	3	4	4	3	3	4	4	4	4
3	5	3	4	3	3	3	5	5	5
4	4	5	4	4	3	4	4	5	3
3	4	3	4	3	4	3	5	4	4
4	5	5	4	3	3	3	4	3	4
1	2	1	2	1	3	2	2	3	3
5	5	4	5	3	4	5	3	5	4
4	4	3	4	4	5	3	4	4	4
2	3	3	3	2	2	2	2	2	2
3	3	3	4	5	4	4	4	3	4
3	4	4	5	3	4	4	3	5	4
4	5	5	4	5	5	3	5	4	4
5	4	4	4	4	3	5	5	3	5
5	4	4	3	3	4	4	4	5	4
4	5	4	4	4	5	4	4	5	2
4	4	4	3	5	4	5	4	5	4

4	4	4	4	3	5	5	4	5	3
4	4	5	4	4	5	4	4	4	3
3	4	4	3	4	4	4	3	4	4
4	4	4	3	3	3	4	3	4	3
3	4	3	5	3	5	1	2	1	4
3	2	2	3	2	2	2	1	2	3
4	5	5	4	5	5	3	3	5	5
4	3	5	4	3	4	4	3	4	5
3	4	4	4	5	4	4	3	5	3
5	4	4	5	3	5	4	4	3	5
3	5	4	4	3	3	3	3	5	5
4	4	4	4	4	4	3	3	4	4
5	5	5	5	5	4	5	4	5	3
3	4	5	4	4	5	5	3	4	4
3	4	3	4	5	4	4	4	3	4
3	4	4	4	4	4	4	5	5	4
4	5	5	4	5	5	4	3	5	5
2	2	1	1	2	1	4	5	3	4
4	4	4	3	4	5	5	4	5	4
5	4	5	4	4	5	4	4	5	4
5	4	4	5	4	4	4	4	5	4
4	4	5	3	5	5	4	4	4	4
3	2	2	2	2	2	1	2	3	1
3	2	1	2	2	2	1	3	3	2
4	4	4	3	3	4	3	4	4	5
3	4	5	4	4	3	5	5	5	4
5	4	3	4	3	4	3	3	3	4
4	5	4	3	3	5	4	4	4	4
3	4	5	5	3	3	1	2	1	3
4	4	5	4	5	5	5	3	5	4
1	2	2	1	2	3	3	1	3	2
2	2	1	2	3	1	3	1	2	1
4	5	5	4	4	4	4	4	4	4
4	5	3	5	3	3	5	4	5	3
4	4	5	4	4	4	4	5	4	1
5	3	4	4	3	4	4	3	4	4
2	2	2	3	1	2	3	3	2	2
4	4	5	4	3	5	4	5	3	4
4	4	5	5	4	4	4	4	3	4
1	3	2	2	2	2	2	1	2	3
3	5	4	5	4	4	1	1	2	5
5	5	4	3	3	4	4	5	3	3
5	5	3	4	5	4	4	5	4	5
4	4	3	4	3	3	2	2	2	5
4	5	3	4	4	5	4	4	3	4
2	3	2	1	1	2	2	3	3	2
4	4	4	4	3	4	4	4	4	4

5	5	5	4	4	5	4	4	4	5
4	4	4	3	3	3	4	5	5	3
4	3	3	5	4	5	3	4	5	4
5	4	3	4	4	5	4	3	3	5
3	2	2	3	2	2	1	3	2	3
4	4	5	4	3	4	3	4	4	3
3	5	3	4	3	4	3	5	5	5
4	4	5	5	3	5	3	4	5	4
5	5	3	3	3	5	3	5	4	3
4	5	4	3	4	5	4	4	3	5
5	5	4	4	5	3	3	4	3	4
5	5	5	4	3	5	4	5	4	5
2	1	1	2	1	1	4	3	3	3
2	2	2	3	3	1	1	2	3	3
2	2	3	3	3	1	2	2	2	1
5	3	4	5	5	5	5	4	5	5
5	4	4	5	4	4	3	5	4	3
3	3	5	4	4	5	4	5	3	5
4	4	4	3	4	5	4	3	4	3
4	4	4	4	4	4	4	4	3	4
4	5	4	4	4	4	4	3	4	5
3	3	4	4	4	5	5	4	4	4
5	4	3	3	3	4	5	5	3	4
3	5	4	3	5	4	4	4	3	4
2	1	1	2	1	2	3	4	5	3
4	4	5	4	3	4	4	4	4	3
4	4	4	4	4	3	5	4	4	3
5	4	3	3	3	5	3	4	5	5
4	4	4	3	4	3	5	3	4	4
2	2	2	1	1	2	2	3	1	2
3	4	4	4	4	3	4	4	4	5
4	5	4	5	5	4	4	3	4	3
3	5	5	4	4	3	4	5	4	4
4	5	5	4	4	3	4	3	5	5
4	4	5	3	4	4	5	4	4	3
5	5	3	3	4	3	3	5	3	3
2	2	1	2	2	2	5	4	3	4
4	3	4	5	4	4	3	5	4	4
3	4	5	4	4	4	4	5	3	4
4	5	4	5	4	4	4	3	5	3
4	3	5	4	3	4	3	4	4	2
1	1	2	1	2	1	5	5	5	4
2	1	2	2	2	2	1	2	3	2
2	2	2	1	3	3	1	3	1	2
4	4	4	4	3	4	2	2	2	5
2	2	1	2	2	3	1	2	2	1
4	3	5	4	4	4	4	5	5	5

3	4	5	5	4	5	3	4	5	3
3	4	4	3	5	5	3	5	4	5
4	4	4	4	5	4	4	3	3	4
1	2	2	2	1	3	2	2	1	2
3	5	4	5	4	4	3	5	5	1
3	4	5	5	3	4	4	3	5	4
4	3	5	3	4	3	4	4	4	3
4	4	4	4	4	4	5	5	4	3
5	4	3	3	4	4	3	5	4	4
4	5	4	5	3	4	3	5	4	3
5	4	4	3	5	5	3	3	5	4
5	3	3	3	4	4	5	4	4	4
4	4	3	4	4	3	4	4	5	5
5	4	5	3	4	3	4	5	4	3
5	4	4	4	3	5	5	5	4	1
3	5	5	4	4	4	4	3	5	2
5	3	4	4	4	3	3	4	3	3
5	3	3	5	4	5	4	4	3	4
3	4	3	4	4	4	4	3	3	3
5	4	4	4	4	3	5	3	3	4
3	3	4	4	5	4	4	5	3	4
2	3	3	3	1	2	1	3	2	2
4	3	5	4	5	4	4	3	4	5
3	4	5	4	5	5	5	5	4	5
4	4	5	3	4	3	5	4	4	2
1	2	2	2	2	2	2	2	1	3
4	4	4	4	4	5	3	4	4	4
3	2	1	3	2	1	2	1	2	2
4	4	5	4	4	4	4	4	3	4
3	4	3	5	4	5	4	5	3	5
3	4	4	4	5	4	5	5	3	5
4	3	3	4	4	4	4	4	3	4
3	4	4	4	4	3	4	4	3	5
5	4	5	4	3	4	4	3	4	3
1	2	2	1	2	2	5	4	5	3
1	3	1	3	2	3	1	1	2	1
5	3	4	4	3	4	5	3	3	5
4	3	3	4	4	4	4	5	3	4
5	4	3	5	5	4	4	4	5	4
5	5	5	3	3	5	5	4	4	4
3	1	2	3	3	1	2	1	1	2
5	4	5	4	4	3	4	4	3	5
3	4	4	4	5	4	5	4	4	5
3	5	5	3	3	3	3	4	4	5
3	4	4	4	4	3	3	4	4	5
1	2	1	2	1	2	5	4	5	5
4	5	4	3	5	4	5	5	5	5

3	2	1	1	2	2	3	3	3	1
4	4	3	5	4	4	4	5	4	5
4	5	3	4	4	3	4	3	3	5
4	4	4	3	5	4	4	5	4	5
3	4	4	5	4	3	4	4	5	4
5	4	3	5	3	4	5	4	4	3
5	3	4	4	5	4	4	4	4	3
4	4	3	5	5	4	1	2	1	3
4	4	4	4	3	3	4	5	4	4
3	4	4	3	3	4	3	4	3	5
5	4	4	4	4	4	3	4	4	3
4	4	5	4	4	4	3	5	5	4
3	5	4	3	4	3	3	5	3	3
3	4	3	5	4	4	4	5	3	4
5	4	4	4	4	3	5	4	5	4
4	4	4	5	4	4	4	3	4	3
3	5	5	4	4	5	3	4	4	4
5	4	4	4	4	5	3	5	4	3
4	5	3	4	5	4	4	4	4	4
4	5	3	4	4	4	3	4	5	3
4	4	5	4	5	5	4	3	5	5
4	3	5	5	3	4	4	3	3	4
4	3	4	4	4	3	5	4	4	4
2	2	1	1	2	2	3	3	3	1
5	4	3	4	4	5	4	4	5	4
4	3	5	3	5	3	3	5	4	4
3	2	3	2	2	3	3	2	2	2
3	3	4	5	3	4	4	3	3	3
4	5	4	3	4	4	4	4	4	5
2	2	1	3	3	2	3	2	3	1
4	4	4	3	4	5	5	5	3	4
4	4	4	4	5	5	5	5	4	4
5	5	4	5	3	3	4	5	4	5
3	4	3	4	4	4	4	3	3	5
3	3	3	3	5	4	4	4	4	3
3	4	5	5	4	4	4	3	4	4
3	5	4	4	3	4	3	4	3	4
4	4	5	5	4	4	4	5	4	3
4	4	3	4	3	5	3	5	3	4
5	3	4	4	3	4	3	4	5	4
4	4	3	3	4	4	5	4	3	4
2	3	1	2	1	3	2	2	2	2
5	3	5	5	4	3	4	5	5	3
5	4	4	4	5	3	3	4	3	5
3	5	5	4	4	5	5	5	4	3
3	3	3	3	4	4	5	4	5	4
4	5	4	4	3	5	4	4	5	4

4	5	3	3	3	5	4	4	5	4
3	4	3	5	3	4	5	4	3	4
4	3	4	4	5	5	3	4	5	4
4	4	5	3	4	3	3	3	5	4
5	5	4	4	4	5	4	5	4	5
4	4	4	4	3	5	4	3	4	4
4	5	5	3	5	3	5	5	4	5
5	5	3	3	4	5	5	4	4	3
1	2	2	2	2	1	3	2	2	2
5	3	4	4	4	3	4	4	3	4
5	3	4	3	5	4	3	4	3	4
5	3	4	5	4	5	4	4	3	4
4	4	4	4	3	4	4	4	4	4
3	4	5	4	5	4	5	3	4	4
5	5	4	5	5	4	3	4	5	4
5	3	3	5	3	3	4	4	5	4
5	3	5	5	3	3	5	4	5	5
5	5	3	5	4	4	4	4	4	4
4	4	3	3	5	4	4	3	3	3
4	5	5	5	4	5	5	4	3	4
2	2	2	2	3	2	1	3	3	2
4	4	4	5	4	4	3	4	5	5
4	3	4	3	5	4	4	4	3	5
4	4	4	4	5	5	4	4	4	1
3	4	4	5	4	5	5	4	4	5
4	4	3	4	4	5	4	3	4	5
1	1	2	1	2	1	3	4	3	4
5	4	4	4	3	4	4	5	4	4
3	5	3	4	4	3	5	5	5	4
4	4	4	4	3	3	5	3	4	5
1	2	1	1	1	2	3	5	3	4
3	4	3	4	3	5	1	1	1	4
4	4	5	4	4	4	3	5	5	5
4	3	5	3	4	4	4	4	4	5
5	5	4	4	4	3	4	3	4	3
3	3	1	1	3	2	2	3	3	1
4	3	4	4	3	4	5	5	5	5
4	4	5	3	4	4	4	5	4	4
3	4	4	4	3	5	2	2	2	4
4	4	4	4	5	4	4	5	4	4
4	5	4	4	3	4	4	4	4	4
4	3	3	4	3	3	4	5	4	4
3	5	4	4	5	5	3	5	3	3
3	4	5	4	5	3	5	3	4	3
4	4	5	4	5	4	5	4	4	4
3	1	2	3	2	1	3	2	2	3
3	4	4	5	5	5	2	2	2	5

4	3	4	4	4	3	3	4	4	1
4	3	5	4	4	4	5	5	4	5
4	4	4	5	3	5	5	3	3	2
3	2	2	2	2	2	3	2	2	2
1	2	2	2	2	2	3	4	3	3
5	4	3	4	4	4	3	5	3	5
4	4	5	4	4	3	4	3	5	4
5	4	4	5	4	3	5	4	5	5
4	5	4	3	5	4	3	3	4	4
4	5	3	4	4	5	4	3	4	4
5	4	4	4	3	4	5	5	3	3
4	5	5	4	4	3	4	4	5	4
4	3	4	4	3	4	5	3	3	4
4	4	3	3	5	4	4	4	3	5
4	5	4	4	3	3	4	4	4	4
4	4	5	4	3	4	4	4	4	4
4	3	4	4	4	3	4	3	3	5
2	1	2	1	2	2	4	5	5	3
4	4	3	3	5	4	5	3	3	4
4	5	3	3	3	4	3	4	4	5
5	4	4	5	4	4	1	1	2	4
4	4	4	5	4	5	5	4	5	3
2	2	1	2	1	2	1	2	2	2
3	1	1	2	3	2	1	2	2	1
4	4	4	5	3	3	3	4	4	3
5	4	5	4	3	4	4	4	5	5
3	4	3	4	5	3	4	4	4	5
4	5	4	4	5	5	4	3	4	3
3	4	4	5	4	3	5	4	4	4
5	4	3	5	3	4	5	5	4	5
2	1	1	2	2	2	2	3	2	3
4	5	5	5	3	4	5	4	3	3
3	5	4	4	4	3	3	4	5	1
5	4	4	5	4	4	5	4	4	3
1	1	3	3	2	2	2	1	2	2
4	5	4	4	4	5	3	4	3	4
4	5	4	3	4	3	4	5	4	3
3	4	4	4	4	3	3	3	4	4
3	3	4	5	3	4	5	4	5	4
3	4	3	4	4	3	5	5	4	2
3	5	3	4	4	5	4	5	3	4
2	2	1	2	2	1	3	1	2	1
2	3	3	3	2	3	1	1	3	2
2	1	2	2	1	1	3	2	2	2
4	3	3	5	4	5	4	4	3	4
3	5	4	3	5	5	4	3	5	4
4	5	3	3	4	4	3	4	5	3

1	1	2	1	2	1	3	5	3	3
5	5	4	5	5	5	2	1	1	4
3	3	2	2	2	1	1	1	3	2
3	4	5	3	5	3	3	4	4	4
4	5	4	3	4	4	4	4	4	3
3	3	4	5	5	3	4	4	4	3
4	4	5	5	5	4	5	5	4	5
4	4	4	4	4	4	5	3	4	5
4	4	4	4	4	4	1	2	1	5
1	2	3	2	2	3	2	2	2	2
4	5	4	4	4	3	4	5	3	5
4	3	4	5	3	3	3	4	3	5
2	2	1	3	3	2	3	2	2	1
1	1	1	2	1	2	4	4	4	4
1	1	1	2	1	1	5	4	3	4
2	2	2	3	2	2	2	1	2	3
2	1	2	1	1	2	3	3	5	4
3	4	4	4	3	3	5	3	4	4
4	5	5	4	4	4	3	4	5	3
5	4	4	4	3	4	4	4	4	4
5	4	5	4	5	4	5	5	3	4
3	4	3	5	4	5	5	4	4	4
3	5	3	5	4	4	3	4	3	5
5	4	4	4	4	4	4	4	4	5
3	3	3	4	4	4	5	3	3	5
2	1	2	2	3	2	2	2	1	2
4	4	4	4	5	5	5	4	4	4
3	4	5	5	3	4	4	5	3	3
3	5	4	3	4	3	4	4	3	3
4	4	3	5	4	5	3	5	5	4
4	3	4	3	3	4	3	4	3	3
4	4	4	4	4	3	4	5	5	4
4	3	3	4	3	4	4	4	5	4
4	3	3	4	4	5	4	4	5	5
5	5	4	4	5	5	4	3	3	3
3	4	3	4	4	3	5	3	3	4
5	3	3	4	3	4	5	4	3	5
3	5	5	4	5	4	1	1	1	4
3	3	5	4	5	4	2	2	2	5
3	4	4	3	4	4	4	5	5	4
4	5	3	5	4	5	3	4	4	3
4	4	4	3	5	4	4	4	3	4
2	1	1	2	2	1	4	4	3	4
4	5	3	5	4	4	3	4	3	5
4	3	4	4	3	3	4	4	4	4
3	4	5	5	4	4	4	4	4	4
3	4	4	5	4	3	5	4	5	4

5	4	5	5	4	4	5	3	4	4
4	3	4	5	5	4	5	3	4	4
3	4	3	4	4	5	5	4	3	4
4	3	5	5	4	4	4	4	4	4
4	3	5	5	3	4	4	5	4	4
3	5	3	4	5	4	4	5	4	4
2	2	2	2	2	1	2	3	1	3
5	3	4	4	4	4	4	4	4	1
4	3	5	4	4	3	4	4	3	2
5	4	5	3	4	4	5	4	5	4
5	4	4	5	4	5	4	4	4	5
5	5	3	4	5	5	5	3	3	4
2	3	1	2	3	2	1	3	2	2
4	4	4	5	5	4	5	4	4	5
5	5	4	4	3	4	3	5	4	3
3	4	5	3	5	4	5	3	3	3
5	4	4	3	4	4	3	4	4	4
4	5	4	3	5	4	5	4	3	4
4	4	5	4	4	4	1	1	2	4
3	4	4	4	5	5	5	3	4	3
4	5	4	4	5	5	4	3	4	5
1	2	2	2	1	3	2	2	2	3
5	4	3	4	5	3	3	3	4	4
4	4	5	3	4	4	4	4	3	4
2	2	1	3	3	1	2	2	3	2
2	2	1	2	2	2	1	1	3	1
2	1	2	1	2	2	4	3	4	4
4	4	4	5	4	5	4	5	5	3
4	4	4	3	4	3	3	3	4	4
4	5	5	5	4	3	3	5	5	3
4	4	5	5	4	4	3	5	4	5
5	3	4	4	3	3	4	4	3	5
4	4	5	4	4	3	3	4	4	4
4	4	3	5	4	5	5	4	4	4
4	3	5	4	3	3	3	4	4	4
3	3	4	3	4	4	4	5	4	4
3	5	3	4	5	4	4	3	4	4
4	4	4	4	4	4	3	5	3	4
4	5	4	3	4	5	3	5	5	5
4	4	4	3	4	3	4	5	5	3
4	4	4	5	4	3	4	3	4	4
1	1	1	2	2	2	4	3	3	4
4	4	5	3	4	4	5	5	5	2
3	2	1	3	1	1	2	2	3	1
3	4	3	4	5	3	4	4	4	4
5	3	3	3	4	4	5	4	5	4
5	4	4	4	5	4	3	4	3	3

3	4	4	3	4	3	5	4	5	1
3	4	4	5	4	5	4	4	4	3
4	4	4	3	5	3	4	4	3	3
3	3	5	4	3	4	3	3	4	4
4	4	5	5	4	4	5	5	4	5
4	5	3	5	3	3	4	4	4	3
3	5	5	5	4	4	4	4	5	5
1	1	2	2	3	2	2	2	1	1
4	5	3	3	5	3	4	5	4	4
5	4	3	4	4	4	4	5	4	4
4	5	4	4	3	4	5	4	5	3
4	4	3	4	3	3	2	1	1	4
4	4	5	4	4	5	5	4	5	4
5	3	3	4	5	5	5	3	5	4
2	3	1	1	1	2	3	2	3	2
2	3	3	2	2	2	2	2	3	3
1	2	2	1	2	2	2	1	2	1
4	3	3	4	4	5	4	5	3	5
3	3	4	5	4	4	5	5	5	4
3	3	3	4	3	3	4	4	3	3
4	3	4	4	5	4	4	3	3	3
4	4	4	3	4	3	4	5	5	3
4	4	4	5	4	3	4	3	4	4
1	1	1	2	2	2	4	3	3	4
4	4	5	3	4	4	5	5	5	2
3	2	1	3	1	1	2	2	3	1
3	4	3	4	5	3	4	4	4	4
5	3	3	3	4	4	5	4	5	4
5	4	4	4	5	4	3	4	3	3
3	4	4	3	4	3	5	4	5	1
3	4	4	5	4	5	4	4	4	3
4	4	4	3	5	3	4	4	3	3
3	3	5	4	3	4	3	3	4	4
4	4	5	5	4	4	5	5	4	5
4	5	3	5	3	3	4	4	4	3
3	5	5	5	4	4	4	4	5	5
1	1	2	2	3	2	2	2	1	1
4	5	3	3	5	3	4	5	4	4
5	4	3	4	4	4	4	5	4	4
4	5	4	4	3	4	5	4	5	3
4	4	3	4	3	3	2	1	1	4
4	4	5	4	4	5	5	4	5	4
5	3	3	4	5	5	5	3	5	4
2	3	1	1	1	2	3	2	3	2
2	3	3	2	2	2	2	2	3	3
1	2	2	1	2	2	2	1	2	1
4	3	3	4	4	5	4	5	3	5

3	3	4	5	4	4	5	5	5	4
3	3	3	4	3	3	4	4	3	3
4	3	4	4	5	4	4	3	3	3
4	4	4	3	4	3	4	5	5	3
4	4	4	5	4	3	4	3	4	4
1	1	1	2	2	2	4	3	3	4
4	4	5	3	4	4	5	5	5	2
3	2	1	3	1	1	2	2	3	1
3	4	3	4	5	3	4	4	4	4
5	3	3	3	4	4	5	4	5	4
5	4	4	4	5	4	3	4	3	3
3	4	4	3	4	3	5	4	5	1
3	4	4	5	4	5	4	4	4	3
4	4	4	3	5	3	4	4	3	3
3	3	5	4	3	4	3	3	4	4
4	4	5	5	4	4	5	5	4	5
4	5	3	5	3	3	4	4	4	3
3	5	5	5	4	4	4	4	5	5
1	1	2	2	3	2	2	2	1	1
4	5	3	3	5	3	4	5	4	4
5	4	3	4	4	4	4	5	4	4
4	5	4	4	3	4	5	4	5	3
4	4	3	4	3	3	2	1	1	4
4	4	5	4	4	5	5	4	5	4
5	3	3	4	5	5	5	3	5	4
2	3	1	1	1	2	3	2	3	2
2	3	3	2	2	2	2	2	3	3
1	2	2	1	2	2	2	1	2	1
4	3	3	4	4	5	4	5	3	5
3	3	4	5	4	4	5	5	5	4
3	3	3	4	3	3	4	4	3	3
4	3	4	4	5	4	4	3	3	3
4	4	4	3	4	3	4	5	5	3
4	4	4	5	4	3	4	3	4	4
1	1	1	2	2	2	4	3	3	4
4	4	5	3	4	4	5	5	5	2
3	2	1	3	1	1	2	2	3	1
3	4	3	4	5	3	4	4	4	4
5	3	3	3	4	4	5	4	5	4
5	4	4	4	5	4	3	4	3	3
3	4	4	3	4	3	5	4	5	1
3	4	4	5	4	5	4	4	4	3
4	4	4	3	5	3	4	4	3	3
3	3	5	4	3	4	3	3	4	4
4	4	5	5	4	4	5	5	4	5
4	5	3	5	3	3	4	4	4	3
3	5	5	5	4	4	4	4	5	5

1	1	2	2	3	2	2	2	1	1
4	5	3	3	5	3	4	5	4	4
5	4	3	4	4	4	4	5	4	4
4	5	4	4	3	4	5	4	5	3
4	4	3	4	3	3	2	1	1	4
4	4	5	4	4	5	5	4	5	4
5	3	3	4	5	5	5	3	5	4
2	3	1	1	1	2	3	2	3	2
2	3	3	2	2	2	2	2	3	3
1	2	2	1	2	2	2	1	2	1
4	3	3	4	4	5	4	5	3	5
3	3	4	5	4	4	5	5	5	4
3	3	3	4	3	3	4	4	3	3
4	3	4	4	5	4	4	3	3	3
2	3	1	1	1	2	3	2	3	2
2	3	3	2	2	2	2	2	3	3
1	2	2	1	2	2	2	1	2	1
4	3	3	4	4	5	4	5	3	5
3	3	4	5	4	4	5	5	5	4
3	3	3	4	3	3	4	4	3	3
4	3	4	4	5	4	4	3	3	3
2	3	1	1	1	2	3	2	3	2
2	3	3	2	2	2	2	2	3	3
1	2	2	1	2	2	2	1	2	1
4	3	3	4	4	5	4	5	3	5
1	2	2	1	2	2	2	1	2	1

IAS_2	IAS_3	IAS_4	IAS_5	IAS_6	IAS_7	IAS_8	IAS_9	IAS_10	IAS_11
5	5	3	4	3	5	4	5	4	4
4	4	4	3	4	3	1	2	1	2
5	3	3	4	3	4	5	4	4	5
5	5	4	5	4	5	4	4	4	4
5	5	4	3	4	4	4	5	4	4
4	4	5	3	4	4	4	4	4	4
3	4	4	3	3	3	3	3	4	3
3	4	4	5	5	3	4	4	3	5
5	4	5	5	4	3	5	4	3	4
4	3	4	5	4	4	4	4	5	5
5	5	4	4	4	5	4	5	4	5
5	4	4	4	4	4	3	4	4	4
3	4	4	4	3	3	5	4	4	3
5	4	4	5	4	5	4	3	3	4
2	3	3	1	1	3	1	1	3	2
5	4	3	4	5	4	4	4	3	5
3	2	1	3	3	1	3	1	2	2
4	5	3	3	5	4	4	4	4	5
3	5	4	4	4	4	4	3	4	5
5	4	4	4	3	4	5	3	5	3
4	4	4	5	4	5	3	5	5	4
1	2	2	1	3	3	4	5	4	4
2	3	3	2	2	1	1	3	2	2
3	4	3	4	3	3	4	5	5	4
2	2	1	1	4	4	5	5	3	5
4	4	5	3	1	2	5	3	5	3
4	3	4	5	4	4	5	3	4	4
3	4	5	4	4	5	4	4	3	5
3	1	2	1	1	2	2	2	2	2
3	1	3	1	3	2	2	2	2	2
4	4	4	5	1	1	4	4	3	4
3	4	4	5	4	4	4	3	5	4
4	3	4	5	4	4	4	4	5	5
4	4	5	4	5	5	2	2	1	2
4	4	3	5	3	3	5	4	4	3
3	4	5	4	4	5	5	4	4	3
3	4	4	4	4	3	3	4	4	3
3	4	5	4	3	4	5	4	4	4
4	5	5	3	3	4	5	4	4	4
4	4	5	4	4	4	5	5	3	5
5	3	4	5	5	3	3	4	5	3
5	5	4	3	4	4	4	3	4	5
3	5	5	3	3	4	4	5	4	5
3	5	4	4	4	4	4	4	5	3
3	5	3	5	3	3	4	5	3	3
3	1	2	2	2	1	2	1	2	2

4	4	4	4	3	4	4	4	3	3
1	3	2	2	3	1	2	1	2	3
4	3	4	4	4	4	4	3	4	4
4	5	3	4	5	5	3	5	3	4
4	5	4	4	5	3	4	4	5	4
4	5	4	5	4	5	3	4	3	4
4	4	5	5	3	4	4	5	5	4
3	1	2	3	3	1	1	2	1	2
5	5	5	4	5	5	5	5	4	5
4	4	4	4	4	3	4	4	3	4
2	2	3	3	2	2	2	3	3	3
4	3	3	3	3	4	3	3	3	4
4	4	5	3	5	3	3	4	4	5
4	4	5	4	4	4	4	5	5	4
5	3	3	5	3	4	5	4	4	4
3	4	3	3	5	4	5	4	4	3
2	2	2	2	5	5	4	5	4	4
4	4	4	5	5	4	4	4	4	3
4	5	4	5	5	4	4	4	4	4
4	4	4	4	4	3	4	4	5	4
5	3	4	4	4	4	3	4	4	3
4	4	4	4	4	4	4	4	4	3
5	3	4	3	1	2	3	4	3	5
3	2	2	3	2	2	3	2	2	3
3	4	4	4	5	5	4	5	5	4
3	3	4	4	4	4	4	3	5	4
5	3	4	5	5	3	3	4	4	4
4	3	4	4	3	4	5	4	4	5
5	3	3	4	5	4	3	5	4	4
5	4	3	3	4	5	4	4	4	4
3	3	5	5	5	4	5	5	5	5
3	3	5	4	4	4	3	4	5	4
5	4	4	4	3	5	3	4	3	4
4	5	5	3	5	4	3	4	4	4
4	5	3	3	5	3	4	5	5	4
4	4	4	5	3	4	2	2	1	1
4	4	4	4	5	3	4	4	4	3
3	4	5	3	5	4	5	4	5	4
4	5	4	4	5	5	5	4	4	5
4	4	5	4	4	3	4	4	5	3
2	2	2	2	3	2	3	2	2	2
2	1	1	1	3	2	3	2	1	2
4	5	4	4	4	4	4	4	4	3
4	5	3	5	5	5	3	4	5	4
5	4	4	3	3	4	5	4	3	4
3	5	4	5	4	5	4	5	4	3
4	4	4	5	1	2	3	4	5	5

4	4	3	5	5	5	4	4	5	4
2	1	1	3	3	2	1	2	2	1
2	2	1	2	2	1	2	2	1	2
4	3	4	5	4	3	4	5	5	4
3	5	5	4	5	4	4	5	3	5
1	2	1	2	4	4	4	4	5	4
5	3	4	4	4	3	5	3	4	4
2	1	2	2	2	2	2	2	2	3
4	4	4	3	3	3	4	4	5	4
3	5	4	4	3	5	4	4	5	5
1	1	2	2	2	1	1	3	2	2
4	4	3	3	2	1	3	5	4	5
5	5	3	4	3	3	5	5	4	3
3	4	4	5	4	4	5	5	3	4
4	5	5	5	2	2	4	4	3	4
5	5	4	3	3	4	4	5	3	4
3	2	2	2	3	3	2	3	2	1
4	4	5	3	4	4	4	4	4	4
4	3	5	3	4	4	5	5	5	4
4	4	3	4	5	4	4	4	4	3
5	3	5	4	5	5	4	3	3	5
3	3	4	4	3	3	5	4	3	4
2	2	1	2	2	3	3	2	2	3
3	4	4	3	4	4	4	4	5	4
4	4	5	3	5	4	3	5	3	4
5	4	5	4	5	3	4	4	5	5
5	5	5	4	4	5	5	5	3	3
4	5	4	3	3	5	4	5	4	3
4	4	4	5	3	5	5	5	4	4
4	5	4	4	4	3	5	5	5	4
5	4	5	4	3	3	2	1	1	2
2	3	2	1	3	1	2	2	2	3
1	2	3	2	2	1	2	2	3	3
3	5	5	5	5	3	5	3	4	5
5	4	4	5	4	5	5	4	4	5
5	4	4	4	3	3	3	3	5	4
4	4	5	4	4	3	4	4	4	3
4	4	4	3	3	3	4	4	4	4
3	4	4	5	4	4	4	5	4	4
4	4	5	3	4	3	3	3	4	4
3	4	5	4	3	5	5	4	3	3
5	4	4	4	3	3	3	5	4	3
3	4	4	3	5	4	2	1	1	2
4	4	3	4	4	4	4	4	5	4
4	5	5	4	4	3	4	4	4	4
4	4	4	5	5	3	5	4	3	3
4	5	4	5	4	5	4	4	4	3

1	2	2	2	1	2	2	2	2	1
5	5	3	4	4	3	3	4	4	4
4	5	3	4	4	4	4	5	4	5
4	3	3	4	4	4	3	5	5	4
4	5	4	5	5	3	4	5	5	4
5	4	5	4	4	3	4	4	5	3
4	4	5	4	3	4	5	5	3	3
4	3	4	4	3	4	2	2	1	2
5	4	3	3	4	4	4	3	4	5
4	3	4	5	3	3	3	4	5	4
3	4	3	3	5	3	4	5	4	5
2	2	2	2	4	4	4	3	5	4
3	3	4	5	5	3	1	1	2	1
3	3	2	1	3	3	2	1	2	2
1	2	2	2	1	1	2	2	2	1
3	5	4	4	2	1	4	4	4	4
1	2	2	3	2	2	2	2	1	2
4	4	4	5	5	5	4	3	5	4
5	3	4	4	5	3	3	4	5	5
4	4	4	3	4	5	3	4	4	3
3	4	5	4	3	3	4	4	4	4
3	2	2	3	1	3	1	2	2	2
1	1	2	1	5	3	3	5	4	5
4	4	4	3	5	4	3	4	5	5
3	3	3	3	4	3	4	3	5	3
3	3	5	5	4	4	4	4	4	4
5	4	4	3	4	4	5	4	3	3
5	5	4	4	4	4	4	5	4	5
4	4	5	3	5	3	5	4	4	3
3	4	3	4	4	3	5	3	3	3
4	5	4	4	5	5	4	4	3	4
4	3	3	5	4	4	5	4	5	3
2	2	1	1	4	4	5	4	4	4
1	1	1	1	5	3	3	5	5	4
4	3	4	4	3	3	5	3	4	4
4	4	4	4	3	4	5	3	3	5
5	4	4	4	3	5	3	4	3	4
4	4	3	4	3	3	5	4	4	4
4	4	3	4	3	5	3	3	4	4
3	2	3	3	2	3	2	3	3	3
4	4	5	3	4	4	4	3	5	4
3	4	4	5	4	4	3	4	5	4
2	1	2	2	4	4	4	4	5	3
1	2	3	1	1	2	1	2	2	2
3	5	5	4	4	4	4	4	4	4
2	2	2	2	2	2	3	2	1	3
5	3	5	5	3	3	4	4	5	4

4	5	4	4	3	4	3	4	3	5
3	4	5	5	3	5	3	4	4	4
3	4	4	3	3	5	4	3	3	4
4	4	4	4	3	4	3	4	4	4
3	4	4	4	4	5	5	4	5	4
5	4	3	3	5	4	1	2	2	1
2	1	3	2	2	3	1	3	1	3
4	4	3	5	3	5	5	3	4	4
5	4	4	3	3	3	4	3	3	4
3	3	3	4	5	5	5	4	3	5
4	4	4	4	4	5	5	5	5	3
2	2	1	2	1	1	3	1	2	3
3	4	3	5	3	4	5	4	5	4
4	5	4	5	4	4	3	4	4	4
5	4	5	4	4	4	3	5	5	3
4	3	3	4	4	4	3	4	4	4
3	5	5	5	5	3	1	2	1	2
4	4	3	4	5	5	4	5	4	3
3	1	2	1	3	2	3	2	1	1
4	4	5	5	4	5	4	4	3	5
5	3	5	4	3	5	4	5	3	4
3	3	4	3	4	5	4	4	4	3
5	3	4	4	5	3	3	4	4	5
3	3	4	4	4	5	5	4	3	5
5	3	4	4	4	5	5	3	4	4
3	3	4	5	1	2	4	4	3	5
3	4	5	3	4	4	4	4	4	4
4	4	4	4	3	4	3	4	4	3
5	3	3	3	4	5	5	4	4	4
4	5	5	4	5	4	4	4	5	4
4	4	4	4	3	5	3	5	4	3
4	5	4	5	3	4	3	4	3	5
3	3	5	3	5	4	5	4	4	4
4	4	5	5	4	5	4	4	4	5
4	4	4	4	4	4	3	5	5	4
4	4	4	3	4	5	5	4	4	4
3	5	4	4	4	5	4	5	3	4
4	3	5	3	5	4	4	5	3	4
4	4	3	3	5	4	4	4	5	4
3	3	4	3	3	3	4	3	5	5
5	4	5	5	4	4	4	3	4	4
2	1	2	1	3	2	2	2	1	1
5	5	4	4	5	4	5	4	3	4
5	4	3	3	4	4	4	3	5	3
3	3	2	1	2	2	3	2	3	2
3	4	4	4	3	5	3	3	4	5
4	4	5	4	4	3	4	5	4	3

3	2	3	2	3	2	2	2	1	3
4	4	5	5	3	4	4	4	4	3
4	4	4	4	4	3	4	4	4	4
5	4	4	3	4	4	5	5	4	5
4	4	4	4	3	3	3	4	3	4
3	5	3	5	4	4	3	3	3	3
5	4	4	4	4	4	3	4	5	5
4	5	5	3	3	5	3	5	4	4
3	4	5	4	4	5	4	4	5	5
4	3	4	3	3	3	4	4	3	4
3	5	4	5	5	3	5	3	4	4
4	4	5	5	3	3	4	4	3	3
1	1	3	3	2	1	2	3	1	2
5	4	3	3	5	4	5	3	5	5
4	4	4	4	3	3	5	4	4	4
3	3	3	4	4	4	3	5	5	4
4	5	4	3	5	4	3	3	3	3
4	3	5	3	5	4	4	5	4	4
4	4	3	4	5	4	4	5	3	3
4	5	3	3	3	4	3	4	3	5
4	5	3	3	5	4	4	3	4	4
4	5	3	4	5	4	4	4	5	3
3	4	4	4	4	5	5	5	4	4
4	4	5	5	4	4	4	4	4	4
3	4	4	4	4	4	4	5	5	3
4	4	4	5	4	5	5	5	3	3
1	1	3	2	2	3	1	2	2	2
5	4	4	4	3	4	5	3	4	4
5	5	3	3	3	3	5	3	4	3
3	4	4	3	3	3	5	3	4	5
5	4	4	4	4	3	4	4	4	4
3	4	3	3	4	4	3	4	5	4
4	3	3	3	5	4	5	5	4	5
4	4	5	4	5	4	5	3	3	5
4	3	5	4	5	3	5	3	5	5
5	5	4	3	4	4	5	5	3	5
4	4	5	5	3	3	4	4	3	3
5	4	5	4	3	3	4	5	5	5
2	2	2	2	3	3	2	2	2	2
5	4	3	5	5	5	4	4	4	5
4	5	5	4	3	3	4	3	4	3
1	1	1	1	4	4	4	4	4	4
4	4	4	5	4	5	3	4	4	5
3	4	3	3	4	3	4	4	3	4
4	5	3	5	3	4	1	1	2	1
4	3	3	5	4	5	5	4	4	4
4	4	3	4	5	5	3	5	3	4

3	3	5	3	4	3	4	4	4	4
4	4	5	5	3	3	1	2	1	1
3	3	5	5	1	2	3	4	3	4
3	4	4	4	5	4	4	4	5	4
5	5	5	4	4	5	4	3	5	3
4	4	4	4	4	3	5	5	4	4
1	3	3	1	3	3	3	3	1	1
5	3	3	4	5	4	4	3	4	4
5	5	5	3	4	3	4	4	5	3
4	3	3	4	2	1	3	4	4	4
4	4	4	4	4	4	4	4	4	4
5	4	4	5	4	4	4	5	4	4
4	4	3	3	4	4	4	3	3	4
4	4	4	4	3	3	3	5	4	4
5	4	3	4	4	4	3	4	5	4
3	3	3	4	4	3	4	4	5	4
3	2	2	2	2	2	3	1	2	3
4	4	3	5	2	1	3	4	4	5
2	1	2	1	4	3	4	3	4	4
5	4	4	5	4	5	4	3	5	4
1	1	2	2	3	4	4	4	4	5
2	3	2	2	2	2	3	2	2	2
5	4	3	3	3	4	1	2	2	2
5	3	3	3	3	4	5	4	3	4
4	3	4	4	5	3	4	4	5	4
4	4	3	4	5	4	5	4	4	5
4	5	5	4	4	5	4	5	4	3
5	3	3	5	4	5	4	5	3	4
5	4	3	3	3	5	5	4	4	4
5	3	3	4	5	5	4	5	5	4
4	4	3	4	3	5	4	3	4	4
4	4	4	5	3	3	4	4	3	3
5	3	4	4	4	5	4	5	4	4
4	3	5	4	4	4	4	4	5	4
4	4	4	3	3	3	4	3	4	4
3	4	3	4	5	4	2	1	2	1
3	4	4	3	3	3	4	4	3	3
5	5	5	5	4	4	4	5	3	3
4	3	5	4	2	1	5	4	4	5
4	5	4	5	5	4	4	4	4	5
3	2	3	2	2	2	2	2	1	2
2	2	1	1	2	2	3	1	1	2
4	3	4	4	4	4	4	4	4	5
4	4	3	3	5	4	5	4	5	4
5	4	4	4	4	3	3	4	3	4
5	3	4	4	4	5	4	5	4	4
4	3	4	3	4	5	3	4	4	5

3	3	4	5	4	3	5	4	3	5
3	1	2	2	2	2	2	1	1	2
4	4	4	3	3	3	4	5	5	5
2	2	1	1	5	4	3	5	4	4
3	3	4	5	4	4	5	4	4	5
3	2	2	2	2	3	1	1	3	3
4	4	4	4	3	4	4	5	4	4
4	4	4	5	4	4	4	5	4	3
5	5	3	4	4	4	3	4	4	4
4	5	4	4	5	4	3	3	4	5
2	1	1	1	4	4	3	4	3	4
5	4	4	4	3	5	3	5	3	4
1	3	1	1	2	2	2	2	1	2
3	2	1	2	3	3	2	3	3	3
2	2	2	1	2	2	2	1	2	2
4	4	4	4	3	3	4	3	3	5
3	5	4	4	5	5	3	5	4	3
3	3	4	4	5	5	4	5	3	3
5	3	4	5	3	4	1	1	2	1
5	5	5	5	1	1	5	5	4	5
2	2	1	2	3	2	3	3	2	2
4	4	4	4	4	3	3	4	5	3
3	4	4	3	4	4	4	5	4	3
3	4	5	4	4	5	3	3	4	5
3	4	5	4	4	4	4	4	5	5
4	4	5	4	4	3	4	4	4	4
4	4	3	4	1	1	4	4	4	4
2	3	3	2	2	3	1	2	3	2
4	5	4	4	3	4	4	5	4	4
5	4	5	4	3	4	4	3	4	5
2	2	3	1	2	2	2	2	1	3
4	3	4	4	4	4	1	1	1	2
3	4	5	4	3	3	1	1	1	2
3	1	2	1	2	2	2	2	2	3
5	5	5	4	5	5	2	1	2	1
5	3	5	4	4	4	3	4	4	4
4	4	5	3	5	4	4	5	5	4
5	3	3	3	4	4	5	4	4	4
5	4	4	3	3	4	5	4	5	4
4	4	5	4	4	3	3	4	3	5
4	4	4	3	3	4	3	5	3	5
5	3	4	4	4	5	5	4	4	4
4	5	3	3	3	4	3	3	3	4
2	3	2	3	1	2	2	1	2	2
4	5	4	4	4	3	4	4	4	4
4	5	5	4	3	4	3	4	5	5
3	4	3	5	3	4	3	5	4	3

4	5	3	3	5	3	4	4	3	5
3	3	5	4	3	4	4	3	4	3
5	5	4	4	5	5	4	4	4	4
5	5	5	5	5	3	4	3	3	4
3	5	3	5	5	3	4	3	3	4
5	4	5	3	3	4	5	5	4	4
3	5	5	3	3	5	3	4	3	4
3	5	5	4	3	3	5	3	3	4
3	5	4	4	1	1	3	5	5	4
4	4	5	4	2	2	3	3	5	4
4	4	4	3	5	3	3	4	4	3
4	4	4	4	4	3	4	5	3	5
5	5	5	5	3	5	4	4	4	3
5	5	3	4	3	4	2	1	1	2
5	4	3	3	3	5	4	5	3	5
4	4	3	4	4	4	4	3	4	4
5	4	3	5	4	3	3	4	5	5
4	4	5	3	5	4	3	4	4	5
3	4	4	4	4	3	5	4	5	5
4	4	3	4	4	3	4	3	4	5
3	4	4	5	3	5	3	4	3	4
4	4	3	5	4	3	4	3	5	5
5	5	3	5	4	3	4	3	5	5
3	3	4	5	4	5	3	5	3	4
3	2	1	2	1	2	2	2	2	2
1	2	1	2	4	4	5	3	4	4
1	1	2	1	3	3	4	3	5	4
4	5	4	4	5	4	5	4	5	3
4	3	3	3	4	5	5	4	4	5
4	5	3	4	3	3	5	5	3	4
1	3	2	2	2	1	2	3	1	2
5	5	4	4	4	3	4	4	4	5
4	4	4	3	4	5	5	5	4	4
5	5	4	3	3	3	3	4	5	3
4	5	4	3	4	5	5	4	4	3
3	4	4	3	3	4	4	5	4	3
5	4	3	4	2	2	4	4	5	4
3	5	4	5	4	3	3	4	4	4
4	4	5	5	4	4	4	5	4	4
1	3	2	2	2	3	1	2	2	2
3	3	4	4	4	4	5	4	3	4
5	4	4	3	3	4	4	4	5	3
3	3	3	3	3	2	2	2	1	3
2	2	1	1	3	2	2	2	1	2
4	4	4	5	4	3	2	1	2	1
5	4	4	5	5	4	4	4	4	5
3	4	3	5	4	4	4	4	4	3

4	3	4	3	5	4	4	5	5	5
4	3	4	3	4	4	4	4	5	5
5	4	4	4	3	5	5	3	4	4
4	3	3	5	4	4	4	4	5	4
3	4	5	5	4	4	4	4	3	5
3	5	4	4	4	4	4	3	5	4
4	3	5	3	4	4	3	3	4	3
3	4	4	3	4	4	3	5	3	4
4	3	5	4	3	3	4	4	4	4
3	4	3	3	5	4	4	5	4	3
5	5	4	4	5	4	4	4	4	3
5	4	5	5	4	3	4	4	4	5
3	5	4	4	3	3	1	1	1	2
2	2	1	2	5	3	4	4	5	3
3	3	3	2	3	1	3	2	1	3
4	4	4	5	4	4	3	4	3	4
4	4	4	5	5	4	5	3	3	3
4	4	5	5	3	4	5	4	4	4
1	1	1	1	5	3	3	4	4	3
5	4	3	4	4	5	3	4	4	5
4	4	4	4	3	3	4	4	4	3
3	5	5	4	4	5	3	3	5	4
5	5	4	5	4	4	4	4	5	5
5	4	4	4	4	4	4	5	3	5
5	3	5	3	5	3	3	5	5	5
1	1	3	1	1	2	1	1	2	2
4	5	4	3	4	4	4	5	3	3
3	3	4	3	4	4	5	4	3	4
4	3	4	5	5	3	4	5	4	4
5	4	3	4	1	2	4	4	3	4
5	3	3	5	5	5	4	4	5	4
5	4	4	3	5	3	5	3	3	4
3	3	2	3	3	3	2	3	1	1
2	2	2	2	3	2	2	3	3	2
1	1	2	2	2	2	1	2	2	1
3	4	4	5	3	4	4	3	3	4
5	4	5	5	5	4	3	3	4	5
3	3	5	3	3	4	3	3	3	4
3	4	4	3	3	5	4	3	4	4
5	5	3	4	3	5	4	5	4	4
4	4	4	3	4	3	1	2	1	2
5	3	3	4	3	4	5	4	4	5
5	5	4	5	4	5	4	4	4	4
5	5	4	3	4	4	4	5	4	4
4	4	5	3	4	4	4	4	4	4
3	4	4	3	3	3	3	3	4	3
3	4	4	5	5	3	4	4	3	5

5	4	5	5	4	3	5	4	3	4
4	3	4	5	4	4	4	4	5	5
5	5	4	4	4	5	4	5	4	5
5	4	4	4	4	4	3	4	4	4
3	4	4	4	3	3	5	4	4	3
5	4	4	5	4	5	4	3	3	4
2	3	3	1	1	3	1	1	3	2
5	4	3	4	5	4	4	4	3	5
3	2	1	3	3	1	3	1	2	2
4	5	3	3	5	4	4	4	4	5
3	5	4	4	4	4	4	3	4	5
5	4	4	4	3	4	5	3	5	3
4	4	4	5	4	5	3	5	5	4
1	2	2	1	3	3	4	5	4	4
2	3	3	2	2	1	1	3	2	2
3	4	3	4	3	3	4	5	5	4
2	2	1	1	4	4	5	5	3	5
4	4	5	3	1	2	5	3	5	3
4	3	4	5	4	4	5	3	4	4
3	4	5	4	4	5	4	4	3	5
3	1	2	1	1	2	2	2	2	2
3	1	3	1	3	2	2	2	2	2
4	4	4	5	1	1	4	4	3	4
3	4	4	5	4	4	4	3	5	4
4	3	4	5	4	4	4	4	5	5
4	4	5	4	5	5	2	2	1	2
4	4	3	5	3	3	5	4	4	3
3	4	5	4	4	5	5	4	4	3
3	4	4	4	4	3	3	4	4	3
3	4	5	4	3	4	5	4	4	4
4	5	5	3	3	4	5	4	4	4
4	4	5	4	4	4	5	5	3	5
5	3	4	5	5	3	3	4	5	3
5	5	4	3	4	4	4	3	4	5
3	5	5	3	3	4	4	5	4	5
3	5	4	4	4	4	4	4	5	3
3	5	3	5	3	3	4	5	3	3
3	1	2	2	2	1	2	1	2	2
4	4	4	4	3	4	4	4	3	3
1	3	2	2	3	1	2	1	2	3
4	3	4	4	4	4	4	3	4	4
4	5	3	4	5	5	3	5	3	4
4	5	4	4	5	3	4	4	5	4
4	5	4	5	4	5	3	4	3	4
4	4	5	5	3	4	4	5	5	4
3	1	2	3	3	1	1	2	1	2
5	5	5	4	5	5	5	5	4	5

4	4	4	4	4	3	4	4	3	4
2	2	3	3	2	2	2	3	3	3
4	3	3	3	3	4	3	3	3	4
4	4	5	3	5	3	3	4	4	5
4	4	5	4	4	4	4	5	5	4
5	3	3	5	3	4	5	4	4	4
3	4	3	3	5	4	5	4	4	3
2	2	2	2	5	5	4	5	4	4
4	4	4	5	5	4	4	4	4	3
4	5	4	5	5	4	4	4	4	4
4	4	4	4	4	3	4	4	5	4
5	3	4	4	4	4	3	4	4	3
4	4	4	4	4	4	4	4	4	3
5	3	4	3	1	2	3	4	3	5
3	2	2	3	2	2	3	2	2	3
3	4	4	4	5	5	4	5	5	4
3	3	4	4	4	4	4	3	5	4
5	3	4	5	5	3	3	4	4	4
4	3	4	4	3	4	5	4	4	5
5	3	3	4	5	4	3	5	4	4
5	4	3	3	4	5	4	4	4	4
3	3	5	5	5	4	5	5	5	5
3	3	5	4	4	4	3	4	5	4
5	4	4	4	3	5	3	4	3	4
4	5	5	3	5	4	3	4	4	4
4	5	3	3	5	3	4	5	5	4
4	4	4	5	3	4	2	2	1	1
4	4	4	4	5	3	4	4	4	3
3	4	5	3	5	4	5	4	5	4
4	5	4	4	5	5	5	4	4	5
4	4	5	4	4	3	4	4	5	3
2	2	2	2	3	2	3	2	2	2
2	1	1	1	3	2	3	2	1	2
4	5	4	4	4	4	4	4	4	3
4	5	3	5	5	5	3	4	5	4
5	4	4	3	3	4	5	4	3	4
3	5	4	5	4	5	4	5	4	3
4	4	4	5	1	2	3	4	5	5
4	4	3	5	5	5	4	4	5	4
2	1	1	3	3	2	1	2	2	1
2	2	1	2	2	1	2	2	1	2
4	3	4	5	4	3	4	5	5	4
3	5	5	4	5	4	4	5	3	5
1	2	1	2	4	4	4	4	5	4
5	3	4	4	4	3	5	3	4	4
2	1	2	2	2	2	2	2	2	3
4	4	4	3	3	3	4	4	5	4

3	5	4	4	3	5	4	4	5	5
1	1	2	2	2	1	1	3	2	2
4	4	3	3	2	1	3	5	4	5
5	5	3	4	3	3	5	5	4	3
3	4	4	5	4	4	5	5	3	4
4	5	5	5	2	2	4	4	3	4
5	5	4	3	3	4	4	5	3	4
3	2	2	2	3	3	2	3	2	1
4	4	5	3	4	4	4	4	4	4
4	3	5	3	4	4	5	5	5	4
4	4	3	4	5	4	4	4	4	3
5	3	5	4	5	5	4	3	3	5
3	3	4	4	3	3	5	4	3	4
2	2	1	2	2	3	3	2	2	3
3	4	4	3	4	4	4	4	5	4
4	4	5	3	5	4	3	5	3	4
5	4	5	4	5	3	4	4	5	5
5	5	5	4	4	5	5	5	3	3
4	5	4	3	3	5	4	5	4	3
4	4	4	5	3	5	5	5	4	4
4	5	4	4	4	3	5	5	5	4
5	4	5	4	3	3	2	1	1	2
2	3	2	1	3	1	2	2	2	3
1	2	3	2	2	1	2	2	3	3
3	5	5	5	5	3	5	3	4	5
5	4	4	5	4	5	5	4	4	5
5	4	4	4	3	3	3	3	5	4
4	4	5	4	4	3	4	4	4	3
4	4	4	3	3	3	4	4	4	4
3	4	4	5	4	4	4	5	4	4
4	4	5	3	4	3	3	3	4	4
3	4	5	4	3	5	5	4	3	3
5	4	4	4	3	3	3	5	4	3
3	4	4	3	5	4	2	1	1	2
4	4	3	4	4	4	4	4	5	4
4	5	5	4	4	3	4	4	4	4
4	4	4	5	5	3	5	4	3	3
4	5	4	5	4	5	4	4	4	3
1	2	2	2	1	2	2	2	2	1
5	5	3	4	4	3	3	4	4	4
4	5	3	4	4	4	4	5	4	5
4	3	3	4	4	4	3	5	5	4
4	5	4	5	5	3	4	5	5	4
5	4	5	4	4	3	4	4	5	3
4	4	5	4	3	4	5	5	3	3
4	3	4	4	3	4	2	2	1	2
5	4	3	3	4	4	4	3	4	5

4	3	4	5	3	3	3	4	5	4
3	4	3	3	5	3	4	5	4	5
2	2	2	2	4	4	4	3	5	4
3	3	4	5	5	3	1	1	2	1
3	3	2	1	3	3	2	1	2	2
1	2	2	2	1	1	2	2	2	1
3	5	4	4	2	1	4	4	4	4
1	2	2	3	2	2	2	2	1	2
4	4	4	5	5	5	4	3	5	4
5	3	4	4	5	3	3	4	5	5
4	4	4	3	4	5	3	4	4	3
3	4	5	4	3	3	4	4	4	4
3	2	2	3	1	3	1	2	2	2
1	1	2	1	5	3	3	5	4	5
4	4	4	3	5	4	3	4	5	5
3	3	3	3	4	3	4	3	5	3
3	3	5	5	4	4	4	4	4	4
5	4	4	3	4	4	5	4	3	3
5	5	4	4	4	4	4	5	4	5
4	4	5	3	5	3	5	4	4	3
3	4	3	4	4	3	5	3	3	3
4	5	4	4	5	5	4	4	3	4
4	3	3	5	4	4	5	4	5	3
2	2	1	1	4	4	5	4	4	4
1	1	1	1	5	3	3	5	5	4
4	3	4	4	3	3	5	3	4	4
4	4	4	4	3	4	5	3	3	5
5	4	4	4	3	5	3	4	3	4
4	4	3	4	3	3	5	4	4	4
4	4	3	4	3	5	3	3	4	4
3	2	3	3	2	3	2	3	3	3
4	4	5	3	4	4	4	3	5	4
3	4	4	5	4	4	3	4	5	4
2	1	2	2	4	4	4	4	5	3
1	2	3	1	1	2	1	2	2	2
3	5	5	4	4	4	4	4	4	4
2	2	2	2	2	2	3	2	1	3
5	3	5	5	3	3	4	4	5	4
4	5	4	4	3	4	3	4	3	5
3	4	5	5	3	5	3	4	4	4
3	4	4	3	3	5	4	3	3	4
4	4	4	4	3	4	3	4	4	4
3	4	4	4	4	5	5	4	5	4
5	4	3	3	5	4	1	2	2	1
2	1	3	2	2	3	1	3	1	3
4	4	3	5	3	5	5	3	4	4
5	4	4	3	3	3	4	3	3	4

3	3	3	4	5	5	5	4	3	5
4	4	4	4	4	5	5	5	5	3
2	2	1	2	1	1	3	1	2	3
3	4	3	5	3	4	5	4	5	4
4	5	4	5	4	4	3	4	4	4
5	4	5	4	4	4	3	5	5	3
4	3	3	4	4	4	3	4	4	4
3	5	5	5	5	3	1	2	1	2
4	4	3	4	5	5	4	5	4	3
3	1	2	1	3	2	3	2	1	1
4	4	5	5	4	5	4	4	3	5
5	3	5	4	3	5	4	5	3	4
3	3	4	3	4	5	4	4	4	3
5	3	4	4	5	3	3	4	4	5
3	3	4	4	4	5	5	4	3	5
5	3	4	4	4	5	5	3	4	4
3	3	4	5	1	2	4	4	3	5
3	4	5	3	4	4	4	4	4	4
4	4	4	4	3	4	3	4	4	3
5	3	3	3	4	5	5	4	4	4
4	5	5	4	5	4	4	4	5	4
4	4	4	4	3	5	3	5	4	3
4	5	4	5	3	4	3	4	3	5
3	3	5	3	5	4	5	4	4	4
4	4	5	5	4	5	4	4	4	5
4	4	4	4	4	4	3	5	5	4
4	4	4	3	4	5	5	4	4	4
3	5	4	4	4	5	4	5	3	4
4	3	5	3	5	4	4	5	3	4
4	4	3	3	5	4	4	4	5	4
3	3	4	3	3	3	4	3	5	5
5	4	5	5	4	4	4	3	4	4
2	1	2	1	3	2	2	2	1	1
5	5	4	4	5	4	5	4	3	4
5	4	3	3	4	4	4	3	5	3
3	3	2	1	2	2	3	2	3	2
3	4	4	4	3	5	3	3	4	5
4	4	5	4	4	3	4	5	4	3
3	2	3	2	3	2	2	2	1	3
4	4	5	5	3	4	4	4	4	3
4	4	4	4	4	3	4	4	4	4
5	4	4	3	4	4	5	5	4	5
4	4	4	4	3	3	3	4	3	4
3	5	3	5	4	4	3	3	3	3
5	4	4	4	4	4	3	4	5	5
4	5	5	3	3	5	3	5	4	4
3	4	5	4	4	5	4	4	5	5

4	3	4	3	3	3	4	4	3	4
3	5	4	5	5	3	5	3	4	4
4	4	5	5	3	3	4	4	3	3
1	1	3	3	2	1	2	3	1	2
5	4	3	3	5	4	5	3	5	5
4	4	4	4	3	3	5	4	4	4
3	3	3	4	4	4	3	5	5	4
4	5	4	3	5	4	3	3	3	3
4	3	5	3	5	4	4	5	4	4
4	4	3	4	5	4	4	5	3	3
4	5	3	3	3	4	3	4	3	5
4	5	3	3	5	4	4	3	4	4
4	5	3	4	5	4	4	4	5	3
3	4	4	4	4	5	5	5	4	4
4	4	5	5	4	4	4	4	4	4
3	4	4	4	4	4	4	5	5	3
4	4	4	5	4	5	5	5	3	3
1	1	3	2	2	3	1	2	2	2
5	4	4	4	3	4	5	3	4	4
5	5	3	3	3	3	5	3	4	3
3	4	4	3	3	3	5	3	4	5
5	4	4	4	4	3	4	4	4	4
3	4	3	3	4	4	3	4	5	4
4	3	3	3	5	4	5	5	4	5
4	4	5	4	5	4	5	3	3	5
4	3	5	4	5	3	5	3	5	5
5	5	4	3	4	4	5	5	3	5
4	4	5	5	3	3	4	4	3	3
5	4	5	4	3	3	4	5	5	5
2	2	2	2	3	3	2	2	2	2
5	4	3	5	5	5	4	4	4	5
4	5	5	4	3	3	4	3	4	3
1	1	1	1	4	4	4	4	4	4
4	4	4	5	4	5	3	4	4	5
3	4	3	3	4	3	4	4	3	4
4	5	3	5	3	4	1	1	2	1
4	3	3	5	4	5	5	4	4	4
4	4	3	4	5	5	3	5	3	4
3	3	5	3	4	3	4	4	4	4
4	4	5	5	3	3	1	2	1	1
3	3	5	5	1	2	3	4	3	4
3	4	4	4	5	4	4	4	5	4
5	5	5	4	4	5	4	3	5	3
4	4	4	4	4	3	5	5	4	4
1	3	3	1	3	3	3	3	1	1
5	3	3	4	5	4	4	3	4	4
5	5	5	3	4	3	4	4	5	3

4	3	3	4	2	1	3	4	4	4
4	4	4	4	4	4	4	4	4	4
5	4	4	5	4	4	4	5	4	4
4	4	3	3	4	4	4	3	3	4
4	4	4	4	3	3	3	5	4	4
5	4	3	4	4	4	3	4	5	4
3	3	3	4	4	3	4	4	5	4
3	2	2	2	2	2	3	1	2	3
4	4	3	5	2	1	3	4	4	5
2	1	2	1	4	3	4	3	4	4
5	4	4	5	4	5	4	3	5	4
1	1	2	2	3	4	4	4	4	5
2	3	2	2	2	2	3	2	2	2
5	4	3	3	3	4	1	2	2	2
5	3	3	3	3	4	5	4	3	4
4	3	4	4	5	3	4	4	5	4
4	4	3	4	5	4	5	4	4	5
4	5	5	4	4	5	4	5	4	3
5	3	3	5	4	5	4	5	3	4
5	4	3	3	3	5	5	4	4	4
5	3	3	4	5	5	4	5	5	4
4	4	3	4	3	5	4	3	4	4
4	4	4	5	3	3	4	4	3	3
5	3	4	4	4	5	4	5	4	4
4	3	5	4	4	4	4	4	5	4
4	4	4	3	3	3	4	3	4	4
3	4	3	4	5	4	2	1	2	1
3	4	4	3	3	3	4	4	3	3
5	5	5	5	4	4	4	5	3	3
4	3	5	4	2	1	5	4	4	5
4	5	4	5	5	4	4	4	4	5
3	2	3	2	2	2	2	2	1	2
2	2	1	1	2	2	3	1	1	2
4	3	4	4	4	4	4	4	4	5
4	4	3	3	5	4	5	4	5	4
5	4	4	4	4	3	3	4	3	4
5	3	4	4	4	5	4	5	4	4
4	3	4	3	4	5	3	4	4	5
3	3	4	5	4	3	5	4	3	5
3	1	2	2	2	2	2	1	1	2
4	4	4	3	3	3	4	5	5	5
2	2	1	1	5	4	3	5	4	4
3	3	4	5	4	4	5	4	4	5
3	2	2	2	2	3	1	1	3	3
4	4	4	4	3	4	4	5	4	4
4	4	4	5	4	4	4	5	4	3
5	5	3	4	4	4	3	4	4	4

4	5	4	4	5	4	3	3	4	5
2	1	1	1	4	4	3	4	3	4
5	4	4	4	3	5	3	5	3	4
1	3	1	1	2	2	2	2	1	2
3	2	1	2	3	3	2	3	3	3
2	2	2	1	2	2	2	1	2	2
4	4	4	4	3	3	4	3	3	5
3	5	4	4	5	5	3	5	4	3
3	3	4	4	5	5	4	5	3	3
5	3	4	5	3	4	1	1	2	1
5	5	5	5	1	1	5	5	4	5
2	2	1	2	3	2	3	3	2	2
4	4	4	4	4	3	3	4	5	3
3	4	4	3	4	4	4	5	4	3
3	4	5	4	4	5	3	3	4	5
3	4	5	4	4	4	4	4	5	5
4	4	5	4	4	3	4	4	4	4
4	4	3	4	1	1	4	4	4	4
2	3	3	2	2	3	1	2	3	2
4	5	4	4	3	4	4	5	4	4
5	4	5	4	3	4	4	3	4	5
2	2	3	1	2	2	2	2	1	3
4	3	4	4	4	4	1	1	1	2
3	4	5	4	3	3	1	1	1	2
3	1	2	1	2	2	2	2	2	3
5	5	5	4	5	5	2	1	2	1
5	3	5	4	4	4	3	4	4	4
4	4	5	3	5	4	4	5	5	4
5	3	3	3	4	4	5	4	4	4
5	4	4	3	3	4	5	4	5	4
4	4	5	4	4	3	3	4	3	5
4	4	4	3	3	4	3	5	3	5
5	3	4	4	4	5	5	4	4	4
4	5	3	3	3	4	3	3	3	4
2	3	2	3	1	2	2	1	2	2
4	5	4	4	4	3	4	4	4	4
4	5	5	4	3	4	3	4	5	5
3	4	3	5	3	4	3	5	4	3
4	5	3	3	5	3	4	4	3	5
3	3	5	4	3	4	4	3	4	3
5	5	4	4	5	5	4	4	4	4
5	5	5	5	5	3	4	3	3	4
3	5	3	5	5	3	4	3	3	4
5	4	5	3	3	4	5	5	4	4
3	5	5	3	3	5	3	4	3	4
3	5	5	4	3	3	5	3	3	4
3	5	4	4	1	1	3	5	5	4

4	4	5	4	2	2	3	3	5	4
4	4	4	3	5	3	3	4	4	3
4	4	4	4	4	3	4	5	3	5
5	5	5	5	3	5	4	4	4	3
5	5	3	4	3	4	2	1	1	2
5	4	3	3	3	5	4	5	3	5
4	4	3	4	4	4	4	3	4	4
5	4	3	5	4	3	3	4	5	5
4	4	5	3	5	4	3	4	4	5
3	4	4	4	4	3	5	4	5	5
4	4	3	4	4	3	4	3	4	5
3	4	4	5	3	5	3	4	3	4
4	4	3	5	4	3	4	3	5	5
5	5	3	5	4	3	4	3	5	5
3	3	4	5	4	5	3	5	3	4
3	2	1	2	1	2	2	2	2	2
1	2	1	2	4	4	5	3	4	4
1	1	2	1	3	3	4	3	5	4
4	5	4	4	5	4	5	4	5	3
4	3	3	3	4	5	5	4	4	5
4	5	3	4	3	3	5	5	3	4
1	3	2	2	2	1	2	3	1	2
5	5	4	4	4	3	4	4	4	5
4	4	4	3	4	5	5	5	4	4
5	5	4	3	3	3	3	4	5	3
4	5	4	3	4	5	5	4	4	3
3	4	4	3	3	4	4	5	4	3
5	4	3	4	2	2	4	4	5	4
3	5	4	5	4	3	3	4	4	4
4	4	5	5	4	4	4	5	4	4
1	3	2	2	2	3	1	2	2	2
3	3	4	4	4	4	5	4	3	4
5	4	4	3	3	4	4	4	5	3
3	3	3	3	3	2	2	2	1	3
2	2	1	1	3	2	2	2	1	2
4	4	4	5	4	3	2	1	2	1
5	4	4	5	5	4	4	4	4	5
3	4	3	5	4	4	4	4	4	3
4	3	4	3	5	4	4	5	5	5
4	3	4	3	4	4	4	4	5	5
5	4	4	4	3	5	5	3	4	4
4	3	3	5	4	4	4	4	5	4
3	4	5	5	4	4	4	4	3	5
3	5	4	4	4	4	4	3	5	4
4	3	5	3	4	4	3	3	4	3
3	4	4	3	4	4	3	5	3	4
4	3	5	4	3	3	4	4	4	4

3	4	3	3	5	4	4	5	4	3
5	5	4	4	5	4	4	4	4	3
5	4	5	5	4	3	4	4	4	5
3	5	4	4	3	3	1	1	1	2
2	2	1	2	5	3	4	4	5	3
3	3	3	2	3	1	3	2	1	3
4	4	4	5	4	4	3	4	3	4
4	4	4	5	5	4	5	3	3	3
4	4	5	5	3	4	5	4	4	4
1	1	1	1	5	3	3	4	4	3
5	4	3	4	4	5	3	4	4	5
4	4	4	4	3	3	4	4	4	3
3	5	5	4	4	5	3	3	5	4
5	5	4	5	4	4	4	4	5	5
5	4	4	4	4	4	4	5	3	5
5	3	5	3	5	3	3	5	5	5
1	1	3	1	1	2	1	1	2	2
4	5	4	3	4	4	4	5	3	3
3	3	4	3	4	4	5	4	3	4
4	3	4	5	5	3	4	5	4	4
5	4	3	4	1	2	4	4	3	4
5	3	3	5	5	5	4	4	5	4
5	4	4	3	5	3	5	3	3	4
3	3	2	3	3	3	2	3	1	1
2	2	2	2	3	2	2	3	3	2
1	1	2	2	2	2	1	2	2	1
3	4	4	5	3	4	4	3	3	4
5	4	5	5	5	4	3	3	4	5
3	3	5	3	3	4	3	3	3	4
3	4	4	3	3	5	4	3	4	4
5	5	3	4	3	5	4	5	4	4
4	4	4	3	4	3	1	2	1	2
5	3	3	4	3	4	5	4	4	5
5	5	4	5	4	5	4	4	4	4
5	5	4	3	4	4	4	5	4	4
4	4	5	3	4	4	4	4	4	4
3	4	4	3	3	3	3	3	4	3
3	4	4	5	5	3	4	4	3	5
5	4	5	5	4	3	5	4	3	4
4	3	4	5	4	4	4	4	5	5
5	5	4	4	4	5	4	5	4	5
5	4	4	4	4	4	3	4	4	4
3	4	4	4	3	3	5	4	4	3
5	4	4	5	4	5	4	3	3	4
2	3	3	1	1	3	1	1	3	2
5	4	3	4	5	4	4	4	3	5
3	2	1	3	3	1	3	1	2	2

4	5	3	3	5	4	4	4	4	5
3	5	4	4	4	4	4	3	4	5
5	4	4	4	3	4	5	3	5	3
4	4	4	5	4	5	3	5	5	4
1	2	2	1	3	3	4	5	4	4
2	3	3	2	2	1	1	3	2	2
3	4	3	4	3	3	4	5	5	4
2	2	1	1	4	4	5	5	3	5
4	4	5	3	1	2	5	3	5	3
4	3	4	5	4	4	5	3	4	4
3	4	5	4	4	5	4	4	3	5
3	1	2	1	1	2	2	2	2	2
3	1	3	1	3	2	2	2	2	2
4	4	4	5	1	1	4	4	3	4
3	4	4	5	4	4	4	3	5	4
4	3	4	5	4	4	4	4	5	5
4	4	5	4	5	5	2	2	1	2
4	4	3	5	3	3	5	4	4	3
3	4	5	4	4	5	5	4	4	3
3	4	4	4	4	3	3	4	4	3
3	4	5	4	3	4	5	4	4	4
4	5	5	3	3	4	5	4	4	4
4	4	5	4	4	4	5	5	3	5
5	3	4	5	5	3	3	4	5	3
5	5	4	3	4	4	4	3	4	5
3	5	5	3	3	4	4	5	4	5
3	5	4	4	4	4	4	4	5	3
3	5	3	5	3	3	4	5	3	3
3	1	2	2	2	1	2	1	2	2
4	4	4	4	3	4	4	4	3	3
1	3	2	2	3	1	2	1	2	3
4	3	4	4	4	4	4	3	4	4
4	5	3	4	5	5	3	5	3	4
4	5	4	4	5	3	4	4	5	4
4	5	4	5	4	5	3	4	3	4
4	4	5	5	3	4	4	5	5	4
3	1	2	3	3	1	1	2	1	2
5	5	5	4	5	5	5	5	4	5
4	4	4	4	4	3	4	4	3	4
2	2	3	3	2	2	2	3	3	3
4	3	3	3	3	4	3	3	3	4
4	4	5	3	5	3	3	4	4	5
4	4	5	4	4	4	4	5	5	4
5	3	3	5	3	4	5	4	4	4
3	4	3	3	5	4	5	4	4	3
2	2	2	2	5	5	4	5	4	4
4	4	4	5	5	4	4	4	4	3

4	5	4	5	5	4	4	4	4	4
4	4	4	4	4	3	4	4	5	4
5	3	4	4	4	4	3	4	4	3
4	4	4	4	4	4	4	4	4	3
5	3	4	3	1	2	3	4	3	5
3	2	2	3	2	2	3	2	2	3
3	4	4	4	5	5	4	5	5	4
3	3	4	4	4	4	4	3	5	4
5	3	4	5	5	3	3	4	4	4
4	3	4	4	3	4	5	4	4	5
5	3	3	4	5	4	3	5	4	4
5	4	3	3	4	5	4	4	4	4
3	3	5	5	5	4	5	5	5	5
3	3	5	4	4	4	3	4	5	4
5	4	4	4	3	5	3	4	3	4
4	5	5	3	5	4	3	4	4	4
4	5	3	3	5	3	4	5	5	4
4	4	4	5	3	4	2	2	1	1
4	4	4	4	5	3	4	4	4	3
3	4	5	3	5	4	5	4	5	4
4	5	4	4	5	5	5	4	4	5
4	4	5	4	4	3	4	4	5	3
2	2	2	2	3	2	3	2	2	2
2	1	1	1	3	2	3	2	1	2
4	5	4	4	4	4	4	4	4	3
4	5	3	5	5	5	3	4	5	4
5	4	4	3	3	4	5	4	3	4
3	5	4	5	4	5	4	5	4	3
4	4	4	5	1	2	3	4	5	5
4	4	3	5	5	5	4	4	5	4
2	1	1	3	3	2	1	2	2	1
2	2	1	2	2	1	2	2	1	2
4	3	4	5	4	3	4	5	5	4
3	5	5	4	5	4	4	5	3	5
1	2	1	2	4	4	4	4	5	4
5	3	4	4	4	3	5	3	4	4
2	1	2	2	2	2	2	2	2	3
4	4	4	3	3	3	4	4	5	4
3	5	4	4	3	5	4	4	5	5
1	1	2	2	2	1	1	3	2	2
4	4	3	3	2	1	3	5	4	5
5	5	3	4	3	3	5	5	4	3
3	4	4	5	4	4	5	5	3	4
4	5	5	5	2	2	4	4	3	4
5	5	4	3	3	4	4	5	3	4
3	2	2	2	3	3	2	3	2	1
4	4	5	3	4	4	4	4	4	4

4	3	5	3	4	4	5	5	5	4
4	4	3	4	5	4	4	4	4	3
5	3	5	4	5	5	4	3	3	5
3	3	4	4	3	3	5	4	3	4
2	2	1	2	2	3	3	2	2	3
3	4	4	3	4	4	4	4	5	4
4	4	5	3	5	4	3	5	3	4
5	4	5	4	5	3	4	4	5	5
5	5	5	4	4	5	5	5	3	3
4	5	4	3	3	5	4	5	4	3
4	4	4	5	3	5	5	5	4	4
4	5	4	4	4	3	5	5	5	4
5	4	5	4	3	3	2	1	1	2
2	3	2	1	3	1	2	2	2	3
1	2	3	2	2	1	2	2	3	3
3	5	5	5	5	3	5	3	4	5
5	4	4	5	4	5	5	4	4	5
5	4	4	4	3	3	3	3	5	4
4	4	5	4	4	3	4	4	4	3
4	4	4	3	3	3	4	4	4	4
3	4	4	5	4	4	4	5	4	4
4	4	5	3	4	3	3	3	4	4
3	4	5	4	3	5	5	4	3	3
5	4	4	4	3	3	3	5	4	3
3	4	4	3	5	4	2	1	1	2
4	4	3	4	4	4	4	4	5	4
4	5	5	4	4	3	4	4	4	4
4	4	4	5	5	3	5	4	3	3
4	5	4	5	4	5	4	4	4	3
1	2	2	2	1	2	2	2	2	1
5	5	3	4	4	3	3	4	4	4
4	5	3	4	4	4	4	5	4	5
4	3	3	4	4	4	3	5	5	4
4	5	4	5	5	3	4	5	5	4
5	4	5	4	4	3	4	4	5	3
4	4	5	4	3	4	5	5	3	3
4	3	4	4	3	4	2	2	1	2
5	4	3	3	4	4	4	3	4	5
4	3	4	5	3	3	3	4	5	4
3	4	3	3	5	3	4	5	4	5
2	2	2	2	4	4	4	3	5	4
3	3	4	5	5	3	1	1	2	1
3	3	2	1	3	3	2	1	2	2
1	2	2	2	1	1	2	2	2	1
3	5	4	4	2	1	4	4	4	4
1	2	2	3	2	2	2	2	1	2
4	4	4	5	5	5	4	3	5	4

5	3	4	4	5	3	3	4	5	5
4	4	4	3	4	5	3	4	4	3
3	4	5	4	3	3	4	4	4	4
3	2	2	3	1	3	1	2	2	2
1	1	2	1	5	3	3	5	4	5
4	4	4	3	5	4	3	4	5	5
3	3	3	3	4	3	4	3	5	3
3	3	5	5	4	4	4	4	4	4
5	4	4	3	4	4	5	4	3	3
5	5	4	4	4	4	4	5	4	5
4	4	5	3	5	3	5	4	4	3
3	4	3	4	4	3	5	3	3	3
4	5	4	4	5	5	4	4	3	4
4	3	3	5	4	4	5	4	5	3
2	2	1	1	4	4	5	4	4	4
1	1	1	1	5	3	3	5	5	4
4	3	4	4	3	3	5	3	4	4
4	4	4	4	3	4	5	3	3	5
5	4	4	4	3	5	3	4	3	4
4	4	3	4	3	3	5	4	4	4
4	4	3	4	3	5	3	3	4	4
3	2	3	3	2	3	2	3	3	3
4	4	5	3	4	4	4	3	5	4
3	4	4	5	4	4	3	4	5	4
2	1	2	2	4	4	4	4	5	3
1	2	3	1	1	2	1	2	2	2
3	5	5	4	4	4	4	4	4	4
2	2	2	2	2	2	3	2	1	3
5	3	5	5	3	3	4	4	5	4
4	5	4	4	3	4	3	4	3	5
3	4	5	5	3	5	3	4	4	4
3	4	4	3	3	5	4	3	3	4
4	4	4	4	3	4	3	4	4	4
3	4	4	4	4	5	5	4	5	4
5	4	3	3	5	4	1	2	2	1
2	1	3	2	2	3	1	3	1	3
4	4	3	5	3	5	5	3	4	4
5	4	4	3	3	3	4	3	3	4
3	3	3	4	5	5	5	4	3	5
4	4	4	4	4	5	5	5	5	3
2	2	1	2	1	1	3	1	2	3
3	4	3	5	3	4	5	4	5	4
4	5	4	5	4	4	3	4	4	4
5	4	5	4	4	4	3	5	5	3
4	3	3	4	4	4	3	4	4	4
3	5	5	5	5	3	1	2	1	2
4	4	3	4	5	5	4	5	4	3

3	1	2	1	3	2	3	2	1	1
4	4	5	5	4	5	4	4	3	5
5	3	5	4	3	5	4	5	3	4
3	3	4	3	4	5	4	4	4	3
5	3	4	4	5	3	3	4	4	5
3	3	4	4	4	5	5	4	3	5
5	3	4	4	4	5	5	3	4	4
3	3	4	5	1	2	4	4	3	5
3	4	5	3	4	4	4	4	4	4
4	4	4	4	3	4	3	4	4	3
5	3	3	3	4	5	5	4	4	4
4	5	5	4	5	4	4	4	5	4
4	4	4	4	3	5	3	5	4	3
4	5	4	5	3	4	3	4	3	5
3	3	5	3	5	4	5	4	4	4
4	4	5	5	4	5	4	4	4	5
4	4	4	4	4	4	3	5	5	4
4	4	4	3	4	5	5	4	4	4
3	5	4	4	4	5	4	5	3	4
4	3	5	3	5	4	4	5	3	4
4	4	3	3	5	4	4	4	5	4
3	3	4	3	3	3	4	3	5	5
5	4	5	5	4	4	4	3	4	4
2	1	2	1	3	2	2	2	1	1
5	5	4	4	5	4	5	4	3	4
5	4	3	3	4	4	4	3	5	3
3	3	2	1	2	2	3	2	3	2
3	4	4	4	3	5	3	3	4	5
4	4	5	4	4	3	4	5	4	3
3	2	3	2	3	2	2	2	1	3
4	4	5	5	3	4	4	4	4	3
4	4	4	4	4	3	4	4	4	4
5	4	4	3	4	4	5	5	4	5
4	4	4	4	3	3	3	4	3	4
3	5	3	5	4	4	3	3	3	3
5	4	4	4	4	4	3	4	5	5
4	5	5	3	3	5	3	5	4	4
3	4	5	4	4	5	4	4	5	5
4	3	4	3	3	3	4	4	3	4
3	5	4	5	5	3	5	3	4	4
4	4	5	5	3	3	4	4	3	3
1	1	3	3	2	1	2	3	1	2
5	4	3	3	5	4	5	3	5	5
4	4	4	4	3	3	5	4	4	4
3	3	3	4	4	4	3	5	5	4
4	5	4	3	5	4	3	3	3	3
4	3	5	3	5	4	4	5	4	4

4	4	3	4	5	4	4	5	3	3
4	5	3	3	3	4	3	4	3	5
4	5	3	3	5	4	4	3	4	4
4	5	3	4	5	4	4	4	5	3
3	4	4	4	4	5	5	5	4	4
4	4	5	5	4	4	4	4	4	4
3	4	4	4	4	4	4	5	5	3
4	4	4	5	4	5	5	5	3	3
1	1	3	2	2	3	1	2	2	2
5	4	4	4	3	4	5	3	4	4
5	5	3	3	3	3	5	3	4	3
3	4	4	3	3	3	5	3	4	5
5	4	4	4	4	3	4	4	4	4
3	4	3	3	4	4	3	4	5	4
4	3	3	3	5	4	5	5	4	5
4	4	5	4	5	4	5	3	3	5
4	3	5	4	5	3	5	3	5	5
5	5	4	3	4	4	5	5	3	5
4	4	5	5	3	3	4	4	3	3
5	4	5	4	3	3	4	5	5	5
2	2	2	2	3	3	2	2	2	2
5	4	3	5	5	5	4	4	4	5
4	5	5	4	3	3	4	3	4	3
1	1	1	1	4	4	4	4	4	4
4	4	4	5	4	5	3	4	4	5
3	4	3	3	4	3	4	4	3	4
4	5	3	5	3	4	1	1	2	1
4	3	3	5	4	5	5	4	4	4
4	4	3	4	5	5	3	5	3	4
3	3	5	3	4	3	4	4	4	4
4	4	5	5	3	3	1	2	1	1
3	3	5	5	1	2	3	4	3	4
3	4	4	4	5	4	4	4	5	4
5	5	5	4	4	5	4	3	5	3
4	4	4	4	4	3	5	5	4	4
1	3	3	1	3	3	3	3	1	1
5	3	3	4	5	4	4	3	4	4
5	5	5	3	4	3	4	4	5	3
4	3	3	4	2	1	3	4	4	4
4	4	4	4	4	4	4	4	4	4
5	4	4	5	4	4	4	5	4	4
4	4	3	3	4	4	4	3	3	4
4	4	4	4	3	3	3	5	4	4
5	4	3	4	4	4	3	4	5	4
3	3	3	4	4	3	4	4	5	4
3	2	2	2	2	2	3	1	2	3
4	4	3	5	2	1	3	4	4	5

2	1	2	1	4	3	4	3	4	4
5	4	4	5	4	5	4	3	5	4
1	1	2	2	3	4	4	4	4	5
2	3	2	2	2	2	3	2	2	2
5	4	3	3	3	4	1	2	2	2
5	3	3	3	3	4	5	4	3	4
4	3	4	4	5	3	4	4	5	4
4	4	3	4	5	4	5	4	4	5
4	5	5	4	4	5	4	5	4	3
5	3	3	5	4	5	4	5	3	4
5	4	3	3	3	5	5	4	4	4
5	3	3	4	5	5	4	5	5	4
4	4	3	4	3	5	4	3	4	4
4	4	4	5	3	3	4	4	3	3
5	3	4	4	4	5	4	5	4	4
4	3	5	4	4	4	4	4	5	4
4	4	4	3	3	3	4	3	4	4
3	4	3	4	5	4	2	1	2	1
3	4	4	3	3	3	4	4	3	3
5	5	5	5	4	4	4	5	3	3
4	3	5	4	2	1	5	4	4	5
4	5	4	5	5	4	4	4	4	5
3	2	3	2	2	2	2	2	1	2
2	2	1	1	2	2	3	1	1	2
4	3	4	4	4	4	4	4	4	5
4	4	3	3	5	4	5	4	5	4
5	4	4	4	4	3	3	4	3	4
5	3	4	4	4	5	4	5	4	4
4	3	4	3	4	5	3	4	4	5
3	3	4	5	4	3	5	4	3	5
3	1	2	2	2	2	2	1	1	2
4	4	4	3	3	3	4	5	5	5
2	2	1	1	5	4	3	5	4	4
3	3	4	5	4	4	5	4	4	5
3	2	2	2	2	3	1	1	3	3
4	4	4	4	3	4	4	5	4	4
4	4	4	5	4	4	4	5	4	3
5	5	3	4	4	4	3	4	4	4
4	5	4	4	5	4	3	3	4	5
2	1	1	1	4	4	3	4	3	4
5	4	4	4	3	5	3	5	3	4
1	3	1	1	2	2	2	2	1	2
3	2	1	2	3	3	2	3	3	3
2	2	2	1	2	2	2	1	2	2
4	4	4	4	3	3	4	3	3	5
3	5	4	4	5	5	3	5	4	3
3	3	4	4	5	5	4	5	3	3

5	3	4	5	3	4	1	1	2	1
5	5	5	5	1	1	5	5	4	5
2	2	1	2	3	2	3	3	2	2
4	4	4	4	4	3	3	4	5	3
3	4	4	3	4	4	4	5	4	3
3	4	5	4	4	5	3	3	4	5
3	4	5	4	4	4	4	4	5	5
4	4	5	4	4	3	4	4	4	4
4	4	3	4	1	1	4	4	4	4
2	3	3	2	2	3	1	2	3	2
4	5	4	4	3	4	4	5	4	4
5	4	5	4	3	4	4	3	4	5
2	2	3	1	2	2	2	2	1	3
4	3	4	4	4	4	1	1	1	2
3	4	5	4	3	3	1	1	1	2
3	1	2	1	2	2	2	2	2	3
5	5	5	4	5	5	2	1	2	1
5	3	5	4	4	4	3	4	4	4
4	4	5	3	5	4	4	5	5	4
5	3	3	3	4	4	5	4	4	4
5	4	4	3	3	4	5	4	5	4
4	4	5	4	4	3	3	4	3	5
4	4	4	3	3	4	3	5	3	5
5	3	4	4	4	5	5	4	4	4
4	5	3	3	3	4	3	3	3	4
2	3	2	3	1	2	2	1	2	2
4	5	4	4	4	3	4	4	4	4
4	5	5	4	3	4	3	4	5	5
3	4	3	5	3	4	3	5	4	3
4	5	3	3	5	3	4	4	3	5
3	3	5	4	3	4	4	3	4	3
5	5	4	4	5	5	4	4	4	4
5	5	5	5	5	3	4	3	3	4
3	5	3	5	5	3	4	3	3	4
5	4	5	3	3	4	5	5	4	4
3	5	5	3	3	5	3	4	3	4
3	5	5	4	3	3	5	3	3	4
3	5	4	4	1	1	3	5	5	4
4	4	5	4	2	2	3	3	5	4
4	4	4	3	5	3	3	4	4	3
4	4	4	4	4	3	4	5	3	5
5	5	5	5	3	5	4	4	4	3
5	5	3	4	3	4	2	1	1	2
5	4	3	3	3	5	4	5	3	5
4	4	3	4	4	4	4	3	4	4
5	4	3	5	4	3	3	4	5	5
4	4	5	3	5	4	3	4	4	5

3	4	4	4	4	3	5	4	5	5
4	4	3	4	4	3	4	3	4	5
3	4	4	5	3	5	3	4	3	4
4	4	3	5	4	3	4	3	5	5
5	5	3	5	4	3	4	3	5	5
3	3	4	5	4	5	3	5	3	4
3	2	1	2	1	2	2	2	2	2
1	2	1	2	4	4	5	3	4	4
1	1	2	1	3	3	4	3	5	4
4	5	4	4	5	4	5	4	5	3
4	3	3	3	4	5	5	4	4	5
4	5	3	4	3	3	5	5	3	4
1	3	2	2	2	1	2	3	1	2
5	5	4	4	4	3	4	4	4	5
4	4	4	3	4	5	5	5	4	4
5	5	4	3	3	3	3	4	5	3
4	5	4	3	4	5	5	4	4	3
3	4	4	3	3	4	4	5	4	3
5	4	3	4	2	2	4	4	5	4
3	5	4	5	4	3	3	4	4	4
4	4	5	5	4	4	4	5	4	4
1	3	2	2	2	3	1	2	2	2
3	3	4	4	4	4	5	4	3	4
5	4	4	3	3	4	4	4	5	3
3	3	3	3	3	2	2	2	1	3
2	2	1	1	3	2	2	2	1	2
4	4	4	5	4	3	2	1	2	1
5	4	4	5	5	4	4	4	4	5
3	4	3	5	4	4	4	4	4	3
4	3	4	3	5	4	4	5	5	5
4	3	4	3	4	4	4	4	5	5
5	4	4	4	3	5	5	3	4	4
4	3	3	5	4	4	4	4	5	4
3	4	5	5	4	4	4	4	3	5
3	5	4	4	4	4	4	3	5	4
4	3	5	3	4	4	3	3	4	3
3	4	4	3	4	4	3	5	3	4
4	3	5	4	3	3	4	4	4	4
3	4	3	3	5	4	4	5	4	3
5	5	4	4	5	4	4	4	4	3
5	4	5	5	4	3	4	4	4	5
3	5	4	4	3	3	1	1	1	2
2	2	1	2	5	3	4	4	5	3
3	3	3	2	3	1	3	2	1	3
4	4	4	5	4	4	3	4	3	4
4	4	4	5	5	4	5	3	3	3
4	4	5	5	3	4	5	4	4	4

1	1	1	1	5	3	3	4	4	3
5	4	3	4	4	5	3	4	4	5
4	4	4	4	3	3	4	4	4	3
3	5	5	4	4	5	3	3	5	4
5	5	4	5	4	4	4	4	5	5
5	4	4	4	4	4	4	5	3	5
5	3	5	3	5	3	3	5	5	5
1	1	3	1	1	2	1	1	2	2
4	5	4	3	4	4	4	5	3	3
3	3	4	3	4	4	5	4	3	4
4	3	4	5	5	3	4	5	4	4
5	4	3	4	1	2	4	4	3	4
5	3	3	5	5	5	4	4	5	4
5	4	4	3	5	3	5	3	3	4
3	3	2	3	3	3	2	3	1	1
2	2	2	2	3	2	2	3	3	2
1	1	2	2	2	2	1	2	2	1
3	4	4	5	3	4	4	3	3	4
5	4	5	5	5	4	3	3	4	5
3	3	5	3	3	4	3	3	3	4
3	4	4	3	3	5	4	3	4	4
5	5	4	4	5	4	4	4	4	3
5	4	5	5	4	3	4	4	4	5
3	5	4	4	3	3	1	1	1	2
2	2	1	2	5	3	4	4	5	3
3	3	3	2	3	1	3	2	1	3
4	4	4	5	4	4	3	4	3	4
4	4	4	5	5	4	5	3	3	3
4	4	5	5	3	4	5	4	4	4
1	1	1	1	5	3	3	4	4	3
5	4	3	4	4	5	3	4	4	5
4	4	4	4	3	3	4	4	4	3
3	5	5	4	4	5	3	3	5	4
5	5	4	5	4	4	4	4	5	5
5	4	4	4	4	4	4	5	3	5
5	3	5	3	5	3	3	5	5	5
1	1	3	1	1	2	1	1	2	2
4	5	4	3	4	4	4	5	3	3
3	3	4	3	4	4	5	4	3	4
4	3	4	5	5	3	4	5	4	4
5	4	3	4	1	2	4	4	3	4
5	3	3	5	5	5	4	4	5	4
5	4	4	3	5	3	5	3	3	4
3	3	2	3	3	3	2	3	1	1
2	2	2	2	3	2	2	3	3	2
1	1	2	2	2	2	1	2	2	1
3	4	4	5	3	4	4	3	3	4

5	4	5	5	5	4	3	3	4	5
3	3	5	3	3	4	3	3	3	4
3	4	4	3	3	5	4	3	4	4
5	5	4	4	5	4	4	4	4	3
5	4	5	5	4	3	4	4	4	5
3	5	4	4	3	3	1	1	1	2
2	2	1	2	5	3	4	4	5	3
3	3	3	2	3	1	3	2	1	3
4	4	4	5	4	4	3	4	3	4
4	4	4	5	5	4	5	3	3	3
4	4	5	5	3	4	5	4	4	4
1	1	1	1	5	3	3	4	4	3
5	4	3	4	4	5	3	4	4	5
4	4	4	4	3	3	4	4	4	3
3	5	5	4	4	5	3	3	5	4
5	5	4	5	4	4	4	4	5	5
5	4	4	4	4	4	4	5	3	5
5	3	5	3	5	3	3	5	5	5
1	1	3	1	1	2	1	1	2	2
4	5	4	3	4	4	4	5	3	3
3	3	4	3	4	4	5	4	3	4
4	3	4	5	5	3	4	5	4	4
5	4	3	4	1	2	4	4	3	4
5	3	3	5	5	5	4	4	5	4
5	4	4	3	5	3	5	3	3	4
3	3	2	3	3	3	2	3	1	1
2	2	2	2	3	2	2	3	3	2
1	1	2	2	2	2	1	2	2	1
3	4	4	5	3	4	4	3	3	4
5	4	5	5	5	4	3	3	4	5
3	3	5	3	3	4	3	3	3	4
3	4	4	3	3	5	4	3	4	4
5	5	4	4	5	4	4	4	4	3
5	4	5	5	4	3	4	4	4	5
3	5	4	4	3	3	1	1	1	2
2	2	1	2	5	3	4	4	5	3
3	3	3	2	3	1	3	2	1	3
4	4	4	5	4	4	3	4	3	4
4	4	4	5	5	4	5	3	3	3
4	4	5	5	3	4	5	4	4	4
1	1	1	1	5	3	3	4	4	3
5	4	3	4	4	5	3	4	4	5
4	4	4	4	3	3	4	4	4	3
3	5	5	4	4	5	3	3	5	4
5	5	4	5	4	4	4	4	5	5
5	4	4	4	4	4	4	5	3	5
5	3	5	3	5	3	3	5	5	5

1	1	3	1	1	2	1	1	2	2
4	5	4	3	4	4	4	5	3	3
3	3	4	3	4	4	5	4	3	4
4	3	4	5	5	3	4	5	4	4
5	4	3	4	1	2	4	4	3	4
5	3	3	5	5	5	4	4	5	4
5	4	4	3	5	3	5	3	3	4
3	3	2	3	3	3	2	3	1	1
2	2	2	2	3	2	2	3	3	2
1	1	2	2	2	2	1	2	2	1
3	4	4	5	3	4	4	3	3	4
5	4	5	5	5	4	3	3	4	5
3	3	5	3	3	4	3	3	3	4
3	4	4	3	3	5	4	3	4	4
3	3	2	3	3	3	2	3	1	1
2	2	2	2	3	2	2	3	3	2
1	1	2	2	2	2	1	2	2	1
3	4	4	5	3	4	4	3	3	4
5	4	5	5	5	4	3	3	4	5
3	3	5	3	3	4	3	3	3	4
3	4	4	3	3	5	4	3	4	4
3	3	2	3	3	3	2	3	1	1
2	2	2	2	3	2	2	3	3	2
1	1	2	2	2	2	1	2	2	1
3	4	4	5	3	4	4	3	3	4
1	1	2	2	2	2	1	2	2	1

IAS_12	IAS_13	IAS_14	IAS_15	MAAS_1	MAAS_2	MAAS_3	MAAS_4	MAAS_5	MAAS_6
4	4	4	4	5	4	3	3	3	5
1	2	4	4	4	4	4	4	5	3
5	4	4	5	3	4	4	4	5	4
4	4	3	4	4	5	3	3	4	4
3	4	4	4	4	5	4	4	5	4
3	5	4	5	4	4	3	4	5	5
4	4	4	3	5	3	4	3	5	3
3	5	3	4	4	4	4	5	5	5
5	3	3	5	1	2	1	2	2	1
4	3	4	5	4	4	4	5	5	4
4	4	4	3	3	4	4	4	5	5
4	4	4	4	4	3	4	4	3	4
5	4	5	3	4	3	3	3	3	4
5	4	5	4	4	4	3	5	5	4
3	2	3	2	3	2	2	3	2	2
4	3	4	4	5	4	4	4	3	4
3	2	1	2	2	3	2	2	1	3
5	5	4	4	3	4	3	4	4	4
3	4	4	4	3	4	5	4	4	5
4	4	4	4	3	4	4	4	5	4
4	4	4	4	4	5	3	4	4	4
4	3	4	4	5	3	5	5	3	4
3	2	2	1	3	2	3	2	3	2
3	5	3	4	4	3	4	4	3	3
5	4	4	5	5	3	5	4	3	4
4	4	2	1	5	4	3	5	4	4
4	4	4	4	4	4	3	4	3	4
3	4	5	4	4	4	5	4	4	5
2	2	1	3	3	1	2	1	2	1
2	2	2	2	2	3	2	3	1	1
4	3	2	2	4	4	4	5	4	5
4	4	4	5	4	4	4	4	5	5
5	3	5	3	4	4	3	3	3	4
1	1	5	4	5	4	4	3	5	4
3	4	4	4	4	4	4	3	3	3
5	4	4	4	3	3	5	5	3	4
4	3	5	3	4	4	5	4	3	4
4	3	4	4	4	4	5	5	4	5
4	5	3	4	4	3	5	5	5	4
5	3	4	4	3	3	3	5	4	3
4	4	4	5	4	4	5	4	4	4
4	3	5	5	4	4	4	4	4	4
4	4	4	3	3	4	3	3	5	4
3	5	4	3	5	4	5	5	3	3
3	4	4	4	3	4	3	4	4	4
3	1	2	1	3	1	2	3	3	1

3	4	3	5	5	5	4	4	4	3
2	2	1	2	2	1	1	2	2	2
3	3	4	4	3	4	4	3	3	4
3	3	3	5	4	3	4	3	4	4
4	3	4	4	4	3	3	4	3	4
3	4	3	5	5	5	3	4	4	4
3	3	3	4	3	4	3	4	4	5
1	3	2	2	2	1	2	2	3	2
3	4	5	3	4	4	3	3	4	3
4	5	3	4	3	4	4	4	3	5
2	2	2	2	2	3	2	1	3	2
5	4	4	4	3	5	4	4	4	4
3	4	4	3	4	4	4	5	3	4
5	5	3	5	5	4	3	4	4	5
4	3	5	5	1	2	2	2	1	1
3	4	4	4	3	4	4	3	3	5
4	5	4	4	5	4	4	4	4	3
5	4	5	4	4	5	3	3	4	5
3	5	5	4	4	5	3	5	5	3
4	5	4	4	4	4	4	5	3	5
4	4	4	3	3	4	5	4	4	3
3	3	4	3	4	5	3	3	3	3
3	5	1	2	5	3	4	3	4	4
2	2	2	1	1	1	2	2	1	2
5	5	3	3	4	4	3	3	4	4
3	4	4	3	3	4	4	3	4	4
5	4	4	3	4	3	4	4	3	3
3	5	4	4	5	4	5	4	4	5
3	3	3	3	3	3	5	4	5	3
4	4	3	3	3	4	3	5	4	4
5	4	5	4	5	3	4	4	4	3
4	5	5	3	1	1	1	2	1	1
5	4	4	4	4	4	4	3	3	4
4	4	4	5	4	3	3	5	4	4
5	5	4	3	4	4	3	4	4	3
2	1	4	5	4	3	3	4	3	4
4	5	5	4	4	3	3	4	5	4
4	5	4	4	4	4	4	3	3	5
4	4	4	4	5	4	3	4	4	3
5	5	4	4	4	5	4	4	3	5
2	2	1	2	3	1	3	3	2	1
2	2	1	3	2	2	1	2	2	1
3	4	3	4	4	5	5	3	4	4
4	3	5	5	4	4	4	3	4	4
3	4	3	3	5	5	3	4	3	3
3	5	4	4	4	4	3	5	3	5
3	3	1	2	4	4	4	4	4	3

5	5	5	3	4	3	3	4	5	3
2	3	3	1	3	1	2	2	1	2
3	1	3	1	2	1	2	3	2	2
4	4	4	4	4	3	4	5	4	4
3	3	5	4	4	4	5	4	5	4
4	4	4	5	4	3	4	4	4	3
3	4	4	3	5	4	5	4	5	5
1	2	3	3	2	2	2	2	1	2
3	5	4	5	4	4	4	4	4	3
4	4	4	4	4	3	4	5	3	4
2	2	2	1	1	3	2	2	2	2
4	4	1	1	4	4	4	4	4	4
3	4	4	5	4	3	5	5	4	4
5	4	4	5	3	3	4	4	3	4
3	3	2	2	5	3	4	4	4	4
4	5	4	4	3	3	3	4	4	5
1	2	2	3	1	1	2	3	3	2
3	4	4	4	5	4	3	4	3	4
4	5	4	4	4	4	4	4	4	5
3	3	4	5	4	4	4	3	3	5
4	5	3	4	5	3	3	4	4	4
4	5	4	3	4	3	3	3	4	4
2	2	1	3	2	2	1	3	1	2
3	4	3	4	4	4	4	3	4	3
3	4	3	5	3	4	4	5	4	4
3	5	3	4	4	4	3	4	4	3
3	5	3	5	5	4	3	5	4	5
4	5	4	4	5	4	5	4	5	5
5	3	3	4	4	5	5	4	4	4
3	5	4	5	4	4	4	3	4	4
1	1	4	3	4	4	4	3	3	3
3	1	1	2	2	1	2	2	3	2
3	1	2	2	3	1	1	2	2	2
5	5	5	4	3	4	5	3	4	4
4	4	3	5	4	3	4	3	3	3
4	5	4	5	4	5	5	4	3	4
4	5	4	3	5	4	4	4	3	3
4	4	4	4	4	3	4	3	5	3
4	4	4	3	4	4	4	5	3	4
4	5	5	4	5	5	5	4	4	3
3	4	5	5	4	3	3	5	4	3
5	4	4	4	4	3	5	4	4	5
1	2	3	4	4	3	4	4	4	5
3	4	4	4	3	4	3	4	4	4
4	3	5	4	4	5	3	5	4	5
3	5	3	4	5	4	4	5	3	3
4	3	5	3	4	3	3	3	4	4

1	2	2	3	3	3	2	3	2	2
4	3	4	4	4	4	4	3	4	4
5	4	4	3	4	3	3	5	4	4
4	3	4	5	4	5	3	5	4	3
4	3	4	3	5	5	4	4	4	5
4	4	5	4	4	4	4	5	5	3
4	3	3	5	4	3	4	4	4	4
2	2	5	4	1	2	1	1	2	2
4	4	3	5	5	4	4	4	3	4
4	4	4	5	3	4	4	5	4	3
4	4	4	3	5	3	3	3	3	3
3	4	3	4	4	5	4	4	3	5
2	1	5	5	4	3	4	5	4	3
2	2	1	2	3	3	3	2	2	1
3	3	1	3	3	1	1	2	2	2
3	4	2	2	3	3	5	4	4	4
2	3	1	2	3	3	1	1	1	2
4	4	4	5	4	5	5	5	5	5
4	5	3	4	3	4	5	5	5	3
5	5	3	5	5	4	4	4	5	3
5	4	4	3	4	4	4	4	5	5
1	3	2	2	2	2	1	3	3	1
4	4	3	5	4	4	3	4	5	4
3	4	4	3	5	5	4	3	3	3
4	3	4	4	5	4	3	4	5	5
4	4	5	5	4	3	4	3	5	4
4	4	3	5	4	4	5	4	4	4
3	4	3	5	4	4	3	4	4	4
5	5	3	3	4	4	5	3	4	4
4	4	5	4	4	3	5	3	4	5
4	3	4	4	2	1	1	1	2	1
4	3	4	5	3	5	4	5	5	5
3	5	5	5	4	3	4	3	5	4
4	4	4	3	4	3	5	4	4	3
4	3	3	4	3	4	3	4	4	5
4	5	4	4	3	4	4	4	4	5
4	4	4	3	4	4	5	4	5	5
4	3	5	3	4	3	5	5	4	5
5	4	4	5	4	4	4	4	4	4
1	2	1	3	2	1	1	2	3	1
5	4	4	3	3	5	5	3	4	4
5	5	5	5	4	3	3	3	4	4
4	3	5	4	5	3	4	5	4	4
2	2	2	2	2	3	2	3	3	2
4	5	3	4	2	2	1	1	1	1
2	1	2	1	1	2	2	2	1	2
4	4	4	4	3	4	5	5	5	4

4	5	4	5	4	3	4	4	4	4
5	4	5	5	4	4	4	4	5	4
4	4	4	4	5	3	3	4	4	5
4	3	4	4	5	5	4	3	3	4
3	4	4	3	4	5	3	3	5	5
2	2	5	4	4	4	4	5	4	5
2	3	1	1	1	3	2	2	1	2
3	4	5	3	4	4	5	4	4	4
4	4	4	5	5	4	5	4	5	5
5	4	4	4	4	4	4	5	4	4
3	5	5	4	3	4	3	3	4	4
3	1	2	1	2	2	2	2	2	1
4	3	4	4	3	5	4	5	5	4
5	4	5	4	3	4	5	4	3	4
3	3	3	4	5	4	4	3	5	4
4	3	3	4	3	3	4	3	5	3
1	2	5	4	4	4	4	4	5	4
5	4	5	5	3	3	4	3	3	4
2	2	3	3	2	2	1	2	2	2
4	4	4	5	4	3	4	4	5	4
4	3	4	3	3	3	4	4	4	5
5	4	4	5	4	4	4	4	5	4
4	3	4	4	3	3	4	5	3	4
3	4	5	4	4	5	3	4	4	4
5	4	4	4	4	4	4	3	4	4
5	4	1	2	4	3	4	5	3	5
3	3	4	5	3	5	5	5	4	4
3	4	3	4	4	5	4	3	4	3
4	4	3	4	5	5	5	4	4	5
4	4	3	5	5	5	4	5	3	5
4	3	3	5	3	3	5	5	4	4
4	4	4	5	3	5	4	3	4	4
4	3	5	4	5	3	3	4	5	3
4	4	4	3	3	5	3	4	3	5
4	5	3	4	4	4	3	3	5	4
4	5	3	5	4	4	5	3	3	4
5	4	4	4	3	3	5	3	4	4
4	4	3	4	5	4	4	5	5	5
5	5	4	3	4	3	4	3	5	3
3	4	4	3	5	5	4	3	4	4
4	3	5	4	5	4	4	4	4	4
2	2	3	3	1	2	1	2	3	2
4	5	4	4	3	3	4	5	4	5
5	3	3	5	4	3	3	4	4	4
2	3	3	2	3	1	3	2	2	2
3	4	4	3	4	5	5	3	4	3
4	4	4	4	4	3	4	5	4	5

3	2	3	2	2	3	1	2	2	3
4	5	5	5	5	4	4	5	3	3
5	5	5	5	4	5	4	4	4	4
3	3	4	5	1	1	2	2	1	1
4	4	4	3	1	2	1	2	1	2
5	4	4	4	3	5	4	5	3	5
4	4	4	3	5	4	3	4	4	4
3	4	3	4	4	4	4	4	5	5
4	4	4	5	4	4	4	4	4	4
3	5	3	5	5	4	3	4	4	3
3	4	3	4	4	4	4	4	3	3
4	4	5	4	4	3	3	3	4	5
1	3	2	2	3	3	2	2	1	1
4	3	4	5	4	4	3	4	3	5
5	3	3	4	3	4	4	3	3	4
4	5	5	5	4	4	4	4	3	4
4	4	5	4	4	5	3	5	4	3
3	5	4	4	2	1	1	2	1	2
3	5	4	4	4	5	4	4	5	4
3	4	5	4	4	4	4	3	4	3
5	5	3	4	3	4	4	4	3	4
4	3	3	3	3	4	3	3	4	5
4	5	4	5	4	3	4	3	4	4
3	5	4	3	3	3	4	4	5	4
5	3	5	5	4	4	4	4	4	4
4	5	5	4	4	5	4	4	4	3
2	1	3	2	2	2	2	1	3	1
4	3	4	4	3	4	4	3	5	4
5	4	3	4	5	4	4	4	4	5
4	5	4	4	5	5	5	5	5	4
3	4	4	4	5	5	4	5	5	3
5	4	5	3	4	3	5	4	4	3
5	4	3	4	2	2	2	2	2	1
3	3	4	4	4	4	4	3	5	3
3	3	5	4	3	4	5	4	3	4
4	4	4	4	5	5	3	4	4	4
5	4	4	3	4	4	5	5	3	3
4	5	5	4	3	3	4	4	5	4
3	2	1	3	1	3	3	1	2	2
4	4	3	4	4	3	4	3	4	4
5	4	4	4	5	4	5	3	4	4
5	5	4	4	4	4	5	5	5	3
4	5	5	4	5	4	4	5	4	3
4	5	4	3	4	5	4	4	3	4
2	1	3	4	4	4	4	4	3	4
3	4	4	5	4	4	4	4	4	4
4	3	5	5	5	3	4	5	4	5

3	3	5	3	5	4	4	4	3	3
1	2	3	5	3	4	4	3	4	4
3	5	1	1	4	4	3	3	4	5
4	4	3	5	5	3	4	4	5	5
4	4	4	4	5	3	4	5	4	4
4	3	4	3	2	2	1	2	1	1
3	2	2	3	1	3	2	2	2	2
3	4	5	5	4	4	4	3	5	4
4	4	4	5	3	3	4	5	5	4
3	5	2	2	3	3	3	5	4	5
5	4	4	5	1	2	2	2	1	2
3	4	4	4	3	3	3	4	4	5
3	3	4	5	4	3	3	4	4	4
5	5	3	5	3	4	4	3	4	4
5	3	5	3	1	2	2	1	1	2
5	4	5	4	4	4	5	3	4	5
2	1	3	2	2	3	1	2	2	2
5	5	2	2	5	4	4	4	4	5
4	3	3	4	3	5	3	5	3	4
4	4	5	5	5	4	3	5	3	5
3	5	5	3	4	5	4	5	4	5
2	2	3	2	2	3	2	3	2	3
2	2	3	4	4	4	3	4	4	3
4	4	3	5	1	1	1	2	2	1
4	3	4	3	3	5	4	3	5	4
4	3	5	4	4	4	4	5	5	4
5	4	3	3	4	3	4	4	4	3
4	5	4	3	4	3	4	3	4	5
3	4	5	5	3	5	4	4	3	5
4	3	4	4	3	4	4	5	5	5
3	4	5	3	3	3	4	4	4	3
5	4	4	4	5	4	3	5	4	4
3	3	4	4	4	4	5	5	3	4
3	4	4	4	5	3	5	4	3	4
4	3	4	3	4	5	4	5	3	5
2	2	4	5	3	4	3	5	4	5
5	4	5	3	3	3	4	3	4	5
3	4	3	4	4	4	4	5	5	3
4	4	1	1	4	5	3	4	4	3
4	5	5	4	4	4	4	5	3	5
1	2	1	2	2	2	2	3	2	2
3	2	1	2	1	2	3	2	2	2
3	3	3	4	3	4	3	3	3	4
3	4	4	4	4	4	4	3	4	4
5	3	4	4	4	3	4	5	3	4
5	5	4	3	5	4	3	4	3	3
4	3	5	4	3	3	3	4	4	4

3	4	5	5	5	3	3	4	4	3
2	2	2	3	2	1	2	1	2	2
3	4	5	4	4	5	4	5	4	4
4	3	3	4	4	3	4	3	5	5
4	4	5	4	5	3	4	4	3	5
2	2	2	1	2	2	1	3	3	1
4	5	3	4	4	4	4	4	4	4
4	3	4	5	4	3	4	4	3	5
4	3	3	3	3	4	3	4	5	5
3	4	5	4	4	3	5	5	4	3
4	3	5	5	4	4	5	4	3	3
4	5	4	5	4	4	4	4	4	3
2	1	3	1	3	2	2	3	3	2
2	3	1	1	1	1	2	3	1	1
1	1	3	2	2	3	2	3	2	1
4	5	4	4	4	4	4	4	3	4
5	5	4	3	4	5	4	4	4	4
4	4	3	4	4	5	4	3	4	3
2	1	3	5	4	4	4	4	4	4
5	5	2	1	3	4	4	4	3	5
2	1	1	1	2	2	2	2	3	2
5	3	3	4	3	4	4	4	3	5
4	4	4	4	3	4	3	3	5	4
5	3	4	4	5	3	3	4	4	4
5	4	5	5	4	4	4	3	4	5
4	4	5	3	4	3	4	3	4	4
4	4	1	2	4	4	3	3	5	5
2	3	2	2	2	2	2	2	2	3
4	3	4	5	3	5	5	4	3	5
3	3	3	4	3	4	5	3	5	5
3	2	3	2	1	1	3	2	3	2
1	2	4	4	4	4	3	5	5	4
1	1	5	4	5	5	4	4	5	4
2	2	2	1	2	2	3	3	1	2
1	2	3	3	5	4	5	3	4	4
3	3	5	3	4	4	3	4	4	3
4	4	3	4	3	3	4	4	4	3
3	4	4	4	3	4	5	3	3	5
5	4	5	5	4	5	4	3	4	3
4	5	5	4	3	4	4	4	4	4
4	4	3	4	5	4	5	4	5	3
4	4	4	4	3	3	4	4	3	4
4	4	5	3	4	5	5	4	4	5
3	2	2	2	2	2	3	3	2	2
5	5	5	4	2	1	2	1	1	1
3	4	4	5	4	4	3	4	4	3
4	3	4	4	3	3	5	4	5	4

4	5	3	5	5	4	4	5	4	5
3	4	3	4	4	5	4	4	3	5
4	3	4	5	3	4	3	4	3	5
3	4	4	4	3	3	3	4	4	3
4	5	4	4	4	4	4	3	3	5
5	5	4	3	5	3	5	4	4	5
4	3	5	3	4	4	5	4	5	3
3	4	5	4	5	5	5	3	5	3
5	4	1	1	3	5	4	5	3	5
5	4	2	2	3	4	5	4	3	5
4	4	4	5	4	3	5	3	5	4
4	5	3	4	4	5	4	5	5	4
5	4	4	4	4	3	4	4	4	5
2	1	4	4	4	4	3	5	3	4
4	4	3	4	5	5	4	4	4	4
3	3	4	4	3	4	3	4	5	3
4	4	4	4	3	3	3	3	4	4
4	3	5	4	4	4	5	5	3	3
4	4	5	3	5	3	3	3	4	4
5	4	5	3	4	5	4	3	5	5
4	5	5	4	5	4	5	5	4	3
4	4	4	4	4	3	3	4	4	4
3	4	4	5	4	5	3	5	4	4
5	4	4	5	3	4	3	3	4	5
2	1	2	3	2	3	3	1	2	2
4	4	4	4	3	4	4	4	3	4
4	3	4	4	5	4	4	5	3	4
4	4	5	4	4	3	4	4	4	5
4	5	4	4	3	4	5	4	4	4
5	5	5	3	3	5	5	5	5	5
3	2	1	3	1	2	1	3	2	2
5	4	5	4	4	3	4	4	3	4
3	4	3	5	4	3	4	5	4	4
5	4	5	3	5	4	4	5	3	4
4	4	3	4	4	3	5	5	4	5
5	4	5	4	5	3	3	3	5	5
4	4	1	1	4	3	3	4	4	4
5	5	5	3	4	4	5	4	4	4
5	5	4	3	4	4	5	4	4	4
1	3	2	2	2	2	1	2	1	1
5	3	3	3	3	4	4	4	3	4
4	4	4	4	3	3	4	5	3	4
3	1	2	2	1	1	2	2	3	3
2	2	1	1	3	2	2	1	2	2
2	2	4	3	4	5	4	4	4	5
4	5	4	5	3	4	5	4	5	4
4	3	3	3	4	4	4	4	4	3

4	3	3	5	3	5	4	3	5	5
4	4	3	5	4	3	3	5	4	4
3	3	4	4	5	4	5	4	4	3
4	3	3	4	1	1	1	1	2	2
4	5	5	4	4	5	5	5	5	4
3	3	3	4	5	4	4	3	4	5
4	4	4	5	5	3	3	3	4	3
5	4	4	3	4	5	5	4	4	4
4	4	3	5	3	3	3	4	5	3
4	5	3	5	5	3	5	5	3	4
4	3	4	5	5	4	5	4	4	4
4	3	4	3	3	4	5	4	3	4
2	2	4	3	2	2	2	2	1	2
4	4	5	5	4	4	4	5	3	4
1	1	2	2	2	2	3	2	2	3
5	3	4	4	5	3	4	5	5	4
4	4	5	4	3	3	4	5	4	4
5	4	3	4	4	4	3	4	3	3
4	3	5	4	4	4	4	4	5	4
4	5	4	4	4	4	3	3	4	4
5	3	4	4	3	4	4	4	4	5
3	4	3	3	3	5	4	4	4	5
4	4	5	5	3	5	4	3	4	5
3	3	4	4	5	4	3	3	4	4
4	4	4	4	3	4	4	4	3	5
3	2	2	2	1	1	3	1	1	3
5	3	4	5	4	5	5	5	4	4
4	4	4	5	4	4	4	4	4	4
3	4	5	4	1	2	2	2	2	2
3	3	2	1	5	3	3	4	5	5
4	5	5	4	4	3	4	3	4	5
5	5	5	3	5	4	5	3	5	5
1	2	3	2	3	3	2	2	1	2
2	2	2	2	2	1	2	2	2	3
2	2	2	1	1	3	1	2	2	2
4	5	4	5	4	4	3	4	5	4
4	4	5	5	4	3	4	3	4	4
3	3	4	4	4	3	3	4	5	3
5	4	4	3	4	5	3	5	4	3
4	4	4	4	5	4	3	3	3	5
1	2	4	4	4	4	4	4	5	3
5	4	4	5	3	4	4	4	5	4
4	4	3	4	4	5	3	3	4	4
3	4	4	4	4	5	4	4	5	4
3	5	4	5	4	4	3	4	5	5
4	4	4	3	5	3	4	3	5	3
3	5	3	4	4	4	4	5	5	5

5	3	3	5	1	2	1	2	2	1
4	3	4	5	4	4	4	5	5	4
4	4	4	3	3	4	4	4	5	5
4	4	4	4	4	3	4	4	3	4
5	4	5	3	4	3	3	3	3	4
5	4	5	4	4	4	3	5	5	4
3	2	3	2	3	2	2	3	2	2
4	3	4	4	5	4	4	4	3	4
3	2	1	2	2	3	2	2	1	3
5	5	4	4	3	4	3	4	4	4
3	4	4	4	3	4	5	4	4	5
4	4	4	4	3	4	4	4	5	4
4	4	4	4	4	5	3	4	4	4
4	3	4	4	5	3	5	5	3	4
3	2	2	1	3	2	3	2	3	2
3	5	3	4	4	3	4	4	3	3
5	4	4	5	5	3	5	4	3	4
4	4	2	1	5	4	3	5	4	4
4	4	4	4	4	4	3	4	3	4
3	4	5	4	4	4	5	4	4	5
2	2	1	3	3	1	2	1	2	1
2	2	2	2	2	3	2	3	1	1
4	3	2	2	4	4	4	5	4	5
4	4	4	5	4	4	4	4	5	5
5	3	5	3	4	4	3	3	3	4
1	1	5	4	5	4	4	3	5	4
3	4	4	4	4	4	4	3	3	3
5	4	4	4	3	3	5	5	3	4
4	3	5	3	4	4	5	4	3	4
4	3	4	4	4	4	5	5	4	5
4	5	3	4	4	3	5	5	5	4
5	3	4	4	3	3	3	5	4	3
4	4	4	5	4	4	5	4	4	4
4	3	5	5	4	4	4	4	4	4
4	4	4	3	3	4	3	3	5	4
3	5	4	3	5	4	5	5	3	3
3	4	4	4	3	4	3	4	4	4
3	1	2	1	3	1	2	3	3	1
3	4	3	5	5	5	4	4	4	3
2	2	1	2	2	1	1	2	2	2
3	3	4	4	3	4	4	3	3	4
3	3	3	5	4	3	4	3	4	4
4	3	4	4	4	3	3	4	3	4
3	4	3	5	5	5	3	4	4	4
3	3	3	4	3	4	3	4	4	5
1	3	2	2	2	1	2	2	3	2
3	4	5	3	4	4	3	3	4	3

4	5	3	4	3	4	4	4	3	5
2	2	2	2	2	3	2	1	3	2
5	4	4	4	3	5	4	4	4	4
3	4	4	3	4	4	4	5	3	4
5	5	3	5	5	4	3	4	4	5
4	3	5	5	1	2	2	2	1	1
3	4	4	4	3	4	4	3	3	5
4	5	4	4	5	4	4	4	4	3
5	4	5	4	4	5	3	3	4	5
3	5	5	4	4	5	3	5	5	3
4	5	4	4	4	4	4	5	3	5
4	4	4	3	3	4	5	4	4	3
3	3	4	3	4	5	3	3	3	3
3	5	1	2	5	3	4	3	4	4
2	2	2	1	1	1	2	2	1	2
5	5	3	3	4	4	3	3	4	4
3	4	4	3	3	4	4	3	4	4
5	4	4	3	4	3	4	4	3	3
3	5	4	4	5	4	5	4	4	5
3	3	3	3	3	3	5	4	5	3
4	4	3	3	3	4	3	5	4	4
5	4	5	4	5	3	4	4	4	3
4	5	5	3	1	1	1	2	1	1
5	4	4	4	4	4	4	3	3	4
4	4	4	5	4	3	3	5	4	4
5	5	4	3	4	4	3	4	4	3
2	1	4	5	4	3	3	4	3	4
4	5	5	4	4	3	3	4	5	4
4	5	4	4	4	4	4	3	3	5
4	4	4	4	5	4	3	4	4	3
5	5	4	4	4	5	4	4	3	5
2	2	1	2	3	1	3	3	2	1
2	2	1	3	2	2	1	2	2	1
3	4	3	4	4	5	5	3	4	4
4	3	5	5	4	4	4	3	4	4
3	4	3	3	5	5	3	4	3	3
3	5	4	4	4	4	3	5	3	5
3	3	1	2	4	4	4	4	4	3
5	5	5	3	4	3	3	4	5	3
2	3	3	1	3	1	2	2	1	2
3	1	3	1	2	1	2	3	2	2
4	4	4	4	4	3	4	5	4	4
3	3	5	4	4	4	5	4	5	4
4	4	4	5	4	3	4	4	4	3
3	4	4	3	5	4	5	4	5	5
1	2	3	3	2	2	2	2	1	2
3	5	4	5	4	4	4	4	4	3

4	4	4	4	4	3	4	5	3	4
2	2	2	1	1	3	2	2	2	2
4	4	1	1	4	4	4	4	4	4
3	4	4	5	4	3	5	5	4	4
5	4	4	5	3	3	4	4	3	4
3	3	2	2	5	3	4	4	4	4
4	5	4	4	3	3	3	4	4	5
1	2	2	3	1	1	2	3	3	2
3	4	4	4	5	4	3	4	3	4
4	5	4	4	4	4	4	4	4	5
3	3	4	5	4	4	4	3	3	5
4	5	3	4	5	3	3	4	4	4
4	5	4	3	4	3	3	3	4	4
2	2	1	3	2	2	1	3	1	2
3	4	3	4	4	4	4	3	4	3
3	4	3	5	3	4	4	5	4	4
3	5	3	4	4	4	3	4	4	3
3	5	3	5	5	4	3	5	4	5
4	5	4	4	5	4	5	4	5	5
5	3	3	4	4	5	5	4	4	4
3	5	4	5	4	4	4	3	4	4
1	1	4	3	4	4	4	3	3	3
3	1	1	2	2	1	2	2	3	2
3	1	2	2	3	1	1	2	2	2
5	5	5	4	3	4	5	3	4	4
4	4	3	5	4	3	4	3	3	3
4	5	4	5	4	5	5	4	3	4
4	5	4	3	5	4	4	4	3	3
4	4	4	4	4	3	4	3	5	3
4	4	4	3	4	4	4	5	3	4
4	5	5	4	5	5	5	4	4	3
3	4	5	5	4	3	3	5	4	3
5	4	4	4	4	3	5	4	4	5
1	2	3	4	4	3	4	4	4	5
3	4	4	4	3	4	3	4	4	4
4	3	5	4	4	5	3	5	4	5
3	5	3	4	5	4	4	5	3	3
4	3	5	3	4	3	3	3	4	4
1	2	2	3	3	3	2	3	2	2
4	3	4	4	4	4	4	3	4	4
5	4	4	3	4	3	3	5	4	4
4	3	4	5	4	5	3	5	4	3
4	3	4	3	5	5	4	4	4	5
4	4	5	4	4	4	4	5	5	3
4	3	3	5	4	3	4	4	4	4
2	2	5	4	1	2	1	1	2	2
4	4	3	5	5	4	4	4	3	4

4	4	4	5	3	4	4	5	4	3
4	4	4	3	5	3	3	3	3	3
3	4	3	4	4	5	4	4	3	5
2	1	5	5	4	3	4	5	4	3
2	2	1	2	3	3	3	2	2	1
3	3	1	3	3	1	1	2	2	2
3	4	2	2	3	3	5	4	4	4
2	3	1	2	3	3	1	1	1	2
4	4	4	5	4	5	5	5	5	5
4	5	3	4	3	4	5	5	5	3
5	5	3	5	5	4	4	4	5	3
5	4	4	3	4	4	4	4	5	5
1	3	2	2	2	2	1	3	3	1
4	4	3	5	4	4	3	4	5	4
3	4	4	3	5	5	4	3	3	3
4	3	4	4	5	4	3	4	5	5
4	4	5	5	4	3	4	3	5	4
4	4	3	5	4	4	5	4	4	4
3	4	3	5	4	4	3	4	4	4
5	5	3	3	4	4	5	3	4	4
4	4	5	4	4	3	5	3	4	5
4	3	4	4	2	1	1	1	2	1
4	3	4	5	3	5	4	5	5	5
3	5	5	5	4	3	4	3	5	4
4	4	4	3	4	3	5	4	4	3
4	3	3	4	3	4	3	4	4	5
4	5	4	4	3	4	4	4	4	5
4	4	4	3	4	4	5	4	5	5
4	3	5	3	4	3	5	5	4	5
5	4	4	5	4	4	4	4	4	4
1	2	1	3	2	1	1	2	3	1
5	4	4	3	3	5	5	3	4	4
5	5	5	5	4	3	3	3	4	4
4	3	5	4	5	3	4	5	4	4
2	2	2	2	2	3	2	3	3	2
4	5	3	4	2	2	1	1	1	1
2	1	2	1	1	2	2	2	1	2
4	4	4	4	3	4	5	5	5	4
4	5	4	5	4	3	4	4	4	4
5	4	5	5	4	4	4	4	5	4
4	4	4	4	5	3	3	4	4	5
4	3	4	4	5	5	4	3	3	4
3	4	4	3	4	5	3	3	5	5
2	2	5	4	4	4	4	5	4	5
2	3	1	1	1	3	2	2	1	2
3	4	5	3	4	4	5	4	4	4
4	4	4	5	5	4	5	4	5	5

5	4	4	4	4	4	4	5	4	4
3	5	5	4	3	4	3	3	4	4
3	1	2	1	2	2	2	2	2	1
4	3	4	4	3	5	4	5	5	4
5	4	5	4	3	4	5	4	3	4
3	3	3	4	5	4	4	3	5	4
4	3	3	4	3	3	4	3	5	3
1	2	5	4	4	4	4	4	5	4
5	4	5	5	3	3	4	3	3	4
2	2	3	3	2	2	1	2	2	2
4	4	4	5	4	3	4	4	5	4
4	3	4	3	3	3	4	4	4	5
5	4	4	5	4	4	4	4	5	4
4	3	4	4	3	3	4	5	3	4
3	4	5	4	4	5	3	4	4	4
5	4	4	4	4	4	4	3	4	4
5	4	1	2	4	3	4	5	3	5
3	3	4	5	3	5	5	5	4	4
3	4	3	4	4	5	4	3	4	3
4	4	3	4	5	5	5	4	4	5
4	4	3	5	5	5	4	5	3	5
4	3	3	5	3	3	5	5	4	4
4	4	4	5	3	5	4	3	4	4
4	3	5	4	5	3	3	4	5	3
4	4	4	3	3	5	3	4	3	5
4	5	3	4	4	4	3	3	5	4
4	5	3	5	4	4	5	3	3	4
5	4	4	4	3	3	5	3	4	4
4	4	3	4	5	4	4	5	5	5
5	5	4	3	4	3	4	3	5	3
3	4	4	3	5	5	4	3	4	4
4	3	5	4	5	4	4	4	4	4
2	2	3	3	1	2	1	2	3	2
4	5	4	4	3	3	4	5	4	5
5	3	3	5	4	3	3	4	4	4
2	3	3	2	3	1	3	2	2	2
3	4	4	3	4	5	5	3	4	3
4	4	4	4	4	3	4	5	4	5
3	2	3	2	2	3	1	2	2	3
4	5	5	5	5	4	4	5	3	3
5	5	5	5	4	5	4	4	4	4
3	3	4	5	1	1	2	2	1	1
4	4	4	3	1	2	1	2	1	2
5	4	4	4	3	5	4	5	3	5
4	4	4	3	5	4	3	4	4	4
3	4	3	4	4	4	4	4	5	5
4	4	4	5	4	4	4	4	4	4

3	5	3	5	5	4	3	4	4	3
3	4	3	4	4	4	4	4	3	3
4	4	5	4	4	3	3	3	4	5
1	3	2	2	3	3	2	2	1	1
4	3	4	5	4	4	3	4	3	5
5	3	3	4	3	4	4	3	3	4
4	5	5	5	4	4	4	4	3	4
4	4	5	4	4	5	3	5	4	3
3	5	4	4	2	1	1	2	1	2
3	5	4	4	4	5	4	4	5	4
3	4	5	4	4	4	4	3	4	3
5	5	3	4	3	4	4	4	3	4
4	3	3	3	3	4	3	3	4	5
4	5	4	5	4	3	4	3	4	4
3	5	4	3	3	3	4	4	5	4
5	3	5	5	4	4	4	4	4	4
4	5	5	4	4	5	4	4	4	3
2	1	3	2	2	2	2	1	3	1
4	3	4	4	3	4	4	3	5	4
5	4	3	4	5	4	4	4	4	5
4	5	4	4	5	5	5	5	5	4
3	4	4	4	5	5	4	5	5	3
5	4	5	3	4	3	5	4	4	3
5	4	3	4	2	2	2	2	2	1
3	3	4	4	4	4	4	3	5	3
3	3	5	4	3	4	5	4	3	4
4	4	4	4	5	5	3	4	4	4
5	4	4	3	4	4	5	5	3	3
4	5	5	4	3	3	4	4	5	4
3	2	1	3	1	3	3	1	2	2
4	4	3	4	4	3	4	3	4	4
5	4	4	4	5	4	5	3	4	4
5	5	4	4	4	4	5	5	5	3
4	5	5	4	5	4	4	5	4	3
4	5	4	3	4	5	4	4	3	4
2	1	3	4	4	4	4	4	3	4
3	4	4	5	4	4	4	4	4	4
4	3	5	5	5	3	4	5	4	5
3	3	5	3	5	4	4	4	3	3
1	2	3	5	3	4	4	3	4	4
3	5	1	1	4	4	3	3	4	5
4	4	3	5	5	3	4	4	5	5
4	4	4	4	5	3	4	5	4	4
4	3	4	3	2	2	1	2	1	1
3	2	2	3	1	3	2	2	2	2
3	4	5	5	4	4	4	3	5	4
4	4	4	5	3	3	4	5	5	4

3	5	2	2	3	3	3	5	4	5
5	4	4	5	1	2	2	2	1	2
3	4	4	4	3	3	3	4	4	5
3	3	4	5	4	3	3	4	4	4
5	5	3	5	3	4	4	3	4	4
5	3	5	3	1	2	2	1	1	2
5	4	5	4	4	4	5	3	4	5
2	1	3	2	2	3	1	2	2	2
5	5	2	2	5	4	4	4	4	5
4	3	3	4	3	5	3	5	3	4
4	4	5	5	5	4	3	5	3	5
3	5	5	3	4	5	4	5	4	5
2	2	3	2	2	3	2	3	2	3
2	2	3	4	4	4	3	4	4	3
4	4	3	5	1	1	1	2	2	1
4	3	4	3	3	5	4	3	5	4
4	3	5	4	4	4	4	5	5	4
5	4	3	3	4	3	4	4	4	3
4	5	4	3	4	3	4	3	4	5
3	4	5	5	3	5	4	4	3	5
4	3	4	4	3	4	4	5	5	5
3	4	5	3	3	3	4	4	4	3
5	4	4	4	5	4	3	5	4	4
3	3	4	4	4	4	5	5	3	4
3	4	4	4	5	3	5	4	3	4
4	3	4	3	4	5	4	5	3	5
2	2	4	5	3	4	3	5	4	5
5	4	5	3	3	3	4	3	4	5
3	4	3	4	4	4	4	5	5	3
4	4	1	1	4	5	3	4	4	3
4	5	5	4	4	4	4	5	3	5
1	2	1	2	2	2	2	3	2	2
3	2	1	2	1	2	3	2	2	2
3	3	3	4	3	4	3	3	3	4
3	4	4	4	4	4	4	3	4	4
5	3	4	4	4	3	4	5	3	4
5	5	4	3	5	4	3	4	3	3
4	3	5	4	3	3	3	4	4	4
3	4	5	5	5	3	3	4	4	3
2	2	2	3	2	1	2	1	2	2
3	4	5	4	4	5	4	5	4	4
4	3	3	4	4	3	4	3	5	5
4	4	5	4	5	3	4	4	3	5
2	2	2	1	2	2	1	3	3	1
4	5	3	4	4	4	4	4	4	4
4	3	4	5	4	3	4	4	3	5
4	3	3	3	3	4	3	4	5	5

3	4	5	4	4	3	5	5	4	3
4	3	5	5	4	4	5	4	3	3
4	5	4	5	4	4	4	4	4	3
2	1	3	1	3	2	2	3	3	2
2	3	1	1	1	1	2	3	1	1
1	1	3	2	2	3	2	3	2	1
4	5	4	4	4	4	4	4	3	4
5	5	4	3	4	5	4	4	4	4
4	4	3	4	4	5	4	3	4	3
2	1	3	5	4	4	4	4	4	4
5	5	2	1	3	4	4	4	3	5
2	1	1	1	2	2	2	2	3	2
5	3	3	4	3	4	4	4	3	5
4	4	4	4	3	4	3	3	5	4
5	3	4	4	5	3	3	4	4	4
5	4	5	5	4	4	4	3	4	5
4	4	5	3	4	3	4	3	4	4
4	4	1	2	4	4	3	3	5	5
2	3	2	2	2	2	2	2	2	3
4	3	4	5	3	5	5	4	3	5
3	3	3	4	3	4	5	3	5	5
3	2	3	2	1	1	3	2	3	2
1	2	4	4	4	4	3	5	5	4
1	1	5	4	5	5	4	4	5	4
2	2	2	1	2	2	3	3	1	2
1	2	3	3	5	4	5	3	4	4
3	3	5	3	4	4	3	4	4	3
4	4	3	4	3	3	4	4	4	3
3	4	4	4	3	4	5	3	3	5
5	4	5	5	4	5	4	3	4	3
4	5	5	4	3	4	4	4	4	4
4	4	3	4	5	4	5	4	5	3
4	4	4	4	3	3	4	4	3	4
4	4	5	3	4	5	5	4	4	5
3	2	2	2	2	2	3	3	2	2
5	5	5	4	2	1	2	1	1	1
3	4	4	5	4	4	3	4	4	3
4	3	4	4	3	3	5	4	5	4
4	5	3	5	5	4	4	5	4	5
3	4	3	4	4	5	4	4	3	5
4	3	4	5	3	4	3	4	3	5
3	4	4	4	3	3	3	4	4	3
4	5	4	4	4	4	4	3	3	5
5	5	4	3	5	3	5	4	4	5
4	3	5	3	4	4	5	4	5	3
3	4	5	4	5	5	5	3	5	3
5	4	1	1	3	5	4	5	3	5

5	4	2	2	3	4	5	4	3	5
4	4	4	5	4	3	5	3	5	4
4	5	3	4	4	5	4	5	5	4
5	4	4	4	4	3	4	4	4	5
2	1	4	4	4	4	3	5	3	4
4	4	3	4	5	5	4	4	4	4
3	3	4	4	3	4	3	4	5	3
4	4	4	4	3	3	3	3	4	4
4	3	5	4	4	4	5	5	3	3
4	4	5	3	5	3	3	3	4	4
5	4	5	3	4	5	4	3	5	5
4	5	5	4	5	4	5	5	4	3
4	4	4	4	4	3	3	4	4	4
3	4	4	5	4	5	3	5	4	4
5	4	4	5	3	4	3	3	4	5
2	1	2	3	2	3	3	1	2	2
4	4	4	4	3	4	4	4	3	4
4	3	4	4	5	4	4	5	3	4
4	4	5	4	4	3	4	4	4	5
4	5	4	4	3	4	5	4	4	4
5	5	5	3	3	5	5	5	5	5
3	2	1	3	1	2	1	3	2	2
5	4	5	4	4	3	4	4	3	4
3	4	3	5	4	3	4	5	4	4
5	4	5	3	5	4	4	5	3	4
4	4	3	4	4	3	5	5	4	5
5	4	5	4	5	3	3	3	5	5
4	4	1	1	4	3	3	4	4	4
5	5	5	3	4	4	5	4	4	4
5	5	4	3	4	4	5	4	4	4
1	3	2	2	2	2	1	2	1	1
5	3	3	3	3	4	4	4	3	4
4	4	4	4	3	3	4	5	3	4
3	1	2	2	1	1	2	2	3	3
2	2	1	1	3	2	2	1	2	2
2	2	4	3	4	5	4	4	4	5
4	5	4	5	3	4	5	4	5	4
4	3	3	3	4	4	4	4	4	3
4	3	3	5	3	5	4	3	5	5
4	4	3	5	4	3	3	5	4	4
3	3	4	4	5	4	5	4	4	3
4	3	3	4	1	1	1	1	2	2
4	5	5	4	4	5	5	5	5	4
3	3	3	4	5	4	4	3	4	5
4	4	4	5	5	3	3	3	4	3
5	4	4	3	4	5	5	4	4	4
4	4	3	5	3	3	3	4	5	3

4	5	3	5	5	3	5	5	3	4
4	3	4	5	5	4	5	4	4	4
4	3	4	3	3	4	5	4	3	4
2	2	4	3	2	2	2	2	1	2
4	4	5	5	4	4	4	5	3	4
1	1	2	2	2	2	3	2	2	3
5	3	4	4	5	3	4	5	5	4
4	4	5	4	3	3	4	5	4	4
5	4	3	4	4	4	3	4	3	3
4	3	5	4	4	4	4	4	5	4
4	5	4	4	4	4	3	3	4	4
5	3	4	4	3	4	4	4	4	5
3	4	3	3	3	5	4	4	4	5
4	4	5	5	3	5	4	3	4	5
3	3	4	4	5	4	3	3	4	4
4	4	4	4	3	4	4	4	3	5
3	2	2	2	1	1	3	1	1	3
5	3	4	5	4	5	5	5	4	4
4	4	4	5	4	4	4	4	4	4
3	4	5	4	1	2	2	2	2	2
3	3	2	1	5	3	3	4	5	5
4	5	5	4	4	3	4	3	4	5
5	5	5	3	5	4	5	3	5	5
1	2	3	2	3	3	2	2	1	2
2	2	2	2	2	1	2	2	2	3
2	2	2	1	1	3	1	2	2	2
4	5	4	5	4	4	3	4	5	4
4	4	5	5	4	3	4	3	4	4
3	3	4	4	4	3	3	4	5	3
5	4	4	3	4	5	3	5	4	3
4	4	4	4	5	4	3	3	3	5
1	2	4	4	4	4	4	4	5	3
5	4	4	5	3	4	4	4	5	4
4	4	3	4	4	5	3	3	4	4
3	4	4	4	4	5	4	4	5	4
3	5	4	5	4	4	3	4	5	5
4	4	4	3	5	3	4	3	5	3
3	5	3	4	4	4	4	5	5	5
5	3	3	5	1	2	1	2	2	1
4	3	4	5	4	4	4	5	5	4
4	4	4	3	3	4	4	4	5	5
4	4	4	4	4	3	4	4	3	4
5	4	5	3	4	3	3	3	3	4
5	4	5	4	4	4	3	5	5	4
3	2	3	2	3	2	2	3	2	2
4	3	4	4	5	4	4	4	3	4
3	2	1	2	2	3	2	2	1	3

5	5	4	4	3	4	3	4	4	4
3	4	4	4	3	4	5	4	4	5
4	4	4	4	3	4	4	4	5	4
4	4	4	4	4	5	3	4	4	4
4	3	4	4	5	3	5	5	3	4
3	2	2	1	3	2	3	2	3	2
3	5	3	4	4	3	4	4	3	3
5	4	4	5	5	3	5	4	3	4
4	4	2	1	5	4	3	5	4	4
4	4	4	4	4	4	3	4	3	4
3	4	5	4	4	4	5	4	4	5
2	2	1	3	3	1	2	1	2	1
2	2	2	2	2	3	2	3	1	1
4	3	2	2	4	4	4	5	4	5
4	4	4	5	4	4	4	4	5	5
5	3	5	3	4	4	3	3	3	4
1	1	5	4	5	4	4	3	5	4
3	4	4	4	4	4	4	3	3	3
5	4	4	4	3	3	5	5	3	4
4	3	5	3	4	4	5	4	3	4
4	3	4	4	4	4	5	5	4	5
4	5	3	4	4	3	5	5	5	4
5	3	4	4	3	3	3	5	4	3
4	4	4	5	4	4	5	4	4	4
4	3	5	5	4	4	4	4	4	4
4	4	4	3	3	4	3	3	5	4
3	5	4	3	5	4	5	5	3	3
3	4	4	4	3	4	3	4	4	4
3	1	2	1	3	1	2	3	3	1
3	4	3	5	5	5	4	4	4	3
2	2	1	2	2	1	1	2	2	2
3	3	4	4	3	4	4	3	3	4
3	3	3	5	4	3	4	3	4	4
4	3	4	4	4	3	3	4	3	4
3	4	3	5	5	5	3	4	4	4
3	3	3	4	3	4	3	4	4	5
1	3	2	2	2	1	2	2	3	2
3	4	5	3	4	4	3	3	4	3
4	5	3	4	3	4	4	4	3	5
2	2	2	2	2	3	2	1	3	2
5	4	4	4	3	5	4	4	4	4
3	4	4	3	4	4	4	5	3	4
5	5	3	5	5	4	3	4	4	5
4	3	5	5	1	2	2	2	1	1
3	4	4	4	3	4	4	3	3	5
4	5	4	4	5	4	4	4	4	3
5	4	5	4	4	5	3	3	4	5

3	5	5	4	4	5	3	5	5	3
4	5	4	4	4	4	4	5	3	5
4	4	4	3	3	4	5	4	4	3
3	3	4	3	4	5	3	3	3	3
3	5	1	2	5	3	4	3	4	4
2	2	2	1	1	1	2	2	1	2
5	5	3	3	4	4	3	3	4	4
3	4	4	3	3	4	4	3	4	4
5	4	4	3	4	3	4	4	3	3
3	5	4	4	5	4	5	4	4	5
3	3	3	3	3	3	5	4	5	3
4	4	3	3	3	4	3	5	4	4
5	4	5	4	5	3	4	4	4	3
4	5	5	3	1	1	1	2	1	1
5	4	4	4	4	4	4	3	3	4
4	4	4	5	4	3	3	5	4	4
5	5	4	3	4	4	3	4	4	3
2	1	4	5	4	3	3	4	3	4
4	5	5	4	4	3	3	4	5	4
4	5	4	4	4	4	4	3	3	5
4	4	4	4	5	4	3	4	4	3
5	5	4	4	4	5	4	4	3	5
2	2	1	2	3	1	3	3	2	1
2	2	1	3	2	2	1	2	2	1
3	4	3	4	4	5	5	3	4	4
4	3	5	5	4	4	4	3	4	4
3	4	3	3	5	5	3	4	3	3
3	5	4	4	4	4	3	5	3	5
3	3	1	2	4	4	4	4	4	3
5	5	5	3	4	3	3	4	5	3
2	3	3	1	3	1	2	2	1	2
3	1	3	1	2	1	2	3	2	2
4	4	4	4	4	3	4	5	4	4
3	3	5	4	4	4	5	4	5	4
4	4	4	5	4	3	4	4	4	3
3	4	4	3	5	4	5	4	5	5
1	2	3	3	2	2	2	2	1	2
3	5	4	5	4	4	4	4	4	3
4	4	4	4	4	3	4	5	3	4
2	2	2	1	1	3	2	2	2	2
4	4	1	1	4	4	4	4	4	4
3	4	4	5	4	3	5	5	4	4
5	4	4	5	3	3	4	4	3	4
3	3	2	2	5	3	4	4	4	4
4	5	4	4	3	3	3	4	4	5
1	2	2	3	1	1	2	3	3	2
3	4	4	4	5	4	3	4	3	4

4	5	4	4	4	4	4	4	4	5
3	3	4	5	4	4	4	3	3	5
4	5	3	4	5	3	3	4	4	4
4	5	4	3	4	3	3	3	4	4
2	2	1	3	2	2	1	3	1	2
3	4	3	4	4	4	4	3	4	3
3	4	3	5	3	4	4	5	4	4
3	5	3	4	4	4	3	4	4	3
3	5	3	5	5	4	3	5	4	5
4	5	4	4	5	4	5	4	5	5
5	3	3	4	4	5	5	4	4	4
3	5	4	5	4	4	4	3	4	4
1	1	4	3	4	4	4	3	3	3
3	1	1	2	2	1	2	2	3	2
3	1	2	2	3	1	1	2	2	2
5	5	5	4	3	4	5	3	4	4
4	4	3	5	4	3	4	3	3	3
4	5	4	5	4	5	5	4	3	4
4	5	4	3	5	4	4	4	3	3
4	4	4	4	4	3	4	3	5	3
4	4	4	3	4	4	4	5	3	4
4	5	5	4	5	5	5	4	4	3
3	4	5	5	4	3	3	5	4	3
5	4	4	4	4	3	5	4	4	5
1	2	3	4	4	3	4	4	4	5
3	4	4	4	3	4	3	4	4	4
4	3	5	4	4	5	3	5	4	5
3	5	3	4	5	4	4	5	3	3
4	3	5	3	4	3	3	3	4	4
1	2	2	3	3	3	2	3	2	2
4	3	4	4	4	4	4	3	4	4
5	4	4	3	4	3	3	5	4	4
4	3	4	5	4	5	3	5	4	3
4	3	4	3	5	5	4	4	4	5
4	4	5	4	4	4	4	5	5	3
4	3	3	5	4	3	4	4	4	4
2	2	5	4	1	2	1	1	2	2
4	4	3	5	5	4	4	4	3	4
4	4	4	5	3	4	4	5	4	3
4	4	4	3	5	3	3	3	3	3
3	4	3	4	4	5	4	4	3	5
2	1	5	5	4	3	4	5	4	3
2	2	1	2	3	3	3	2	2	1
3	3	1	3	3	1	1	2	2	2
3	4	2	2	3	3	5	4	4	4
2	3	1	2	3	3	1	1	1	2
4	4	4	5	4	5	5	5	5	5

4	5	3	4	3	4	5	5	5	3
5	5	3	5	5	4	4	4	5	3
5	4	4	3	4	4	4	4	5	5
1	3	2	2	2	2	1	3	3	1
4	4	3	5	4	4	3	4	5	4
3	4	4	3	5	5	4	3	3	3
4	3	4	4	5	4	3	4	5	5
4	4	5	5	4	3	4	3	5	4
4	4	3	5	4	4	5	4	4	4
3	4	3	5	4	4	3	4	4	4
5	5	3	3	4	4	5	3	4	4
4	4	5	4	4	3	5	3	4	5
4	3	4	4	2	1	1	1	2	1
4	3	4	5	3	5	4	5	5	5
3	5	5	5	4	3	4	3	5	4
4	4	4	3	4	3	5	4	4	3
4	3	3	4	3	4	3	4	4	5
4	5	4	4	3	4	4	4	4	5
4	4	4	3	4	4	5	4	5	5
4	3	5	3	4	3	5	5	4	5
5	4	4	5	4	4	4	4	4	4
1	2	1	3	2	1	1	2	3	1
5	4	4	3	3	5	5	3	4	4
5	5	5	5	4	3	3	3	4	4
4	3	5	4	5	3	4	5	4	4
2	2	2	2	2	3	2	3	3	2
4	5	3	4	2	2	1	1	1	1
2	1	2	1	1	2	2	2	1	2
4	4	4	4	3	4	5	5	5	4
4	5	4	5	4	3	4	4	4	4
5	4	5	5	4	4	4	4	5	4
4	4	4	4	5	3	3	4	4	5
4	3	4	4	5	5	4	3	3	4
3	4	4	3	4	5	3	3	5	5
2	2	5	4	4	4	4	5	4	5
2	3	1	1	1	3	2	2	1	2
3	4	5	3	4	4	5	4	4	4
4	4	4	5	5	4	5	4	5	5
5	4	4	4	4	4	4	5	4	4
3	5	5	4	3	4	3	3	4	4
3	1	2	1	2	2	2	2	2	1
4	3	4	4	3	5	4	5	5	4
5	4	5	4	3	4	5	4	3	4
3	3	3	4	5	4	4	3	5	4
4	3	3	4	3	3	4	3	5	3
1	2	5	4	4	4	4	4	5	4
5	4	5	5	3	3	4	3	3	4

2	2	3	3	2	2	1	2	2	2
4	4	4	5	4	3	4	4	5	4
4	3	4	3	3	3	4	4	4	5
5	4	4	5	4	4	4	4	5	4
4	3	4	4	3	3	4	5	3	4
3	4	5	4	4	5	3	4	4	4
5	4	4	4	4	4	4	3	4	4
5	4	1	2	4	3	4	5	3	5
3	3	4	5	3	5	5	5	4	4
3	4	3	4	4	5	4	3	4	3
4	4	3	4	5	5	5	4	4	5
4	4	3	5	5	5	4	5	3	5
4	3	3	5	3	3	5	5	4	4
4	4	4	5	3	5	4	3	4	4
4	3	5	4	5	3	3	4	5	3
4	4	4	3	3	5	3	4	3	5
4	5	3	4	4	4	3	3	5	4
4	5	3	5	4	4	5	3	3	4
5	4	4	4	3	3	5	3	4	4
4	4	3	4	5	4	4	5	5	5
5	5	4	3	4	3	4	3	5	3
3	4	4	3	5	5	4	3	4	4
4	3	5	4	5	4	4	4	4	4
2	2	3	3	1	2	1	2	3	2
4	5	4	4	3	3	4	5	4	5
5	3	3	5	4	3	3	4	4	4
2	3	3	2	3	1	3	2	2	2
3	4	4	3	4	5	5	3	4	3
4	4	4	4	4	3	4	5	4	5
3	2	3	2	2	3	1	2	2	3
4	5	5	5	5	4	4	5	3	3
5	5	5	5	4	5	4	4	4	4
3	3	4	5	1	1	2	2	1	1
4	4	4	3	1	2	1	2	1	2
5	4	4	4	3	5	4	5	3	5
4	4	4	3	5	4	3	4	4	4
3	4	3	4	4	4	4	4	5	5
4	4	4	5	4	4	4	4	4	4
3	5	3	5	5	4	3	4	4	3
3	4	3	4	4	4	4	4	3	3
4	4	5	4	4	3	3	3	4	5
1	3	2	2	3	3	2	2	1	1
4	3	4	5	4	4	3	4	3	5
5	3	3	4	3	4	4	3	3	4
4	5	5	5	4	4	4	4	3	4
4	4	5	4	4	5	3	5	4	3
3	5	4	4	2	1	1	2	1	2

3	5	4	4	4	5	4	4	5	4
3	4	5	4	4	4	4	3	4	3
5	5	3	4	3	4	4	4	3	4
4	3	3	3	3	4	3	3	4	5
4	5	4	5	4	3	4	3	4	4
3	5	4	3	3	3	4	4	5	4
5	3	5	5	4	4	4	4	4	4
4	5	5	4	4	5	4	4	4	3
2	1	3	2	2	2	2	1	3	1
4	3	4	4	3	4	4	3	5	4
5	4	3	4	5	4	4	4	4	5
4	5	4	4	5	5	5	5	5	4
3	4	4	4	5	5	4	5	5	3
5	4	5	3	4	3	5	4	4	3
5	4	3	4	2	2	2	2	2	1
3	3	4	4	4	4	4	3	5	3
3	3	5	4	3	4	5	4	3	4
4	4	4	4	5	5	3	4	4	4
5	4	4	3	4	4	5	5	3	3
4	5	5	4	3	3	4	4	5	4
3	2	1	3	1	3	3	1	2	2
4	4	3	4	4	3	4	3	4	4
5	4	4	4	5	4	5	3	4	4
5	5	4	4	4	4	5	5	5	3
4	5	5	4	5	4	4	5	4	3
4	5	4	3	4	5	4	4	3	4
2	1	3	4	4	4	4	4	3	4
3	4	4	5	4	4	4	4	4	4
4	3	5	5	5	3	4	5	4	5
3	3	5	3	5	4	4	4	3	3
1	2	3	5	3	4	4	3	4	4
3	5	1	1	4	4	3	3	4	5
4	4	3	5	5	3	4	4	5	5
4	4	4	4	5	3	4	5	4	4
4	3	4	3	2	2	1	2	1	1
3	2	2	3	1	3	2	2	2	2
3	4	5	5	4	4	4	3	5	4
4	4	4	5	3	3	4	5	5	4
3	5	2	2	3	3	3	5	4	5
5	4	4	5	1	2	2	2	1	2
3	4	4	4	3	3	3	4	4	5
3	3	4	5	4	3	3	4	4	4
5	5	3	5	3	4	4	3	4	4
5	3	5	3	1	2	2	1	1	2
5	4	5	4	4	4	5	3	4	5
2	1	3	2	2	3	1	2	2	2
5	5	2	2	5	4	4	4	4	5

4	3	3	4	3	5	3	5	3	4
4	4	5	5	5	4	3	5	3	5
3	5	5	3	4	5	4	5	4	5
2	2	3	2	2	3	2	3	2	3
2	2	3	4	4	4	3	4	4	3
4	4	3	5	1	1	1	2	2	1
4	3	4	3	3	5	4	3	5	4
4	3	5	4	4	4	4	5	5	4
5	4	3	3	4	3	4	4	4	3
4	5	4	3	4	3	4	3	4	5
3	4	5	5	3	5	4	4	3	5
4	3	4	4	3	4	4	5	5	5
3	4	5	3	3	3	4	4	4	3
5	4	4	4	5	4	3	5	4	4
3	3	4	4	4	4	5	5	3	4
3	4	4	4	5	3	5	4	3	4
4	3	4	3	4	5	4	5	3	5
2	2	4	5	3	4	3	5	4	5
5	4	5	3	3	3	4	3	4	5
3	4	3	4	4	4	4	5	5	3
4	4	1	1	4	5	3	4	4	3
4	5	5	4	4	4	4	5	3	5
1	2	1	2	2	2	2	3	2	2
3	2	1	2	1	2	3	2	2	2
3	3	3	4	3	4	3	3	3	4
3	4	4	4	4	4	4	3	4	4
5	3	4	4	4	3	4	5	3	4
5	5	4	3	5	4	3	4	3	3
4	3	5	4	3	3	3	4	4	4
3	4	5	5	5	3	3	4	4	3
2	2	2	3	2	1	2	1	2	2
3	4	5	4	4	5	4	5	4	4
4	3	3	4	4	3	4	3	5	5
4	4	5	4	5	3	4	4	3	5
2	2	2	1	2	2	1	3	3	1
4	5	3	4	4	4	4	4	4	4
4	3	4	5	4	3	4	4	3	5
4	3	3	3	3	4	3	4	5	5
3	4	5	4	4	3	5	5	4	3
4	3	5	5	4	4	5	4	3	3
4	5	4	5	4	4	4	4	4	3
2	1	3	1	3	2	2	3	3	2
2	3	1	1	1	1	2	3	1	1
1	1	3	2	2	3	2	3	2	1
4	5	4	4	4	4	4	4	3	4
5	5	4	3	4	5	4	4	4	4
4	4	3	4	4	5	4	3	4	3

2	1	3	5	4	4	4	4	4	4
5	5	2	1	3	4	4	4	3	5
2	1	1	1	2	2	2	2	3	2
5	3	3	4	3	4	4	4	3	5
4	4	4	4	3	4	3	3	5	4
5	3	4	4	5	3	3	4	4	4
5	4	5	5	4	4	4	3	4	5
4	4	5	3	4	3	4	3	4	4
4	4	1	2	4	4	3	3	5	5
2	3	2	2	2	2	2	2	2	3
4	3	4	5	3	5	5	4	3	5
3	3	3	4	3	4	5	3	5	5
3	2	3	2	1	1	3	2	3	2
1	2	4	4	4	4	3	5	5	4
1	1	5	4	5	5	4	4	5	4
2	2	2	1	2	2	3	3	1	2
1	2	3	3	5	4	5	3	4	4
3	3	5	3	4	4	3	4	4	3
4	4	3	4	3	3	4	4	4	3
3	4	4	4	3	4	5	3	3	5
5	4	5	5	4	5	4	3	4	3
4	5	5	4	3	4	4	4	4	4
4	4	3	4	5	4	5	4	5	3
4	4	4	4	3	3	4	4	3	4
4	4	5	3	4	5	5	4	4	5
3	2	2	2	2	2	3	3	2	2
5	5	5	4	2	1	2	1	1	1
3	4	4	5	4	4	3	4	4	3
4	3	4	4	3	3	5	4	5	4
4	5	3	5	5	4	4	5	4	5
3	4	3	4	4	5	4	4	3	5
4	3	4	5	3	4	3	4	3	5
3	4	4	4	3	3	3	4	4	3
4	5	4	4	4	4	4	3	3	5
5	5	4	3	5	3	5	4	4	5
4	3	5	3	4	4	5	4	5	3
3	4	5	4	5	5	5	3	5	3
5	4	1	1	3	5	4	5	3	5
5	4	2	2	3	4	5	4	3	5
4	4	4	5	4	3	5	3	5	4
4	5	3	4	4	5	4	5	5	4
5	4	4	4	4	3	4	4	4	5
2	1	4	4	4	4	3	5	3	4
4	4	3	4	5	5	4	4	4	4
3	3	4	4	3	4	3	4	5	3
4	4	4	4	3	3	3	3	4	4
4	3	5	4	4	4	5	5	3	3

4	4	5	3	5	3	3	3	4	4
5	4	5	3	4	5	4	3	5	5
4	5	5	4	5	4	5	5	4	3
4	4	4	4	4	3	3	4	4	4
3	4	4	5	4	5	3	5	4	4
5	4	4	5	3	4	3	3	4	5
2	1	2	3	2	3	3	1	2	2
4	4	4	4	3	4	4	4	3	4
4	3	4	4	5	4	4	5	3	4
4	4	5	4	4	3	4	4	4	5
4	5	4	4	3	4	5	4	4	4
5	5	5	3	3	5	5	5	5	5
3	2	1	3	1	2	1	3	2	2
5	4	5	4	4	3	4	4	3	4
3	4	3	5	4	3	4	5	4	4
5	4	5	3	5	4	4	5	3	4
4	4	3	4	4	3	5	5	4	5
5	4	5	4	5	3	3	3	5	5
4	4	1	1	4	3	3	4	4	4
5	5	5	3	4	4	5	4	4	4
5	5	4	3	4	4	5	4	4	4
1	3	2	2	2	2	1	2	1	1
5	3	3	3	3	4	4	4	3	4
4	4	4	4	3	3	4	5	3	4
3	1	2	2	1	1	2	2	3	3
2	2	1	1	3	2	2	1	2	2
2	2	4	3	4	5	4	4	4	5
4	5	4	5	3	4	5	4	5	4
4	3	3	3	4	4	4	4	4	3
4	3	3	5	3	5	4	3	5	5
4	4	3	5	4	3	3	5	4	4
3	3	4	4	5	4	5	4	4	3
4	3	3	4	1	1	1	1	2	2
4	5	5	4	4	5	5	5	5	4
3	3	3	4	5	4	4	3	4	5
4	4	4	5	5	3	3	3	4	3
5	4	4	3	4	5	5	4	4	4
4	4	3	5	3	3	3	4	5	3
4	5	3	5	5	3	5	5	3	4
4	3	4	5	5	4	5	4	4	4
4	3	4	3	3	4	5	4	3	4
2	2	4	3	2	2	2	2	1	2
4	4	5	5	4	4	4	5	3	4
1	1	2	2	2	2	3	2	2	3
5	3	4	4	5	3	4	5	5	4
4	4	5	4	3	3	4	5	4	4
5	4	3	4	4	4	3	4	3	3

4	3	5	4	4	4	4	4	5	4
4	5	4	4	4	4	3	3	4	4
5	3	4	4	3	4	4	4	4	5
3	4	3	3	3	5	4	4	4	5
4	4	5	5	3	5	4	3	4	5
3	3	4	4	5	4	3	3	4	4
4	4	4	4	3	4	4	4	3	5
3	2	2	2	1	1	3	1	1	3
5	3	4	5	4	5	5	5	4	4
4	4	4	5	4	4	4	4	4	4
3	4	5	4	1	2	2	2	2	2
3	3	2	1	5	3	3	4	5	5
4	5	5	4	4	3	4	3	4	5
5	5	5	3	5	4	5	3	5	5
1	2	3	2	3	3	2	2	1	2
2	2	2	2	2	1	2	2	2	3
2	2	2	1	1	3	1	2	2	2
4	5	4	5	4	4	3	4	5	4
4	4	5	5	4	3	4	3	4	4
3	3	4	4	4	3	3	4	5	3
5	4	4	3	4	5	3	5	4	3
4	3	4	5	5	4	5	4	4	4
4	3	4	3	3	4	5	4	3	4
2	2	4	3	2	2	2	2	1	2
4	4	5	5	4	4	4	5	3	4
1	1	2	2	2	2	3	2	2	3
5	3	4	4	5	3	4	5	5	4
4	4	5	4	3	3	4	5	4	4
5	4	3	4	4	4	3	4	3	3
4	3	5	4	4	4	4	4	5	4
4	5	4	4	4	4	3	3	4	4
5	3	4	4	3	4	4	4	4	5
3	4	3	3	3	5	4	4	4	5
4	4	5	5	3	5	4	3	4	5
3	3	4	4	5	4	3	3	4	4
4	4	4	4	3	4	4	4	3	5
3	2	2	2	1	1	3	1	1	3
5	3	4	5	4	5	5	5	4	4
4	4	4	5	4	4	4	4	4	4
3	4	5	4	1	2	2	2	2	2
3	3	2	1	5	3	3	4	5	5
4	5	5	4	4	3	4	3	4	5
5	5	5	3	5	4	5	3	5	5
1	2	3	2	3	3	2	2	1	2
2	2	2	2	2	1	2	2	2	3
2	2	2	1	1	3	1	2	2	2
4	5	4	5	4	4	3	4	5	4

4	4	5	5	4	3	4	3	4	4
3	3	4	4	4	3	3	4	5	3
5	4	4	3	4	5	3	5	4	3
4	3	4	5	5	4	5	4	4	4
4	3	4	3	3	4	5	4	3	4
2	2	4	3	2	2	2	2	1	2
4	4	5	5	4	4	4	5	3	4
1	1	2	2	2	2	3	2	2	3
5	3	4	4	5	3	4	5	5	4
4	4	5	4	3	3	4	5	4	4
5	4	3	4	4	4	3	4	3	3
4	3	5	4	4	4	4	4	5	4
4	5	4	4	4	4	3	3	4	4
5	3	4	4	3	4	4	4	4	5
3	4	3	3	3	5	4	4	4	5
4	4	5	5	3	5	4	3	4	5
3	3	4	4	5	4	3	3	4	4
4	4	4	4	3	4	4	4	3	5
3	2	2	2	1	1	3	1	1	3
5	3	4	5	4	5	5	5	4	4
4	4	4	5	4	4	4	4	4	4
3	4	5	4	1	2	2	2	2	2
3	3	2	1	5	3	3	4	5	5
4	5	5	4	4	3	4	3	4	5
5	5	5	3	5	4	5	3	5	5
1	2	3	2	3	3	2	2	1	2
2	2	2	2	2	1	2	2	2	3
2	2	2	1	1	3	1	2	2	2
4	5	4	5	4	4	3	4	5	4
4	4	5	5	4	3	4	3	4	4
3	3	4	4	4	3	3	4	5	3
5	4	4	3	4	5	3	5	4	3
4	3	4	5	5	4	5	4	4	4
4	3	4	3	3	4	5	4	3	4
2	2	4	3	2	2	2	2	1	2
4	4	5	5	4	4	4	5	3	4
1	1	2	2	2	2	3	2	2	3
5	3	4	4	5	3	4	5	5	4
4	4	5	4	3	3	4	5	4	4
5	4	3	4	4	4	3	4	3	3
4	3	5	4	4	4	4	4	5	4
4	5	4	4	4	4	3	3	4	4
5	3	4	4	3	4	4	4	4	5
3	4	3	3	3	5	4	4	4	5
4	4	5	5	3	5	4	3	4	5
3	3	4	4	5	4	3	3	4	4
4	4	4	4	3	4	4	4	3	5

3	2	2	2	1	1	3	1	1	3
5	3	4	5	4	5	5	5	4	4
4	4	4	5	4	4	4	4	4	4
3	4	5	4	1	2	2	2	2	2
3	3	2	1	5	3	3	4	5	5
4	5	5	4	4	3	4	3	4	5
5	5	5	3	5	4	5	3	5	5
1	2	3	2	3	3	2	2	1	2
2	2	2	2	2	1	2	2	2	3
2	2	2	1	1	3	1	2	2	2
4	5	4	5	4	4	3	4	5	4
4	4	5	5	4	3	4	3	4	4
3	3	4	4	4	3	3	4	5	3
5	4	4	3	4	5	3	5	4	3
1	2	3	2	3	3	2	2	1	2
2	2	2	2	2	1	2	2	2	3
2	2	2	1	1	3	1	2	2	2
4	5	4	5	4	4	3	4	5	4
4	4	5	5	4	3	4	3	4	4
3	3	4	4	4	3	3	4	5	3
5	4	4	3	4	5	3	5	4	3
1	2	3	2	3	3	2	2	1	2
2	2	2	2	2	1	2	2	2	3
2	2	2	1	1	3	1	2	2	2
4	5	4	5	4	4	3	4	5	4
2	2	2	1	1	3	1	2	2	2

MAAS_7	MAAS_8	MAAS_9	MAAS_10	MAAS_11	MAAS_12	MAAS_13	MAAS_14	MAAS_15
4	4	4	3	4	4	4	4	4
4	4	4	3	5	4	5	5	4
4	4	5	3	5	3	4	4	4
3	5	4	3	4	4	4	4	4
5	4	5	5	3	4	4	3	4
4	4	5	4	4	5	4	5	4
3	4	3	5	3	3	5	4	5
5	5	5	4	4	4	5	4	5
1	4	3	4	4	4	5	3	1
4	3	4	5	4	4	4	5	4
4	4	4	4	4	3	3	4	3
4	4	4	5	3	4	4	3	5
4	4	4	4	4	4	3	5	3
3	3	3	3	3	5	4	5	3
2	3	2	3	2	3	2	2	5
4	4	4	5	4	4	4	3	3
3	2	1	2	3	2	3	1	1
5	4	5	4	4	4	4	5	5
3	5	5	4	4	3	3	3	3
4	4	4	4	3	4	5	4	3
4	3	4	4	3	4	4	4	4
5	3	4	3	4	5	5	4	4
1	2	2	3	2	1	3	1	2
4	1	2	2	1	1	2	1	4
4	4	4	4	4	4	4	3	4
5	3	4	4	3	4	3	5	3
4	3	3	5	3	5	5	4	4
4	4	4	3	4	4	3	4	4
3	1	2	2	3	1	3	2	2
2	2	2	3	1	3	1	1	2
5	4	3	4	4	3	4	5	3
4	3	3	5	3	5	4	5	4
3	5	5	3	4	4	4	4	5
4	4	3	4	5	4	4	4	3
5	3	4	5	5	5	3	4	3
4	4	4	4	5	4	3	5	4
3	3	3	4	3	5	3	5	4
4	5	3	5	5	4	4	4	4
4	4	5	4	5	4	3	3	4
5	3	3	4	3	4	4	4	3
3	3	3	4	4	5	4	3	3
5	5	3	4	5	4	4	4	3
4	3	4	3	4	5	4	4	4
3	1	1	2	2	1	2	2	2
4	4	5	4	5	4	4	4	5
2	1	2	2	2	3	1	1	3

4	3	5	4	5	4	4	4	5
1	2	3	3	3	2	2	3	4
4	5	4	3	4	5	5	4	5
3	5	3	4	4	3	3	4	4
3	4	5	4	3	4	5	3	3
4	4	3	4	3	3	5	3	5
4	5	5	4	4	5	4	3	4
3	2	2	3	2	3	3	3	4
3	5	5	5	4	5	4	4	4
4	4	3	4	4	4	3	4	4
3	3	2	2	2	2	3	2	2
4	5	3	4	4	5	3	4	5
3	3	5	5	3	4	5	5	3
3	4	4	4	3	5	5	4	3
1	3	5	4	4	4	4	4	4
3	4	5	5	5	4	4	4	3
4	4	3	3	4	4	5	3	3
5	5	5	3	4	5	4	3	5
4	5	4	5	4	3	4	4	4
4	4	3	4	4	4	4	4	4
4	4	4	4	3	4	5	5	4
4	4	4	5	5	4	4	4	4
4	5	4	5	4	3	4	4	4
2	3	3	1	3	1	2	3	2
3	3	3	4	4	4	3	4	4
3	5	3	5	4	3	4	4	4
4	5	4	4	5	4	3	4	3
4	3	5	4	3	5	3	3	4
4	4	4	5	3	3	4	3	5
4	5	5	5	5	3	4	3	5
4	4	4	3	5	3	3	5	5
2	3	4	3	3	3	4	4	5
4	3	3	3	3	3	4	5	4
5	4	3	4	5	4	5	3	4
5	3	3	4	5	4	3	5	4
4	4	3	4	4	5	4	4	4
5	3	3	4	4	3	5	3	3
3	3	5	3	3	5	3	4	4
3	3	5	5	4	5	4	4	3
5	4	4	3	4	3	4	3	5
2	3	2	1	1	2	1	2	1
2	2	2	2	2	2	3	1	2
4	4	3	5	3	4	4	4	4
5	3	4	3	3	5	3	3	5
5	3	4	3	5	4	3	3	3
5	4	5	3	4	5	4	5	3
4	4	4	5	5	3	4	4	4

5	5	5	5	4	4	5	4	4
2	1	1	2	1	1	2	1	1
2	2	3	2	3	1	3	3	2
4	3	4	3	3	4	5	4	4
4	5	5	4	4	4	4	4	5
4	3	3	4	4	4	4	3	3
5	4	3	5	3	4	4	3	4
3	1	3	1	1	2	2	1	1
4	5	4	5	5	3	5	3	3
4	3	4	4	5	4	3	3	3
2	2	2	1	1	2	2	1	1
3	4	5	4	4	3	4	3	5
4	3	4	3	4	5	4	5	4
3	3	5	3	5	4	5	4	4
5	4	4	3	5	3	4	4	4
4	5	3	5	4	3	4	4	4
3	2	2	1	3	1	1	2	1
3	4	5	5	4	4	3	3	5
3	5	4	3	3	4	4	4	4
3	5	5	4	5	5	5	4	4
4	4	5	5	4	4	4	4	3
5	4	4	4	4	3	3	4	3
2	2	2	2	3	1	3	2	3
5	3	4	5	5	3	5	3	4
4	5	3	4	5	5	5	5	5
5	4	4	5	4	4	4	4	4
5	4	3	4	4	3	4	3	5
4	4	4	5	3	4	4	4	5
5	3	4	4	4	4	4	4	4
4	4	4	3	4	4	4	4	4
3	4	3	5	4	3	5	4	4
2	3	1	3	2	2	3	2	4
2	3	1	2	2	1	2	3	4
5	5	5	5	5	4	4	4	3
4	4	4	5	4	5	3	5	5
4	4	4	5	3	4	4	4	4
5	3	4	4	4	4	4	5	3
3	3	4	5	5	4	3	4	4
5	4	4	4	4	4	4	4	5
5	4	4	5	3	4	5	4	3
4	4	4	4	5	3	5	4	4
4	4	4	3	4	4	3	4	4
4	3	4	3	4	4	3	3	3
5	3	4	3	5	5	3	5	3
4	4	4	4	4	5	4	4	4
3	4	3	5	5	4	5	4	4
4	4	4	5	5	5	4	5	4

1	1	2	2	1	2	2	1	5
5	3	3	4	5	3	4	3	4
4	3	5	4	5	5	5	5	4
3	4	3	5	5	3	5	5	4
4	2	1	2	2	1	1	2	5
3	5	5	4	3	4	5	4	4
4	2	2	1	2	2	1	1	4
2	4	3	3	3	5	5	4	1
5	3	5	5	4	4	3	3	4
3	4	4	4	5	4	5	5	3
5	4	5	4	4	3	4	4	3
5	3	4	3	4	4	3	4	4
3	5	4	3	5	5	4	4	4
3	1	2	1	1	2	1	2	2
3	2	3	1	3	2	2	1	3
5	5	3	3	4	4	4	3	5
2	3	3	1	1	1	2	2	4
4	5	4	4	4	4	4	3	4
4	4	4	5	4	4	4	3	4
5	3	4	5	3	4	4	5	4
5	5	3	5	5	3	4	3	4
2	2	2	2	1	3	3	2	3
3	5	4	4	3	3	4	4	3
5	4	5	4	3	5	5	4	3
4	2	2	1	2	2	2	2	4
3	4	4	5	3	5	4	5	5
4	5	3	3	5	5	4	4	4
4	1	2	1	1	2	2	2	3
4	4	3	3	4	4	5	4	4
5	5	5	3	4	5	3	3	3
2	4	4	5	4	4	3	4	1
4	3	5	3	4	3	4	4	4
3	5	4	5	3	4	5	4	4
5	5	4	4	4	3	4	4	5
4	3	3	5	4	4	4	3	3
5	3	3	3	4	5	3	4	3
4	5	3	4	5	5	4	5	4
4	4	4	5	3	4	5	4	4
4	5	5	5	4	5	3	5	3
2	3	1	2	1	2	2	1	2
4	4	5	3	3	4	5	4	5
4	3	5	4	4	4	4	5	4
4	5	3	3	4	3	4	4	3
2	2	2	1	1	3	2	2	4
1	4	4	5	4	5	3	4	2
3	2	1	3	1	1	1	1	2
4	4	5	3	4	3	4	5	4

4	4	4	4	3	5	4	3	3
4	5	5	3	4	4	5	4	4
3	4	3	5	4	5	4	4	5
5	3	4	5	4	5	3	4	3
5	2	1	1	2	2	1	2	4
4	5	5	3	3	3	4	5	4
1	3	3	1	2	2	2	3	4
4	4	4	5	3	4	4	5	4
3	3	5	4	4	4	4	4	5
4	5	4	5	5	4	4	4	4
4	4	3	4	4	4	4	3	5
3	1	1	2	2	3	3	3	2
4	1	1	2	1	1	1	2	2
4	4	3	3	3	5	4	5	3
3	3	4	5	4	4	3	4	5
4	5	4	5	4	4	5	5	5
4	4	4	4	4	4	5	3	3
3	5	5	5	4	4	5	3	4
2	3	3	2	1	2	3	3	4
3	4	4	4	5	3	4	5	1
4	5	3	3	4	5	3	4	3
4	3	5	3	4	4	4	3	5
5	4	3	4	5	3	4	4	5
3	5	4	4	5	3	3	5	4
4	3	4	4	4	5	4	3	4
5	4	3	5	4	3	4	4	3
4	3	4	4	3	4	3	3	3
3	4	4	3	4	5	5	5	4
4	4	3	3	3	5	4	4	4
4	4	4	5	5	3	4	4	4
5	4	4	4	5	5	4	4	5
4	4	4	4	4	4	4	3	3
4	4	4	4	4	5	4	5	5
3	3	4	4	3	3	5	4	4
5	1	1	1	2	2	2	2	4
4	3	4	4	4	4	3	4	4
3	3	5	5	4	4	3	5	4
4	3	4	4	4	3	3	5	4
3	5	3	3	5	4	5	3	4
4	4	3	5	5	4	3	4	5
5	5	4	4	3	3	3	3	3
1	1	1	2	2	2	2	2	1
3	4	4	4	3	5	3	4	3
4	1	2	1	1	1	1	1	3
1	3	3	3	2	3	2	3	4
4	5	3	4	3	4	4	4	5
3	5	5	4	4	4	5	5	2

1	1	2	1	3	1	3	1	4
4	4	5	4	3	5	3	4	4
5	5	4	3	4	4	4	4	5
1	4	3	5	4	4	3	4	1
1	5	4	5	4	4	4	3	2
5	4	5	4	3	3	4	4	4
4	5	5	5	4	3	4	3	5
4	5	3	4	4	3	3	4	1
5	4	4	4	3	4	5	4	4
3	5	3	4	5	3	3	4	3
4	3	4	3	5	3	4	4	3
3	4	4	4	3	5	4	4	4
3	2	2	2	1	3	2	1	3
3	5	5	5	4	3	5	4	4
3	3	5	4	5	4	4	4	4
4	4	5	4	4	3	4	4	4
4	3	4	4	4	3	3	5	4
2	5	5	4	4	5	5	4	2
3	5	5	5	3	3	4	4	4
3	4	5	4	5	4	5	4	4
3	4	4	4	4	4	3	4	3
5	5	4	5	4	5	4	4	3
4	4	3	5	4	4	4	4	5
4	5	3	4	5	3	3	3	4
4	5	4	4	5	5	3	3	3
3	1	2	2	2	1	1	2	4
3	2	2	2	3	2	3	3	2
4	5	3	3	5	5	3	4	4
4	4	4	4	3	5	4	5	3
4	4	3	3	5	5	5	3	5
4	4	4	3	4	4	5	3	5
3	3	4	4	4	5	3	5	5
1	4	4	5	3	4	4	3	1
4	5	4	3	5	4	3	3	5
3	3	4	4	5	4	4	4	4
3	4	4	4	5	4	4	3	3
5	5	4	3	3	5	5	3	4
4	3	5	5	5	5	4	4	4
1	1	1	2	3	1	2	1	3
4	4	4	4	4	3	4	3	2
5	4	5	3	4	4	4	4	4
4	5	5	5	5	5	5	5	5
5	1	2	1	2	2	1	1	4
4	5	4	4	4	4	4	3	3
4	4	4	3	4	5	4	5	5
4	4	5	4	4	4	3	4	4
3	1	1	2	2	1	1	1	4

5	4	5	4	5	5	4	4	4
3	4	4	4	3	4	3	3	5
5	5	3	4	5	4	4	5	5
4	3	3	4	4	4	5	4	1
5	4	4	4	3	3	3	4	4
2	5	4	4	4	4	4	5	1
2	1	2	2	3	2	1	3	4
4	3	3	5	3	4	4	4	4
5	4	5	5	4	3	3	4	4
3	3	3	4	4	5	3	3	4
1	4	3	3	4	5	4	3	4
3	3	5	5	4	4	4	4	5
5	4	5	4	4	4	3	4	4
4	5	4	4	5	5	5	4	4
1	3	3	4	5	4	4	4	2
3	3	5	4	3	3	4	3	4
2	3	3	1	2	1	3	2	4
5	3	4	3	4	4	4	4	4
4	5	3	4	3	4	4	5	5
4	4	5	5	5	4	3	4	3
4	5	4	4	3	5	4	4	3
2	2	2	3	3	1	2	1	4
4	5	4	3	5	5	4	4	3
1	3	5	3	5	4	3	4	3
3	3	5	4	5	4	5	3	4
4	4	5	4	4	3	4	5	4
4	3	4	4	3	5	3	3	4
3	3	5	4	4	5	4	4	5
4	3	3	4	4	3	4	4	5
4	5	5	3	3	3	3	5	4
4	3	4	3	4	3	3	4	5
4	3	3	5	4	3	5	3	3
5	5	3	4	4	5	5	3	5
4	5	5	3	4	3	5	4	5
3	4	4	5	4	5	4	3	4
4	4	3	4	4	4	4	4	5
3	1	1	1	2	2	2	1	4
5	3	4	3	5	3	4	3	5
4	5	5	4	3	4	3	5	3
4	4	4	3	3	3	3	5	3
1	2	2	2	3	1	2	2	4
2	1	3	2	3	2	2	2	4
3	4	4	5	5	4	3	4	3
4	3	4	4	3	5	3	4	3
4	1	1	1	1	1	1	1	4
4	4	5	5	4	3	4	3	5
3	4	4	3	5	3	3	3	4

5	5	4	5	5	3	3	4	3
2	2	3	1	2	3	2	1	5
5	5	4	3	4	4	4	4	4
4	3	4	4	3	3	5	5	5
3	3	3	4	5	4	3	4	3
1	2	2	2	1	2	1	2	3
5	3	4	5	4	5	5	4	2
5	4	4	5	4	4	4	4	2
4	3	5	5	5	4	4	5	3
5	4	3	4	5	3	3	4	5
5	4	5	4	4	5	3	3	4
3	5	3	4	4	4	3	5	4
1	2	2	2	2	2	3	2	2
2	1	2	2	3	2	2	1	1
2	2	2	2	2	3	2	3	2
4	4	4	4	3	4	4	3	5
4	5	4	4	3	5	4	4	4
3	3	4	4	4	3	5	3	2
5	4	4	4	3	4	3	5	4
4	5	4	4	4	4	4	4	4
3	3	2	3	1	2	2	2	4
4	4	4	5	4	4	4	4	3
4	5	4	4	3	4	4	4	4
4	4	4	3	4	5	4	3	4
4	4	3	4	4	3	4	3	5
4	3	4	4	4	4	3	3	4
3	5	4	4	4	3	4	4	3
2	3	3	2	2	2	2	2	2
4	3	4	3	4	4	3	4	4
4	4	4	3	5	3	4	3	3
3	3	1	2	3	3	3	2	3
4	3	3	3	4	4	4	3	4
3	4	4	4	4	5	4	5	4
2	2	2	3	2	2	2	2	3
3	3	4	5	4	4	4	3	5
3	5	3	5	4	3	4	3	4
4	4	5	4	4	5	5	3	3
4	4	5	5	4	3	5	4	3
3	4	4	4	3	5	5	5	4
3	4	3	4	3	3	5	4	5
3	3	5	5	4	4	4	4	4
4	3	5	5	3	3	4	4	5
4	4	4	4	3	4	4	3	3
3	3	2	1	3	1	2	2	3
1	5	4	4	3	4	4	4	5
4	4	4	3	5	4	4	4	4
3	4	3	4	4	3	4	3	4

3	4	4	5	4	4	3	5	3
4	5	4	5	4	4	5	4	3
5	5	4	5	3	4	5	4	5
5	4	4	4	4	5	3	4	3
3	1	2	2	2	1	1	1	4
3	5	5	5	5	4	4	3	4
5	5	4	4	4	4	3	4	4
5	4	3	5	3	5	4	4	4
4	3	4	4	4	5	4	3	5
4	3	3	3	5	5	3	3	5
3	2	1	2	2	2	2	1	4
3	4	5	4	4	4	3	4	5
3	4	4	4	4	5	4	3	5
3	3	5	4	5	4	3	5	4
5	3	5	3	5	5	3	4	4
5	4	5	3	4	4	4	4	2
3	3	4	3	4	4	4	4	5
3	4	4	4	4	4	3	4	5
3	5	4	3	3	4	3	5	3
4	3	4	3	5	4	4	4	3
4	5	5	5	3	3	5	4	4
4	5	4	4	4	4	4	5	4
4	4	4	5	4	5	5	3	3
5	5	4	4	4	4	5	4	3
2	1	1	1	3	1	2	1	3
4	5	4	4	4	4	3	3	4
5	5	3	3	3	5	3	5	5
4	3	3	3	4	3	3	4	5
4	4	5	5	3	4	4	4	4
4	4	4	3	5	4	5	5	5
1	1	3	1	1	1	2	1	3
3	4	4	3	5	4	4	4	5
4	5	5	5	5	4	5	4	5
4	4	5	5	5	4	4	5	3
4	5	3	5	5	3	3	3	4
4	3	3	4	5	4	4	4	5
4	4	4	4	4	5	5	4	4
4	4	4	3	5	4	3	4	4
4	5	4	4	4	4	4	5	5
3	3	2	1	2	2	1	2	4
3	3	4	4	4	5	4	4	1
4	3	4	5	4	4	4	4	4
2	1	2	2	3	3	2	3	2
2	2	3	3	2	2	2	2	1
4	5	5	4	4	4	5	4	4
4	5	4	5	4	5	5	4	4
4	4	5	3	5	4	5	4	4

5	4	4	4	4	5	5	5	3
4	4	3	4	5	5	4	3	4
4	4	4	3	3	5	4	5	5
1	4	5	3	5	5	4	3	3
3	5	4	5	4	3	4	4	4
4	5	3	5	4	4	3	5	3
4	3	4	4	3	3	3	4	5
3	4	5	4	4	5	4	5	5
3	5	5	4	3	3	5	4	5
5	4	5	5	5	4	5	3	5
5	5	3	4	5	4	4	5	4
4	4	3	4	3	3	4	4	4
2	4	4	4	5	4	4	3	1
5	3	4	4	4	3	4	4	4
3	3	2	3	2	3	3	1	4
5	4	4	5	4	4	3	4	4
5	3	4	4	4	3	4	5	4
5	3	3	5	4	3	3	4	4
3	3	4	3	3	3	4	4	4
4	3	5	5	3	4	5	3	3
3	4	3	4	3	3	3	3	4
4	4	5	5	4	4	5	4	4
5	4	3	4	4	5	4	3	3
5	4	4	3	5	4	4	4	5
4	4	4	4	4	4	3	5	4
3	3	2	1	2	2	2	3	1
5	5	4	4	5	4	3	4	4
4	4	4	5	3	5	4	4	5
2	4	3	5	5	5	5	4	1
4	3	4	4	5	3	4	4	4
5	2	2	2	1	1	2	2	4
3	5	3	4	3	4	4	5	3
3	3	3	2	2	2	2	1	2
2	2	1	2	2	1	1	1	3
2	2	1	1	2	3	1	2	3
4	4	4	3	3	5	4	4	5
4	3	3	4	4	3	3	4	5
4	4	4	3	3	4	5	4	5
5	4	4	4	5	4	4	5	4
4	4	4	3	4	4	4	4	4
4	4	4	3	5	4	5	5	4
4	4	5	3	5	3	4	4	4
3	5	4	3	4	4	4	4	4
5	4	5	5	3	4	4	3	4
4	4	5	4	4	5	4	5	4
3	4	3	5	3	3	5	4	5
5	5	5	4	4	4	5	4	5

1	4	3	4	4	4	5	3	1
4	3	4	5	4	4	4	5	4
4	4	4	4	4	3	3	4	3
4	4	4	5	3	4	4	3	5
4	4	4	4	4	4	3	5	3
3	3	3	3	3	5	4	5	3
2	3	2	3	2	3	2	2	5
4	4	4	5	4	4	4	3	3
3	2	1	2	3	2	3	1	1
5	4	5	4	4	4	4	5	5
3	5	5	4	4	3	3	3	3
4	4	4	4	3	4	5	4	3
4	3	4	4	3	4	4	4	4
5	3	4	3	4	5	5	4	4
1	2	2	3	2	1	3	1	2
4	1	2	2	1	1	2	1	4
4	4	4	4	4	4	4	3	4
5	3	4	4	3	4	3	5	3
4	3	3	5	3	5	5	4	4
4	4	4	3	4	4	3	4	4
3	1	2	2	3	1	3	2	2
2	2	2	3	1	3	1	1	2
5	4	3	4	4	3	4	5	3
4	3	3	5	3	5	4	5	4
3	5	5	3	4	4	4	4	5
4	4	3	4	5	4	4	4	3
5	3	4	5	5	5	3	4	3
4	4	4	4	5	4	3	5	4
3	3	3	4	3	5	3	5	4
4	5	3	5	5	4	4	4	4
4	4	5	4	5	4	3	3	4
5	3	3	4	3	4	4	4	3
3	3	3	4	4	5	4	3	3
5	5	3	4	5	4	4	4	3
4	3	4	3	4	5	4	4	4
3	1	1	2	2	1	2	2	2
4	4	5	4	5	4	4	4	5
2	1	2	2	2	3	1	1	3
4	3	5	4	5	4	4	4	5
1	2	3	3	3	2	2	3	4
4	5	4	3	4	5	5	4	5
3	5	3	4	4	3	3	4	4
3	4	5	4	3	4	5	3	3
4	4	3	4	3	3	5	3	5
4	5	5	4	4	5	4	3	4
3	2	2	3	2	3	3	3	4
3	5	5	5	4	5	4	4	4

4	4	3	4	4	4	3	4	4
3	3	2	2	2	2	3	2	2
4	5	3	4	4	5	3	4	5
3	3	5	5	3	4	5	5	3
3	4	4	4	3	5	5	4	3
1	3	5	4	4	4	4	4	4
3	4	5	5	5	4	4	4	3
4	4	3	3	4	4	5	3	3
5	5	5	3	4	5	4	3	5
4	5	4	5	4	3	4	4	4
4	4	3	4	4	4	4	4	4
4	4	4	4	3	4	5	5	4
4	4	4	5	5	4	4	4	4
4	5	4	5	4	3	4	4	4
2	3	3	1	3	1	2	3	2
3	3	3	4	4	4	3	4	4
3	5	3	5	4	3	4	4	4
4	5	4	4	5	4	3	4	3
4	3	5	4	3	5	3	3	4
4	4	4	5	3	3	4	3	5
4	5	5	5	5	3	4	3	5
4	4	4	3	5	3	3	5	5
2	3	4	3	3	3	4	4	5
4	3	3	3	3	3	4	5	4
5	4	3	4	5	4	5	3	4
5	3	3	4	5	4	3	5	4
4	4	3	4	4	5	4	4	4
5	3	3	4	4	3	5	3	3
3	3	5	3	3	5	3	4	4
3	3	5	5	4	5	4	4	3
5	4	4	3	4	3	4	3	5
2	3	2	1	1	2	1	2	1
2	2	2	2	2	2	3	1	2
4	4	3	5	3	4	4	4	4
5	3	4	3	3	5	3	3	5
5	3	4	3	5	4	3	3	3
5	4	5	3	4	5	4	5	3
4	4	4	5	5	3	4	4	4
5	5	5	5	4	4	5	4	4
2	1	1	2	1	1	2	1	1
2	2	3	2	3	1	3	3	2
4	3	4	3	3	4	5	4	4
4	5	5	4	4	4	4	4	5
4	3	3	4	4	4	4	3	3
5	4	3	5	3	4	4	3	4
3	1	3	1	1	2	2	1	1
4	5	4	5	5	3	5	3	3

4	3	4	4	5	4	3	3	3
2	2	2	1	1	2	2	1	1
3	4	5	4	4	3	4	3	5
4	3	4	3	4	5	4	5	4
3	3	5	3	5	4	5	4	4
5	4	4	3	5	3	4	4	4
4	5	3	5	4	3	4	4	4
3	2	2	1	3	1	1	2	1
3	4	5	5	4	4	3	3	5
3	5	4	3	3	4	4	4	4
3	5	5	4	5	5	5	4	4
4	4	5	5	4	4	4	4	3
5	4	4	4	4	3	3	4	3
2	2	2	2	3	1	3	2	3
5	3	4	5	5	3	5	3	4
4	5	3	4	5	5	5	5	5
5	4	4	5	4	4	4	4	4
5	4	3	4	4	3	4	3	5
4	4	4	5	3	4	4	4	5
5	3	4	4	4	4	4	4	4
4	4	4	3	4	4	4	4	4
3	4	3	5	4	3	5	4	4
2	3	1	3	2	2	3	2	4
2	3	1	2	2	1	2	3	4
5	5	5	5	5	4	4	4	3
4	4	4	5	4	5	3	5	5
4	4	4	5	3	4	4	4	4
5	3	4	4	4	4	4	5	3
3	3	4	5	5	4	3	4	4
5	4	4	4	4	4	4	4	5
5	4	4	5	3	4	5	4	3
4	4	4	4	5	3	5	4	4
4	4	4	3	4	4	3	4	4
4	3	4	3	4	4	3	3	3
5	3	4	3	5	5	3	5	3
4	4	4	4	4	5	4	4	4
3	4	3	5	5	4	5	4	4
4	4	4	5	5	5	4	5	4
1	1	2	2	1	2	2	1	5
5	3	3	4	5	3	4	3	4
4	3	5	4	5	5	5	5	4
3	4	3	5	5	3	5	5	4
4	2	1	2	2	1	1	2	5
3	5	5	4	3	4	5	4	4
4	2	2	1	2	2	1	1	4
2	4	3	3	3	5	5	4	1
5	3	5	5	4	4	3	3	4

3	4	4	4	5	4	5	5	3
5	4	5	4	4	3	4	4	3
5	3	4	3	4	4	3	4	4
3	5	4	3	5	5	4	4	4
3	1	2	1	1	2	1	2	2
3	2	3	1	3	2	2	1	3
5	5	3	3	4	4	4	3	5
2	3	3	1	1	1	2	2	4
4	5	4	4	4	4	4	3	4
4	4	4	5	4	4	4	3	4
5	3	4	5	3	4	4	5	4
5	5	3	5	5	3	4	3	4
2	2	2	2	1	3	3	2	3
3	5	4	4	3	3	4	4	3
5	4	5	4	3	5	5	4	3
4	2	2	1	2	2	2	2	4
3	4	4	5	3	5	4	5	5
4	5	3	3	5	5	4	4	4
4	1	2	1	1	2	2	2	3
4	4	3	3	4	4	5	4	4
5	5	5	3	4	5	3	3	3
2	4	4	5	4	4	3	4	1
4	3	5	3	4	3	4	4	4
3	5	4	5	3	4	5	4	4
5	5	4	4	4	3	4	4	5
4	3	3	5	4	4	4	3	3
5	3	3	3	4	5	3	4	3
4	5	3	4	5	5	4	5	4
4	4	4	5	3	4	5	4	4
4	5	5	5	4	5	3	5	3
2	3	1	2	1	2	2	1	2
4	4	5	3	3	4	5	4	5
4	3	5	4	4	4	4	5	4
4	5	3	3	4	3	4	4	3
2	2	2	1	1	3	2	2	4
1	4	4	5	4	5	3	4	2
3	2	1	3	1	1	1	1	2
4	4	5	3	4	3	4	5	4
4	4	4	4	3	5	4	3	3
4	5	5	3	4	4	5	4	4
3	4	3	5	4	5	4	4	5
5	3	4	5	4	5	3	4	3
5	2	1	1	2	2	1	2	4
4	5	5	3	3	3	4	5	4
1	3	3	1	2	2	2	3	4
4	4	4	5	3	4	4	5	4
3	3	5	4	4	4	4	4	5

4	5	4	5	5	4	4	4	4
4	4	3	4	4	4	4	3	5
3	1	1	2	2	3	3	3	2
4	1	1	2	1	1	1	2	2
4	4	3	3	3	5	4	5	3
3	3	4	5	4	4	3	4	5
4	5	4	5	4	4	5	5	5
4	4	4	4	4	4	5	3	3
3	5	5	5	4	4	5	3	4
2	3	3	2	1	2	3	3	4
3	4	4	4	5	3	4	5	1
4	5	3	3	4	5	3	4	3
4	3	5	3	4	4	4	3	5
5	4	3	4	5	3	4	4	5
3	5	4	4	5	3	3	5	4
4	3	4	4	4	5	4	3	4
5	4	3	5	4	3	4	4	3
4	3	4	4	3	4	3	3	3
3	4	4	3	4	5	5	5	4
4	4	3	3	3	5	4	4	4
4	4	4	5	5	3	4	4	4
5	4	4	4	5	5	4	4	5
4	4	4	4	4	4	4	3	3
4	4	4	4	4	5	4	5	5
3	3	4	4	3	3	5	4	4
5	1	1	1	2	2	2	2	4
4	3	4	4	4	4	3	4	4
3	3	5	5	4	4	3	5	4
4	3	4	4	4	3	3	5	4
3	5	3	3	5	4	5	3	4
4	4	3	5	5	4	3	4	5
5	5	4	4	3	3	3	3	3
1	1	1	2	2	2	2	2	1
3	4	4	4	3	5	3	4	3
4	1	2	1	1	1	1	1	3
1	3	3	3	2	3	2	3	4
4	5	3	4	3	4	4	4	5
3	5	5	4	4	4	5	5	2
1	1	2	1	3	1	3	1	4
4	4	5	4	3	5	3	4	4
5	5	4	3	4	4	4	4	5
1	4	3	5	4	4	3	4	1
1	5	4	5	4	4	4	3	2
5	4	5	4	3	3	4	4	4
4	5	5	5	4	3	4	3	5
4	5	3	4	4	3	3	4	1
5	4	4	4	3	4	5	4	4

3	5	3	4	5	3	3	4	3
4	3	4	3	5	3	4	4	3
3	4	4	4	3	5	4	4	4
3	2	2	2	1	3	2	1	3
3	5	5	5	4	3	5	4	4
3	3	5	4	5	4	4	4	4
4	4	5	4	4	3	4	4	4
4	3	4	4	4	3	3	5	4
2	5	5	4	4	5	5	4	2
3	5	5	5	3	3	4	4	4
3	4	5	4	5	4	5	4	4
3	4	4	4	4	4	3	4	3
5	5	4	5	4	5	4	4	3
4	4	3	5	4	4	4	4	5
4	5	3	4	5	3	3	3	4
4	5	4	4	5	5	3	3	3
3	1	2	2	2	1	1	2	4
3	2	2	2	3	2	3	3	2
4	5	3	3	5	5	3	4	4
4	4	4	4	3	5	4	5	3
4	4	3	3	5	5	5	3	5
4	4	4	3	4	4	5	3	5
3	3	4	4	4	5	3	5	5
1	4	4	5	3	4	4	3	1
4	5	4	3	5	4	3	3	5
3	3	4	4	5	4	4	4	4
3	4	4	4	5	4	4	3	3
5	5	4	3	3	5	5	3	4
4	3	5	5	5	5	4	4	4
1	1	1	2	3	1	2	1	3
4	4	4	4	4	3	4	3	2
5	4	5	3	4	4	4	4	4
4	5	5	5	5	5	5	5	5
5	1	2	1	2	2	1	1	4
4	5	4	4	4	4	4	3	3
4	4	4	3	4	5	4	5	5
4	4	5	4	4	4	3	4	4
3	1	1	2	2	1	1	1	4
5	4	5	4	5	5	4	4	4
3	4	4	4	3	4	3	3	5
5	5	3	4	5	4	4	5	5
4	3	3	4	4	4	5	4	1
5	4	4	4	3	3	3	4	4
2	5	4	4	4	4	4	5	1
2	1	2	2	3	2	1	3	4
4	3	3	5	3	4	4	4	4
5	4	5	5	4	3	3	4	4

3	3	3	4	4	5	3	3	4
1	4	3	3	4	5	4	3	4
3	3	5	5	4	4	4	4	5
5	4	5	4	4	4	3	4	4
4	5	4	4	5	5	5	4	4
1	3	3	4	5	4	4	4	2
3	3	5	4	3	3	4	3	4
2	3	3	1	2	1	3	2	4
5	3	4	3	4	4	4	4	4
4	5	3	4	3	4	4	5	5
4	4	5	5	5	4	3	4	3
4	5	4	4	3	5	4	4	3
2	2	2	3	3	1	2	1	4
4	5	4	3	5	5	4	4	3
1	3	5	3	5	4	3	4	3
3	3	5	4	5	4	5	3	4
4	4	5	4	4	3	4	5	4
4	3	4	4	3	5	3	3	4
3	3	5	4	4	5	4	4	5
4	3	3	4	4	3	4	4	5
4	5	5	3	3	3	3	5	4
4	3	4	3	4	3	3	4	5
4	3	3	5	4	3	5	3	3
5	5	3	4	4	5	5	3	5
4	5	5	3	4	3	5	4	5
3	4	4	5	4	5	4	3	4
4	4	3	4	4	4	4	4	5
3	1	1	1	2	2	2	1	4
5	3	4	3	5	3	4	3	5
4	5	5	4	3	4	3	5	3
4	4	4	3	3	3	3	5	3
1	2	2	2	3	1	2	2	4
2	1	3	2	3	2	2	2	4
3	4	4	5	5	4	3	4	3
4	3	4	4	3	5	3	4	3
4	1	1	1	1	1	1	1	4
4	4	5	5	4	3	4	3	5
3	4	4	3	5	3	3	3	4
5	5	4	5	5	3	3	4	3
2	2	3	1	2	3	2	1	5
5	5	4	3	4	4	4	4	4
4	3	4	4	3	3	5	5	5
3	3	3	4	5	4	3	4	3
1	2	2	2	1	2	1	2	3
5	3	4	5	4	5	5	4	2
5	4	4	5	4	4	4	4	2
4	3	5	5	5	4	4	5	3

5	4	3	4	5	3	3	4	5
5	4	5	4	4	5	3	3	4
3	5	3	4	4	4	3	5	4
1	2	2	2	2	2	3	2	2
2	1	2	2	3	2	2	1	1
2	2	2	2	2	3	2	3	2
4	4	4	4	3	4	4	3	5
4	5	4	4	3	5	4	4	4
3	3	4	4	4	3	5	3	2
5	4	4	4	3	4	3	5	4
4	5	4	4	4	4	4	4	4
3	3	2	3	1	2	2	2	4
4	4	4	5	4	4	4	4	3
4	5	4	4	3	4	4	4	4
4	4	4	3	4	5	4	3	4
4	4	3	4	4	3	4	3	5
4	3	4	4	4	4	3	3	4
3	5	4	4	4	3	4	4	3
2	3	3	2	2	2	2	2	2
4	3	4	3	4	4	3	4	4
4	4	4	3	5	3	4	3	3
3	3	1	2	3	3	3	2	3
4	3	3	3	4	4	4	3	4
3	4	4	4	4	5	4	5	4
2	2	2	3	2	2	2	2	3
3	3	4	5	4	4	4	3	5
3	5	3	5	4	3	4	3	4
4	4	5	4	4	5	5	3	3
4	4	5	5	4	3	5	4	3
3	4	4	4	3	5	5	5	4
3	4	3	4	3	3	5	4	5
3	3	5	5	4	4	4	4	4
4	3	5	5	3	3	4	4	5
4	4	4	4	3	4	4	3	3
3	3	2	1	3	1	2	2	3
1	5	4	4	3	4	4	4	5
4	4	4	3	5	4	4	4	4
3	4	3	4	4	3	4	3	4
3	4	4	5	4	4	3	5	3
4	5	4	5	4	4	5	4	3
5	5	4	5	3	4	5	4	5
5	4	4	4	4	5	3	4	3
3	1	2	2	2	1	1	1	4
3	5	5	5	5	4	4	3	4
5	5	4	4	4	4	3	4	4
5	4	3	5	3	5	4	4	4
4	3	4	4	4	5	4	3	5

4	3	3	3	5	5	3	3	5
3	2	1	2	2	2	2	1	4
3	4	5	4	4	4	3	4	5
3	4	4	4	4	5	4	3	5
3	3	5	4	5	4	3	5	4
5	3	5	3	5	5	3	4	4
5	4	5	3	4	4	4	4	2
3	3	4	3	4	4	4	4	5
3	4	4	4	4	4	3	4	5
3	5	4	3	3	4	3	5	3
4	3	4	3	5	4	4	4	3
4	5	5	5	3	3	5	4	4
4	5	4	4	4	4	4	5	4
4	4	4	5	4	5	5	3	3
5	5	4	4	4	4	5	4	3
2	1	1	1	3	1	2	1	3
4	5	4	4	4	4	3	3	4
5	5	3	3	3	5	3	5	5
4	3	3	3	4	3	3	4	5
4	4	5	5	3	4	4	4	4
4	4	4	3	5	4	5	5	5
1	1	3	1	1	1	2	1	3
3	4	4	3	5	4	4	4	5
4	5	5	5	5	4	5	4	5
4	4	5	5	5	4	4	5	3
4	5	3	5	5	3	3	3	4
4	3	3	4	5	4	4	4	5
4	4	4	4	4	5	5	4	4
4	4	4	3	5	4	3	4	4
4	5	4	4	4	4	4	5	5
3	3	2	1	2	2	1	2	4
3	3	4	4	4	5	4	4	1
4	3	4	5	4	4	4	4	4
2	1	2	2	3	3	2	3	2
2	2	3	3	2	2	2	2	1
4	5	5	4	4	4	5	4	4
4	5	4	5	4	5	5	4	4
4	4	5	3	5	4	5	4	4
5	4	4	4	4	5	5	5	3
4	4	3	4	5	5	4	3	4
4	4	4	3	3	5	4	5	5
1	4	5	3	5	5	4	3	3
3	5	4	5	4	3	4	4	4
4	5	3	5	4	4	3	5	3
4	3	4	4	3	3	3	4	5
3	4	5	4	4	5	4	5	5
3	5	5	4	3	3	5	4	5

5	4	5	5	5	4	5	3	5
5	5	3	4	5	4	4	5	4
4	4	3	4	3	3	4	4	4
2	4	4	4	5	4	4	3	1
5	3	4	4	4	3	4	4	4
3	3	2	3	2	3	3	1	4
5	4	4	5	4	4	3	4	4
5	3	4	4	4	3	4	5	4
5	3	3	5	4	3	3	4	4
3	3	4	3	3	3	4	4	4
4	3	5	5	3	4	5	3	3
3	4	3	4	3	3	3	3	4
4	4	5	5	4	4	5	4	4
5	4	3	4	4	5	4	3	3
5	4	4	3	5	4	4	4	5
4	4	4	4	4	4	3	5	4
3	3	2	1	2	2	2	3	1
5	5	4	4	5	4	3	4	4
4	4	4	5	3	5	4	4	5
2	4	3	5	5	5	5	4	1
4	3	4	4	5	3	4	4	4
5	2	2	2	1	1	2	2	4
3	5	3	4	3	4	4	5	3
3	3	3	2	2	2	2	1	2
2	2	1	2	2	1	1	1	3
2	2	1	1	2	3	1	2	3
4	4	4	3	3	5	4	4	5
4	3	3	4	4	3	3	4	5
4	4	4	3	3	4	5	4	5
5	4	4	4	5	4	4	5	4
4	4	4	3	4	4	4	4	4
4	4	4	3	5	4	5	5	4
4	4	5	3	5	3	4	4	4
3	5	4	3	4	4	4	4	4
5	4	5	5	3	4	4	3	4
4	4	5	4	4	5	4	5	4
3	4	3	5	3	3	5	4	5
5	5	5	4	4	4	5	4	5
1	4	3	4	4	4	5	3	1
4	3	4	5	4	4	4	5	4
4	4	4	4	4	3	3	4	3
4	4	4	5	3	4	4	3	5
4	4	4	4	4	4	3	5	3
3	3	3	3	3	5	4	5	3
2	3	2	3	2	3	2	2	5
4	4	4	5	4	4	4	3	3
3	2	1	2	3	2	3	1	1

5	4	5	4	4	4	4	5	5
3	5	5	4	4	3	3	3	3
4	4	4	4	3	4	5	4	3
4	3	4	4	3	4	4	4	4
5	3	4	3	4	5	5	4	4
1	2	2	3	2	1	3	1	2
4	1	2	2	1	1	2	1	4
4	4	4	4	4	4	4	3	4
5	3	4	4	3	4	3	5	3
4	3	3	5	3	5	5	4	4
4	4	4	3	4	4	3	4	4
3	1	2	2	3	1	3	2	2
2	2	2	3	1	3	1	1	2
5	4	3	4	4	3	4	5	3
4	3	3	5	3	5	4	5	4
3	5	5	3	4	4	4	4	5
4	4	3	4	5	4	4	4	3
5	3	4	5	5	5	3	4	3
4	4	4	4	5	4	3	5	4
3	3	3	4	3	5	3	5	4
4	5	3	5	5	4	4	4	4
4	4	5	4	5	4	3	3	4
5	3	3	4	3	4	4	4	3
3	3	3	4	4	5	4	3	3
5	5	3	4	5	4	4	4	3
4	3	4	3	4	5	4	4	4
3	1	1	2	2	1	2	2	2
4	4	5	4	5	4	4	4	5
2	1	2	2	2	3	1	1	3
4	3	5	4	5	4	4	4	5
1	2	3	3	3	2	2	3	4
4	5	4	3	4	5	5	4	5
3	5	3	4	4	3	3	4	4
3	4	5	4	3	4	5	3	3
4	4	3	4	3	3	5	3	5
4	5	5	4	4	5	4	3	4
3	2	2	3	2	3	3	3	4
3	5	5	5	4	5	4	4	4
4	4	3	4	4	4	3	4	4
3	3	2	2	2	2	3	2	2
4	5	3	4	4	5	3	4	5
3	3	5	5	3	4	5	5	3
3	4	4	4	3	5	5	4	3
1	3	5	4	4	4	4	4	4
3	4	5	5	5	4	4	4	3
4	4	3	3	4	4	5	3	3
5	5	5	3	4	5	4	3	5

4	5	4	5	4	3	4	4	4
4	4	3	4	4	4	4	4	4
4	4	4	4	3	4	5	5	4
4	4	4	5	5	4	4	4	4
4	5	4	5	4	3	4	4	4
2	3	3	1	3	1	2	3	2
3	3	3	4	4	4	3	4	4
3	5	3	5	4	3	4	4	4
4	5	4	4	5	4	3	4	3
4	3	5	4	3	5	3	3	4
4	4	4	5	3	3	4	3	5
4	5	5	5	5	3	4	3	5
4	4	4	3	5	3	3	5	5
2	3	4	3	3	3	4	4	5
4	3	3	3	3	3	4	5	4
5	4	3	4	5	4	5	3	4
5	3	3	4	5	4	3	5	4
4	4	3	4	4	5	4	4	4
5	3	3	4	4	3	5	3	3
3	3	5	3	3	5	3	4	4
3	3	5	5	4	5	4	4	3
5	4	4	3	4	3	4	3	5
2	3	2	1	1	2	1	2	1
2	2	2	2	2	2	3	1	2
4	4	3	5	3	4	4	4	4
5	3	4	3	3	5	3	3	5
5	3	4	3	5	4	3	3	3
5	4	5	3	4	5	4	5	3
4	4	4	5	5	3	4	4	4
5	5	5	5	4	4	5	4	4
2	1	1	2	1	1	2	1	1
2	2	3	2	3	1	3	3	2
4	3	4	3	3	4	5	4	4
4	5	5	4	4	4	4	4	5
4	3	3	4	4	4	4	3	3
5	4	3	5	3	4	4	3	4
3	1	3	1	1	2	2	1	1
4	5	4	5	5	3	5	3	3
4	3	4	4	5	4	3	3	3
2	2	2	1	1	2	2	1	1
3	4	5	4	4	3	4	3	5
4	3	4	3	4	5	4	5	4
3	3	5	3	5	4	5	4	4
5	4	4	3	5	3	4	4	4
4	5	3	5	4	3	4	4	4
3	2	2	1	3	1	1	2	1
3	4	5	5	4	4	3	3	5

3	5	4	3	3	4	4	4	4
3	5	5	4	5	5	5	4	4
4	4	5	5	4	4	4	4	3
5	4	4	4	4	3	3	4	3
2	2	2	2	3	1	3	2	3
5	3	4	5	5	3	5	3	4
4	5	3	4	5	5	5	5	5
5	4	4	5	4	4	4	4	4
5	4	3	4	4	3	4	3	5
4	4	4	5	3	4	4	4	5
5	3	4	4	4	4	4	4	4
4	4	4	3	4	4	4	4	4
3	4	3	5	4	3	5	4	4
2	3	1	3	2	2	3	2	4
2	3	1	2	2	1	2	3	4
5	5	5	5	5	4	4	4	3
4	4	4	5	4	5	3	5	5
4	4	4	5	3	4	4	4	4
5	3	4	4	4	4	4	5	3
3	3	4	5	5	4	3	4	4
5	4	4	4	4	4	4	4	5
5	4	4	5	3	4	5	4	3
4	4	4	4	5	3	5	4	4
4	4	4	3	4	4	3	4	4
4	3	4	3	4	4	3	3	3
5	3	4	3	5	5	3	5	3
4	4	4	4	4	5	4	4	4
3	4	3	5	5	4	5	4	4
4	4	4	5	5	5	4	5	4
1	1	2	2	1	2	2	1	5
5	3	3	4	5	3	4	3	4
4	3	5	4	5	5	5	5	4
3	4	3	5	5	3	5	5	4
4	2	1	2	2	1	1	2	5
3	5	5	4	3	4	5	4	4
4	2	2	1	2	2	1	1	4
2	4	3	3	3	5	5	4	1
5	3	5	5	4	4	3	3	4
3	4	4	4	5	4	5	5	3
5	4	5	4	4	3	4	4	3
5	3	4	3	4	4	3	4	4
3	5	4	3	5	5	4	4	4
3	1	2	1	1	2	1	2	2
3	2	3	1	3	2	2	1	3
5	5	3	3	4	4	4	3	5
2	3	3	1	1	1	2	2	4
4	5	4	4	4	4	4	3	4

4	4	4	5	4	4	4	3	4
5	3	4	5	3	4	4	5	4
5	5	3	5	5	3	4	3	4
2	2	2	2	1	3	3	2	3
3	5	4	4	3	3	4	4	3
5	4	5	4	3	5	5	4	3
4	2	2	1	2	2	2	2	4
3	4	4	5	3	5	4	5	5
4	5	3	3	5	5	4	4	4
4	1	2	1	1	2	2	2	3
4	4	3	3	4	4	5	4	4
5	5	5	3	4	5	3	3	3
2	4	4	5	4	4	3	4	1
4	3	5	3	4	3	4	4	4
3	5	4	5	3	4	5	4	4
5	5	4	4	4	3	4	4	5
4	3	3	5	4	4	4	3	3
5	3	3	3	4	5	3	4	3
4	5	3	4	5	5	4	5	4
4	4	4	5	3	4	5	4	4
4	5	5	5	4	5	3	5	3
2	3	1	2	1	2	2	1	2
4	4	5	3	3	4	5	4	5
4	3	5	4	4	4	4	5	4
4	5	3	3	4	3	4	4	3
2	2	2	1	1	3	2	2	4
1	4	4	5	4	5	3	4	2
3	2	1	3	1	1	1	1	2
4	4	5	3	4	3	4	5	4
4	4	4	4	3	5	4	3	3
4	5	5	3	4	4	5	4	4
3	4	3	5	4	5	4	4	5
5	3	4	5	4	5	3	4	3
5	2	1	1	2	2	1	2	4
4	5	5	3	3	3	4	5	4
1	3	3	1	2	2	2	3	4
4	4	4	5	3	4	4	5	4
3	3	5	4	4	4	4	4	5
4	5	4	5	5	4	4	4	4
4	4	3	4	4	4	4	3	5
3	1	1	2	2	3	3	3	2
4	1	1	2	1	1	1	2	2
4	4	3	3	3	5	4	5	3
3	3	4	5	4	4	3	4	5
4	5	4	5	4	4	5	5	5
4	4	4	4	4	4	5	3	3
3	5	5	5	4	4	5	3	4

2	3	3	2	1	2	3	3	4
3	4	4	4	5	3	4	5	1
4	5	3	3	4	5	3	4	3
4	3	5	3	4	4	4	3	5
5	4	3	4	5	3	4	4	5
3	5	4	4	5	3	3	5	4
4	3	4	4	4	5	4	3	4
5	4	3	5	4	3	4	4	3
4	3	4	4	3	4	3	3	3
3	4	4	3	4	5	5	5	4
4	4	3	3	3	5	4	4	4
4	4	4	5	5	3	4	4	4
5	4	4	4	5	5	4	4	5
4	4	4	4	4	4	4	3	3
4	4	4	4	4	5	4	5	5
3	3	4	4	3	3	5	4	4
5	1	1	1	2	2	2	2	4
4	3	4	4	4	4	3	4	4
3	3	5	5	4	4	3	5	4
4	3	4	4	4	3	3	5	4
3	5	3	3	5	4	5	3	4
4	4	3	5	5	4	3	4	5
5	5	4	4	3	3	3	3	3
1	1	1	2	2	2	2	2	1
3	4	4	4	3	5	3	4	3
4	1	2	1	1	1	1	1	3
1	3	3	3	2	3	2	3	4
4	5	3	4	3	4	4	4	5
3	5	5	4	4	4	5	5	2
1	1	2	1	3	1	3	1	4
4	4	5	4	3	5	3	4	4
5	5	4	3	4	4	4	4	5
1	4	3	5	4	4	3	4	1
1	5	4	5	4	4	4	3	2
5	4	5	4	3	3	4	4	4
4	5	5	5	4	3	4	3	5
4	5	3	4	4	3	3	4	1
5	4	4	4	3	4	5	4	4
3	5	3	4	5	3	3	4	3
4	3	4	3	5	3	4	4	3
3	4	4	4	3	5	4	4	4
3	2	2	2	1	3	2	1	3
3	5	5	5	4	3	5	4	4
3	3	5	4	5	4	4	4	4
4	4	5	4	4	3	4	4	4
4	3	4	4	4	3	3	5	4
2	5	5	4	4	5	5	4	2

3	5	5	5	3	3	4	4	4
3	4	5	4	5	4	5	4	4
3	4	4	4	4	4	3	4	3
5	5	4	5	4	5	4	4	3
4	4	3	5	4	4	4	4	5
4	5	3	4	5	3	3	3	4
4	5	4	4	5	5	3	3	3
3	1	2	2	2	1	1	2	4
3	2	2	2	3	2	3	3	2
4	5	3	3	5	5	3	4	4
4	4	4	4	3	5	4	5	3
4	4	3	3	5	5	5	3	5
4	4	4	3	4	4	5	3	5
3	3	4	4	4	5	3	5	5
1	4	4	5	3	4	4	3	1
4	5	4	3	5	4	3	3	5
3	3	4	4	5	4	4	4	4
3	4	4	4	5	4	4	3	3
5	5	4	3	3	5	5	3	4
4	3	5	5	5	5	4	4	4
1	1	1	2	3	1	2	1	3
4	4	4	4	4	3	4	3	2
5	4	5	3	4	4	4	4	4
4	5	5	5	5	5	5	5	5
5	1	2	1	2	2	1	1	4
4	5	4	4	4	4	4	3	3
4	4	4	3	4	5	4	5	5
4	4	5	4	4	4	3	4	4
3	1	1	2	2	1	1	1	4
5	4	5	4	5	5	4	4	4
3	4	4	4	3	4	3	3	5
5	5	3	4	5	4	4	5	5
4	3	3	4	4	4	5	4	1
5	4	4	4	3	3	3	4	4
2	5	4	4	4	4	4	5	1
2	1	2	2	3	2	1	3	4
4	3	3	5	3	4	4	4	4
5	4	5	5	4	3	3	4	4
3	3	3	4	4	5	3	3	4
1	4	3	3	4	5	4	3	4
3	3	5	5	4	4	4	4	5
5	4	5	4	4	4	3	4	4
4	5	4	4	5	5	5	4	4
1	3	3	4	5	4	4	4	2
3	3	5	4	3	3	4	3	4
2	3	3	1	2	1	3	2	4
5	3	4	3	4	4	4	4	4

4	5	3	4	3	4	4	5	5
4	4	5	5	5	4	3	4	3
4	5	4	4	3	5	4	4	3
2	2	2	3	3	1	2	1	4
4	5	4	3	5	5	4	4	3
1	3	5	3	5	4	3	4	3
3	3	5	4	5	4	5	3	4
4	4	5	4	4	3	4	5	4
4	3	4	4	3	5	3	3	4
3	3	5	4	4	5	4	4	5
4	3	3	4	4	3	4	4	5
4	5	5	3	3	3	3	5	4
4	3	4	3	4	3	3	4	5
4	3	3	5	4	3	5	3	3
5	5	3	4	4	5	5	3	5
4	5	5	3	4	3	5	4	5
3	4	4	5	4	5	4	3	4
4	4	3	4	4	4	4	4	5
3	1	1	1	2	2	2	1	4
5	3	4	3	5	3	4	3	5
4	5	5	4	3	4	3	5	3
4	4	4	3	3	3	3	5	3
1	2	2	2	3	1	2	2	4
2	1	3	2	3	2	2	2	4
3	4	4	5	5	4	3	4	3
4	3	4	4	3	5	3	4	3
4	1	1	1	1	1	1	1	4
4	4	5	5	4	3	4	3	5
3	4	4	3	5	3	3	3	4
5	5	4	5	5	3	3	4	3
2	2	3	1	2	3	2	1	5
5	5	4	3	4	4	4	4	4
4	3	4	4	3	3	5	5	5
3	3	3	4	5	4	3	4	3
1	2	2	2	1	2	1	2	3
5	3	4	5	4	5	5	4	2
5	4	4	5	4	4	4	4	2
4	3	5	5	5	4	4	5	3
5	4	3	4	5	3	3	4	5
5	4	5	4	4	5	3	3	4
3	5	3	4	4	4	3	5	4
1	2	2	2	2	2	3	2	2
2	1	2	2	3	2	2	1	1
2	2	2	2	2	3	2	3	2
4	4	4	4	3	4	4	3	5
4	5	4	4	3	5	4	4	4
3	3	4	4	4	3	5	3	2

5	4	4	4	3	4	3	5	4
4	5	4	4	4	4	4	4	4
3	3	2	3	1	2	2	2	4
4	4	4	5	4	4	4	4	3
4	5	4	4	3	4	4	4	4
4	4	4	3	4	5	4	3	4
4	4	3	4	4	3	4	3	5
4	3	4	4	4	4	3	3	4
3	5	4	4	4	3	4	4	3
2	3	3	2	2	2	2	2	2
4	3	4	3	4	4	3	4	4
4	4	4	3	5	3	4	3	3
3	3	1	2	3	3	3	2	3
4	3	3	3	4	4	4	3	4
3	4	4	4	4	5	4	5	4
2	2	2	3	2	2	2	2	3
3	3	4	5	4	4	4	3	5
3	5	3	5	4	3	4	3	4
4	4	5	4	4	5	5	3	3
4	4	5	5	4	3	5	4	3
3	4	4	4	3	5	5	5	4
3	4	3	4	3	3	5	4	5
3	3	5	5	4	4	4	4	4
4	3	5	5	3	3	4	4	5
4	4	4	4	3	4	4	3	3
3	3	2	1	3	1	2	2	3
1	5	4	4	3	4	4	4	5
4	4	4	3	5	4	4	4	4
3	4	3	4	4	3	4	3	4
3	4	4	5	4	4	3	5	3
4	5	4	5	4	4	5	4	3
5	5	4	5	3	4	5	4	5
5	4	4	4	4	5	3	4	3
3	1	2	2	2	1	1	1	4
3	5	5	5	5	4	4	3	4
5	5	4	4	4	4	3	4	4
5	4	3	5	3	5	4	4	4
4	3	4	4	4	5	4	3	5
4	3	3	3	5	5	3	3	5
3	2	1	2	2	2	2	1	4
3	4	5	4	4	4	3	4	5
3	4	4	4	4	5	4	3	5
3	3	5	4	5	4	3	5	4
5	3	5	3	5	5	3	4	4
5	4	5	3	4	4	4	4	2
3	3	4	3	4	4	4	4	5
3	4	4	4	4	4	3	4	5

3	5	4	3	3	4	3	5	3
4	3	4	3	5	4	4	4	3
4	5	5	5	3	3	5	4	4
4	5	4	4	4	4	4	5	4
4	4	4	5	4	5	5	3	3
5	5	4	4	4	4	5	4	3
2	1	1	1	3	1	2	1	3
4	5	4	4	4	4	3	3	4
5	5	3	3	3	5	3	5	5
4	3	3	3	4	3	3	4	5
4	4	5	5	3	4	4	4	4
4	4	4	3	5	4	5	5	5
1	1	3	1	1	1	2	1	3
3	4	4	3	5	4	4	4	5
4	5	5	5	5	4	5	4	5
4	4	5	5	5	4	4	5	3
4	5	3	5	5	3	3	3	4
4	3	3	4	5	4	4	4	5
4	4	4	4	4	5	5	4	4
4	4	4	3	5	4	3	4	4
4	5	4	4	4	4	4	5	5
3	3	2	1	2	2	1	2	4
3	3	4	4	4	5	4	4	1
4	3	4	5	4	4	4	4	4
2	1	2	2	3	3	2	3	2
2	2	3	3	2	2	2	2	1
4	5	5	4	4	4	5	4	4
4	5	4	5	4	5	5	4	4
4	4	5	3	5	4	5	4	4
5	4	4	4	4	5	5	5	3
4	4	3	4	5	5	4	3	4
4	4	4	3	3	5	4	5	5
1	4	5	3	5	5	4	3	3
3	5	4	5	4	3	4	4	4
4	5	3	5	4	4	3	5	3
4	3	4	4	3	3	3	4	5
3	4	5	4	4	5	4	5	5
3	5	5	4	3	3	5	4	5
5	4	5	5	5	4	5	3	5
5	5	3	4	5	4	4	5	4
4	4	3	4	3	3	4	4	4
2	4	4	4	5	4	4	3	1
5	3	4	4	4	3	4	4	4
3	3	2	3	2	3	3	1	4
5	4	4	5	4	4	3	4	4
5	3	4	4	4	3	4	5	4
5	3	3	5	4	3	3	4	4

3	3	4	3	3	3	4	4	4
4	3	5	5	3	4	5	3	3
3	4	3	4	3	3	3	3	4
4	4	5	5	4	4	5	4	4
5	4	3	4	4	5	4	3	3
5	4	4	3	5	4	4	4	5
4	4	4	4	4	4	3	5	4
3	3	2	1	2	2	2	3	1
5	5	4	4	5	4	3	4	4
4	4	4	5	3	5	4	4	5
2	4	3	5	5	5	5	4	1
4	3	4	4	5	3	4	4	4
5	2	2	2	1	1	2	2	4
3	5	3	4	3	4	4	5	3
3	3	3	2	2	2	2	1	2
2	2	1	2	2	1	1	1	3
2	2	1	1	2	3	1	2	3
4	4	4	3	3	5	4	4	5
4	3	3	4	4	3	3	4	5
4	4	4	3	3	4	5	4	5
5	4	4	4	5	4	4	5	4
5	5	3	4	5	4	4	5	4
4	4	3	4	3	3	4	4	4
2	4	4	4	5	4	4	3	1
5	3	4	4	4	3	4	4	4
3	3	2	3	2	3	3	1	4
5	4	4	5	4	4	3	4	4
5	3	4	4	4	3	4	5	4
5	3	3	5	4	3	3	4	4
3	3	4	3	3	3	4	4	4
4	3	5	5	3	4	5	3	3
3	4	3	4	3	3	3	3	4
4	4	5	5	4	4	5	4	4
5	4	3	4	4	5	4	3	3
5	4	4	3	5	4	4	4	5
4	4	4	4	4	4	3	5	4
3	3	2	1	2	2	2	3	1
5	5	4	4	5	4	3	4	4
4	4	4	5	3	5	4	4	5
2	4	3	5	5	5	5	4	1
4	3	4	4	5	3	4	4	4
5	2	2	2	1	1	2	2	4
3	5	3	4	3	4	4	5	3
3	3	3	2	2	2	2	1	2
2	2	1	2	2	1	1	1	3
2	2	1	1	2	3	1	2	3
4	4	4	3	3	5	4	4	5

4	3	3	4	4	3	3	4	5
4	4	4	3	3	4	5	4	5
5	4	4	4	5	4	4	5	4
5	5	3	4	5	4	4	5	4
4	4	3	4	3	3	4	4	4
2	4	4	4	5	4	4	3	1
5	3	4	4	4	3	4	4	4
3	3	2	3	2	3	3	1	4
5	4	4	5	4	4	3	4	4
5	3	4	4	4	3	4	5	4
5	3	3	5	4	3	3	4	4
3	3	4	3	3	3	4	4	4
4	3	5	5	3	4	5	3	3
3	4	3	4	3	3	3	3	4
4	4	5	5	4	4	5	4	4
5	4	3	4	4	5	4	3	3
5	4	4	3	5	4	4	4	5
4	4	4	4	4	4	3	5	4
3	3	2	1	2	2	2	3	1
5	5	4	4	5	4	3	4	4
4	4	4	5	3	5	4	4	5
2	4	3	5	5	5	5	4	1
4	3	4	4	5	3	4	4	4
5	2	2	2	1	1	2	2	4
3	5	3	4	3	4	4	5	3
3	3	3	2	2	2	2	1	2
2	2	1	2	2	1	1	1	3
2	2	1	1	2	3	1	2	3
4	4	4	3	3	5	4	4	5
4	3	3	4	4	3	3	4	5
4	4	4	3	3	4	5	4	5
5	4	4	4	5	4	4	5	4
5	5	3	4	5	4	4	5	4
4	4	3	4	3	3	4	4	4
2	4	4	4	5	4	4	3	1
5	3	4	4	4	3	4	4	4
3	3	2	3	2	3	3	1	4
5	4	4	5	4	4	3	4	4
5	3	4	4	4	3	4	5	4
5	3	3	5	4	3	3	4	4
3	3	4	3	3	3	4	4	4
4	3	5	5	3	4	5	3	3
3	4	3	4	3	3	3	3	4
4	4	5	5	4	4	5	4	4
5	4	3	4	4	5	4	3	3
5	4	4	3	5	4	4	4	5
4	4	4	4	4	4	3	5	4

3	3	2	1	2	2	2	3	1
5	5	4	4	5	4	3	4	4
4	4	4	5	3	5	4	4	5
2	4	3	5	5	5	5	4	1
4	3	4	4	5	3	4	4	4
5	2	2	2	1	1	2	2	4
3	5	3	4	3	4	4	5	3
3	3	3	2	2	2	2	1	2
2	2	1	2	2	1	1	1	3
2	2	1	1	2	3	1	2	3
4	4	4	3	3	5	4	4	5
4	3	3	4	4	3	3	4	5
4	4	4	3	3	4	5	4	5
5	4	4	4	5	4	4	5	4
3	3	3	2	2	2	2	1	2
2	2	1	2	2	1	1	1	3
2	2	1	1	2	3	1	2	3
4	4	4	3	3	5	4	4	5
4	3	3	4	4	3	3	4	5
4	4	4	3	3	4	5	4	5
5	4	4	4	5	4	4	5	4
3	3	3	2	2	2	2	1	2
2	2	1	2	2	1	1	1	3
2	2	1	1	2	3	1	2	3
4	4	4	3	3	5	4	4	5
2	2	1	1	2	3	1	2	3
